# Delineation of Pathogenomic Insights of Breast Cancer in Young Women

**DOI:** 10.3390/cells11121927

**Published:** 2022-06-15

**Authors:** Aswathy Mary Paul, Bijesh George, Sunil Saini, Madhavan Radhakrishna Pillai, Masakazu Toi, Luis Costa, Rakesh Kumar

**Affiliations:** 1 Cancer Research Program, Rajiv Gandhi Centre for Biotechnology, Thiruvananthapuram 695014, India; aswathym@rgcb.res.in (A.M.P.); bijeshgeorge@rgcb.res.in (B.G.); 2 Graduate Program, Manipal Academy of Higher Education, Manipal 576104, India; 3 Cancer Research Institute, Himalayan Institute of Medical Sciences, Swami Rama Himalayan University, Dehradun 248016, India; sunilsaini@srhu.edu.in; 4 SAGENOME Private Limited, BioNest, Kochi 683503, India; mrpillai@ohmygene.com; 5 Graduate School of Medicine, Kyoto University, Kyoto 606-8507, Japan; toi@kuhp.kyoto-u.ac.jp; 6 Department of Medical Oncology, Hospital de Santa Maria, Centro Hospitalar Universitário Lisboa Norte, 1649-028 Lisbon, Portugal; lmcosta@medicina.ulisboa.pt; 7 Instituto de Medicina Molecular-João Lobo Antunes, Faculdade de Medicina da Universidade de Lisboa, 1649-028 Lisboa, Portugal; 8 Department of Human and Molecular Genetics, School of Medicine, Virginia Commonwealth University, Richmond, VA 23298, USA; 9 Division of Hematology and Oncology, Department of Medicine, Rutgers New Jersey Medical School, Newark, NJ 07103, USA

**Keywords:** early breast cancer, genomics, transcriptomics, prognosis, predictive biomarkers, young women

## Abstract

The prognosis of breast cancer (BC) in young women (BCYW) aged ≤40 years tends to be poorer than that in older patients due to aggressive phenotypes, late diagnosis, distinct biologic, and poorly understood genomic features of BCYW. Considering the estimated predisposition of only approximately 15% of the BC population to BC-promoting genes, the underlying reasons for an increased occurrence of BCYW, at large, cannot be completely explained based on general risk factors for BC. This underscores the need for the development of next-generation of tissue- and body fluid-based prognostic and predictive biomarkers for BCYW. Here, we identified the genes associated with BCYW with a particular focus on the age, intrinsic BC subtypes, matched normal or normal breast tissues, and BC laterality. In young women with BC, we observed dysregulation of age-associated cancer-relevant gene sets in both cancer and normal breast tissues, sub-sets of which substantially affected the overall survival (OS) or relapse-free survival (RFS) of patients with BC and exhibited statically significant correlations with several gene modules associated with cellular processes such as the stroma, immune responses, mitotic progression, early response, and steroid responses. For example, high expression of COL1A2, COL5A2, COL5A1, NPY1R, and KIAA1644 mRNAs in the BC and normal breast tissues from young women correlated with a substantial reduction in the OS and RFS of BC patients with increased levels of these exemplified genes. Many of the genes upregulated in BCYW were overexpressed or underexpressed in normal breast tissues, which might provide clues regarding the potential involvement of such genes in the development of BC later in life. Many of BCYW-associated gene products were also found in the extracellular microvesicles/exosomes secreted from breast and other cancer cell-types as well as in body fluids such as urine, saliva, breast milk, and plasma, raising the possibility of using such approaches in the development of non-invasive, predictive and prognostic biomarkers. In conclusion, the findings of this study delineated the pathogenomics of BCYW, providing clues for future exploration of the potential predictive and prognostic importance of candidate BCYW molecules and research strategies as well as a rationale to undertake a prospective clinical study to examine some of testable hypotheses presented here. In addition, the results presented here provide a framework to bring out the importance of geographical disparities, to overcome the current bottlenecks in BCYW, and to make the next quantum leap for sporadic BCYW research and treatment.

## 1. Introduction

Breast cancer (BC) is the fifth leading cause of cancer-related deaths worldwide, with an approximately 8–12% estimated lifetime risk [1]. Although the BC-associated mortality rate has reduced in several countries, this gain has not proportionately benefited young women with BC in many parts of the world [2,3,4]. The operational definition of BC in young women (BCYW) is generally flexible because it has been applied to BC in women aged <35, <40, or <50 years or even to BC in premenopausal women [4]. In this study, we defined BCYW as BC in women aged ≤40 years according to a recent consensus based on the clinical features of tumors [5]. In general, age is considered as a risk factor for human cancer due to cumulative dysregulated changes occurring with age in the genome and associated pathways leading to the loss of cellular homeostasis. However, the current trend of a high incidence of aggressive BC in women aged ≤40 or ≤35 years argues against the validity of growing age as a risk factor for BCYW. The prevalence of BCYW is approximately 2–6% in Western countries and 10–20% in Asian countries [6], suggesting that BCYW has become a growing concern globally, including in countries with a vast young population such as India.

The range of clinical characteristics exhibited in BCYW is distinct from that exhibited in older premenopausal and postmenopausal women with BC; thus, BCYW could be considered a subset of premenopausal diseases. BCYW exhibits more aggressive cancer subtypes (such as TNBC/basal, HER2-positive, and luminal B) than BC in older patients [7,8,9,10,11,12,13,14]. The prognosis of BCYW is generally worse than that of BC in older patients, presumably due to its aggressive subtypes, detection at an advanced stage, and a high recurrence rate [1,6,15,16,17,18,19]. The occurrence of breast cancer early in life also translates into many social, economic, psychologic, and medical issues, including a relatively early disease relapse as compared to breast cancer in older age groups, even after a long tumor dormancy. The reasons for a poor prognosis of BCYW in most cases continue to be poorly understood but are likely to involve the dysregulation of genomic and associated cellular and stromal interactions.

Regarding the genomics of BCYW beyond mutations in specific predisposed familial genes, numerous studies over the years have described the nature of transcriptomic changes in BCYW aged 35–45 years. These studies have reported changes in the expression of specific genes, gene sets, or signatures in young patients as compared with those in older patients with BC. For example, patients aged <35 years with ER-positive BC showed an upregulation of signatures pertaining to the insulin-like growth factor receptor and stromal microenvironment in luminal A BC subtype [20]. Patients aged <45 years with BC showed an increased expression of growth factors and cell cycle progression genes [21]; patients aged <45 years showed a high prevalence of the GATA3 mutation [22]; those aged <35 years showed an increased expression and/or mutations of GATA3 and ARID1A [23]; and patients aged <40 years showed an increased expression of RANKL [24]. In addition, 38 age-associated genes were identified in normal tissues but not in matched tumor samples [25]; 63 differentially expressed genes were noted in BC patients aged ≤45 years as compared to those aged > 55 years [26]; 50 differentially expressed genes in young BC patients, aged ≤45 years, as compared to BC patients over ≥65 years old [27]; a defective TGF-β pathway and an activated immune pathway were noted in patients with BC aged <40 years [28], etc. In brief, many transcriptomic studies on BCYW have yielded new information on genomic alterations in BCYW; however, a distinct gene set or sets of high clinical significance, or predictive tissue or body fluid-based biomarkers for BCYW and/or for general population screening, remain to be identified.

The increased incidence of relapse in BCYW is also likely to be profoundly affected by pathways involved in the regulation of tumor dormancy and bi-directional tumor–stroma interactions. This aspect of BC research has been generally understudied, particularly in the context of hormone receptors, nuclear estrogen signaling, and non-genomic estrogen signaling. Many studies in the literature have focused on the significance of stromal cell types in the modification of BC phenotypes [29]. For example, a study on age-associated genes derived from normal breast tissues suggested that a subset of aggressive breast tumors in young patients expresses genes that are upregulated in young normal breast tissues [30]. However, the reason for such upregulation and the significance of tumor-associated genes in normal breast tissues remain unknown. Similarly, another large study demonstrated that 1408 and 1150 upregulated and downregulated genes, respectively, were present in the normal breast tissues from donors aged 27–66 years without a history of breast cancer and indicated the significance of adipogenesis and inflammation in BC progression [31].

Because the nature of the mammary gland stroma, in both normal and cancerous tissues, is known to be markedly modified by ovarian hormones, growth factors, and soluble factors [32,33], the genomic landscape of the normal mammary gland (as well as breast tumors) is presumed to be further modified by the menstrual cycle phases, breast-feeding status, and pregnancy phase - all of which are components of women’s reproductive years. In this context, a genomic study described the nature of differentially expressed genes (DEGs) during the luteal and follicular stages of the menstrual cycle in the normal mammary glands of young women. This study revealed the upregulation of 221 genes in the luteal phase, many of which are known to have established functions in BC progression [34]. These examples of genomic studies on normal breast tissues highlight the significance of altered expression of genes or gene sets in the biology of the mammary gland and BC. However, these studies have not considered issues pertaining to BCYW ≤ 40 years. Therefore, the present study was undertaken to delineate the nature of transcriptomic alterations in BC and its four subtypes in young women aged ≤40 years compared with BC in the age groups of 41–54 and ≥55 years to determine the nature of genomic overlaps between age-specific unique genes in BCYW and matched adjacent normal or normal breast tissues and to explore changes in the function of BC subtypes and BC laterality by using BC genomic datasets.

## 2. Materials and Methods

### 2.1. Identification of Age Groups’ Specific Patterns of Gene Expression

We have selected the two largest annotated breast cancer datasets in the public domain for our analysis, the TCGA Breast Invasive Carcinoma Firehose Legacy and METABRIC datasets. First, we downloaded the clinical information for the TCGA breast cancer dataset from the Xena browser [35]. Using the downloaded clinical data, normal and tumor samples were classified into three main age groups: ≤40 years old (26–40 years, mean age: 36.19 years ± 3.71 SD), 41 to 54 years old (mean age: 48.10 years ± 4.01 SD), or ≥55 years old (55–90 years, mean age: 67.27 years ± 8.92 SD) (Supplementary Table S1). The samples under the three age groups were again subdivided into breast cancer subtype groups, Basal, HER2-positive (HER2), Luminal A (LumA), and Luminal B (LumB). According to anatomic neoplasm division information, we also separated the curated samples for each age group into left and right groups. After the sample classification, we performed differential expression analysis between the tumor and normal samples using the TCGA Biolinks, an R package [36]. In addition to the TCGA normal-tumor analysis, we also performed a *t*-test evaluation of a normal–normal analysis between the main age groups using the Limma-R package [37]. We downloaded the breast cancer Illumina HiSeqlog (x + 1), transformed RSEM normalized data from the Xena browser, and extracted the normal sample dataset based on the sample ID for this analysis. The extracted normal data were then classified into three main groups based on the age information and used for the comparative analysis. Comparative analysis among different datasets was performed using a Venn diagram web-based tool [38].

On the second set of analyses using the METABRIC datasets [39], the differential expression analysis was performed using a similar analysis strategy to that used with the TCGA dataset. As opposed to TCGA data, the METABRIC dataset is low throughput data. Based on the age of breast cancer patients at diagnosis as present in the provided clinical data, the METABRIC breast tumor and normal sample list were first divided into three main age groups. We downloaded the normalized expression data for tumors (EGAD00010000210, EGAD00010000211) and normal samples (EGAD00010000212) of the METABRIC dataset under the study ID EGAS00000000083 from The European Genome-phenome Archive (EGA) after receiving the access from the Data Access Committee (DAC). The data were downloaded using the pyEGA3, EGA download client links. We then classified the downloaded data into different age groups using the sample IDs followed by differential expression analysis using the Limma-R package. The results obtained from the differential expression analysis were first reviewed based on the *p*-value. Genes with a *p*-value less than 0.05 were shortlisted for further analysis. The shortlisted significant genes were then classified into upregulated and downregulated ones based on a log fold change value with a 1-fold cut-off (as well as a 2-fold cut-off). Following the differential expression analysis, we performed a detailed overlap analysis to identify shared and unique genes among the different age groups. Our overlap analysis led to identifying a few essential genes, whose expression was further verified using breast cancer datasets in the cBioPortal platform [40].

### 2.2. MRNA–Protein Correlation Analysis

To validate the expression of 60 genes uniquely upregulated in breast tumors from patients aged ≤40 years from differential expression analysis, we focused the TCGA breast invasive carcinoma database and selected the mRNA expression z-scores relative to diploid samples (RNA Seq V2 RSEM) and protein level z-scores (mass spectrometry by CPTAC) with ±2 as the expression cut-off value. After removing the genes with no expression of mRNA or protein, we selected 15 genes with data at the levels of both mRNA and protein as an example of mRNA–protein correlation and presented the expression pattern as a heatmap.

### 2.3. Prognostic Significance

Selected genes from the differential expression analysis were used to determine the prognostic significance. We performed a multivariate analysis using the GOBO and KMPlot online platforms. Using KMPlot, we performed an overall survival analysis of breast cancer patients using the breast cancer dataset (*n* = 1090) and the mean expression of the selected genes [41]. The clinical significance of genes of interest was assessed using the Gene Expression-Based Outcome for Breast Cancer Online (GOBO) platform. The dataset contains Affymetrix-derived gene expression data from 1881 breast tumors (ER+ tumors, n = 1225; ER- tumors, n = 395; untreated tumors, n = 927; and TAM-treated tumors, n = 326), organized into 19 subtypes and untreated and tamoxifen-treated tumors [42]. The expression of the gene set of interest was stratified into high and low levels and analyzed as Kaplan–Meier curves for overall survival (OS), relapse-free survival (RFS), or distant metastasis-free survival (DMFS) over a period of 10 years. To assess the significance of a gene set, the GOBO platform correlates the test gene set against 8 co-expressed gene modules such as stroma, lipid, immune response, mitotic checkpoint, mitotic progression, basal, early response, and steroid response, and plots them as Spearman’s rank correlations [43]. All survival plots were generated using the online GOBO tools.

### 2.4. Gene Ontology Analysis

Gene ontology analysis for biological processes was performed using the FunRich version 3.1.3 functional enrichment analysis on-line tools [44]. The top 10 biological processes were reported in the Supplementary Tables S2–S8 based on the ascending order of *p*-value.

### 2.5. Fitness Analysis

Fitness scores for breast carcinoma cell lines were collected for each gene from the cancer dependency map [45,46] and the boxplots were generated using the ggplot2 library in the R programming language.

### 2.6. Secreted Proteins

We also the possibility of the presence of dysregulated gene products secreted from our different age-specific gene analyses. We predominantly used the Vesiclepedia and plasma proteome databases [47,48,49].

## 3. Results

### 3.1. Identification of Dysregulated Genes in BCYW Aged ≤40 Years

**Figure 1:**
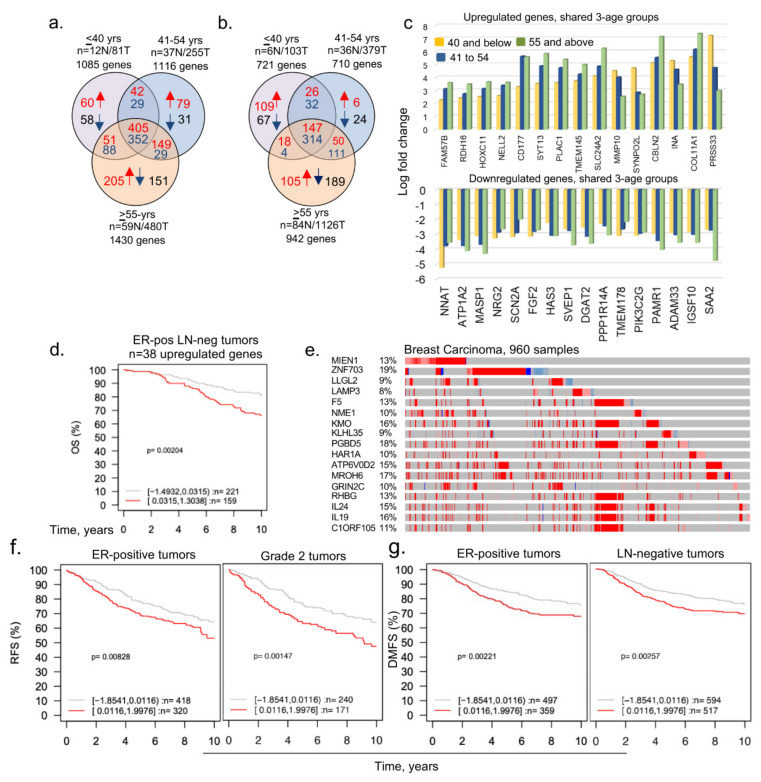
Dysregulated age-associated genes in three age groups of breast cancer patients. (**a**) Curated data from the TCGA Firehose Legacy breast cancer study was segregated into indicated three age groups and transcripts with a cut-off of log 2-fold change are shown here as upregulated or downregulated genes; (**b**) curated data from the METABRIC breast cancer study were divided into three age groups and transcripts with a cut-off of log 1-fold change are shown; (**c**) bar chart representing upregulated (upper panel) and downregulated (lower panel) shared genes among ≤40, 41 to 54 years, and ≥55 years age groups (upper); (**d**) overall survival of ER-positive (ER-pos) lymph-node negative (LN-neg) breast cancer patients using 38 upregulated mapped genes (out of 60 genes) unique in ≤40 years age group. Tumor samples stratified into high (red curve) and low (gray curve) expression of the genes; (**e**) expression of shortlisted 17 highly overexpressed genes. The percentage adjacent to the gene symbol represents the alteration rate. The data was curated from the cBioPortal platform; (**f**,**g**) among the 17 overexpressed genes, 11 genes were mapped in the GOBO database and used for the survival analyses. Relapse-free survival (RFS) of ER-positive tumor and Grade 2 tumor patients (**f**) and distant metastasis-free survival (DMFS) of ER-positive and LN-negative breast tumor patients (**g**). Panels c through g are from the TCGA breast cancer data.

Because age-associated changes in the expression of regulatory genes might be indicative of dysregulated pathways underlying the biology of BC and its four intrinsic subtypes in young women, we reasoned that the delineation of molecules that are selectively altered in BCYW ≤ 40 years old might provide clues regarding the genomic basis of the aggressive nature of BCYW. To explore this hypothesis, we curated The Cancer Genome Atlas (TCGA) dataset for Breast Invasive Carcinoma (Firehose Legacy Study) from the cBioPortal for Cancer Genomics and analyzed the genomic alterations of the following three age groups: ≤40, 41–54, and ≥55 years. While keeping a minimum cut-off of log 2-fold change over built-in internal adjacent normal specimens, we identified genes that were upregulated and downregulated in each age group either uniquely or commonly between two or three age groups. For example, we observed the upregulation of 60 genes (out of 558 upregulated genes with a cut-off of log 2-fold change) and downregulation of 58 genes (out of 527 downregulated genes with a cut-off of log 2-fold change) uniquely in patients with BC aged ≤40 years (Figure 1a and Supplementary Figures S1 and S2). Additionally, 42, 51, and 405 genes were upregulated, respectively, in the age groups of ≤40 and 41–54 years; age groups of ≤40 and ≥55 years; and all three age groups. Results from the Gene Ontology biological function analysis of 45 mapped genes out of 60 upregulated genes in breast tumors from patients aged <40 years had diverse functions (Supplementary Table S2). As expected, shared genes among the three age groups represented the largest set of dysregulated genes.

To broaden the relevance of these findings to BCYW, we also analyzed the curated datasets from the Molecular Taxonomy of Breast Cancer International Consortium (METABRIC)—a United Kingdom- and Canada-based initiative [39]. Similar to our observation with the TCGA dataset, we identified 109 upregulated (out of 300 upregulated genes) and 67 downregulated (out of 421 downregulated genes) genes unique to BC in women aged ≤40 years with a cut-off of log 1-fold change (Figure 1b and Supplementary Figure S3). However, there was no significant overlap among upregulated genes in patients aged ≤40 years between the two datasets, except for three genes (i.e., LAMP3, HAMP, and CLIC3) as well as for two other examples of differentially expressed gene sets identified for BC <45 vs. >55 years [26] and <45 vs. >65 years [27] (Supplementary Figure S4). Because the TCGA Firehose Legacy and METABRIC datasets were constructed using distinct patient pools and with different sequencing and gene expression platforms, a lack of overlap between the two analyses is understandable. Nevertheless, it underscores the critical point regarding the existence of uniquely dysregulated genes in BCYW aged ≤40 years from different parts of the world—highlighting the potential importance of geographical disparities. In subsequent analyses, we used the TCGA Firehose Legacy data for examining the nature of dysregulated genes in BCYW.

Figure 1c illustrates the presence of upregulated or downregulated genes whose expression increased or decreased from groups aged ≤40 years to 41–54 years and to >55 years, highlighting the shared components of age-independent dysregulated genes in BC. Next, we assessed the significance of 60 upregulated genes in BCYW by determining the correlation of gene expression with the overall survival (OS), relapse-free survival (RFS), or distant metastasis-free survival (DMFS) of patients with BC, with a mean follow-up of 10 years, in 1881 tumor samples representing 19 cancer subtypes plus untreated and tamoxifen-treated groups by using the Gene Expression–Based Outcome for the Breast Cancer Online (GOBO) database platform [42]. Among the 60 upregulated genes, 38 were mapped in the database and thus used in subsequent analyses. The expression of high and low levels of these 38 genes in BC was not significantly different according to the multivariant Kaplan–Meier analysis for BC subtypes (Supplementary Figure S5a), expect in the ER-positive lymph-node (LN)-negative subtype (Figure 1d, *p*-value = 0.00204, *n* = 380 patients). The expression status of 60 upregulated genes in the cBioPortal Genomic platform indicated a substantial upregulation (8–19%) of a subset of 17 genes in BC (Figure 1e, Supplementary Table S3). Analysis of the clinical relevance of 11 of 17 highly upregulated genes (as 6 genes were not mapped in the database) revealed that the overexpression of these 11 genes significantly affected RFS (*p*-value = 0.00828, *n* = 738 patients) and the DMFS (*p*-value = 0.00221, *n* = 856 patients) among patients with ER-positive tumors (Figure 1f,g; Supplementary Figure S5d) in the absence of any other factor influencing the OS of young women with BC (Supplementary Figure S5e). However, the overexpression of these 11 genes had opposite effects on RFS for the basal versus ER-positive breast tumors and on DMFS for ERBB2/HER2-positive versus ER-positive LN-negative tumors in young women with BC (Supplementary Figure S5e). Thus, the subset of 11 out of 60 upregulated genes in BCYW aged ≤40 years might be associated with differential outcomes for distinct subtypes, such as a poor outcome for ER-positive tumors and a good outcome for HER2-positive tumors.

Though preceding observations were made using the candidate mRNAs, it implied a corresponding change in the levels of gene products/proteins. To support this notion within the constraints of the limited availability of the mRNA and protein data from the same set of breast tumor specimens in public databases, we examined the status of the mRNA–protein correlation of 60 genes uniquely upregulated in breast tumors from patients aged ≤40 years. After removing the genes with no expression of mRNA or protein, we presented examples of genes with data at the levels of both mRNA and protein. Results presented in the Supplementary Figure S6 suggested a similar trend of mRNA and protein expression of 15 genes. As many of the candidate 60 genes were also detected as proteins in secreted extracellular vesicles as well as in the human plasma database (see below), these observations strengthen the idea of the upregulation of the corresponding proteins, at least in part. However, these observations do not rule out the possibility of additional post-translational modifications of these 60 upregulated genes and resulting changes in respective proteins—an area of research not pursued in the present study.

### 3.2. Subset of Differentially Dysregulated and Clinically Significant Genes in BCYW

**Figure 2:**
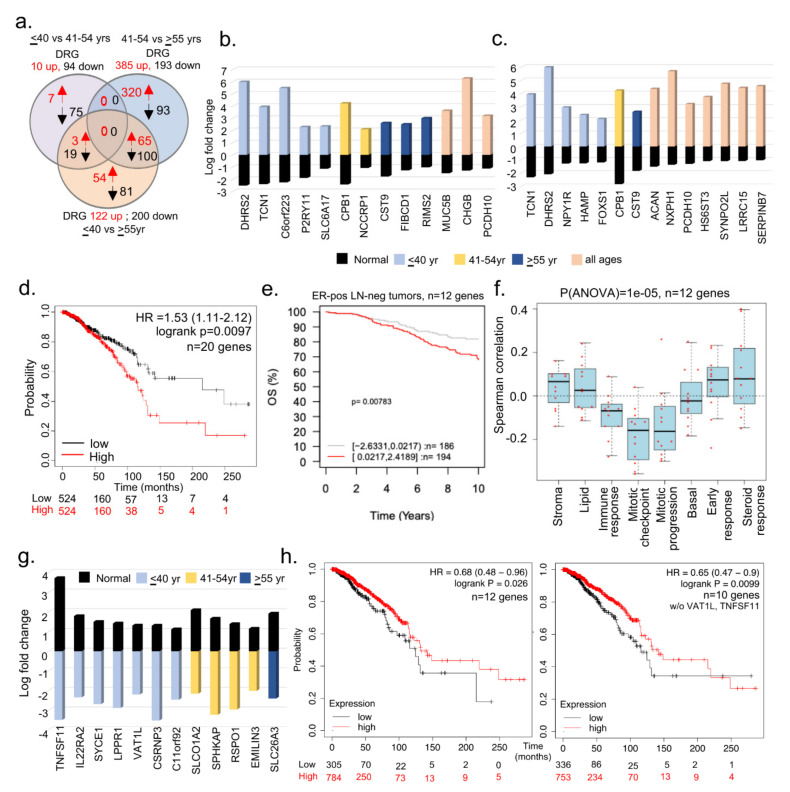
Comparative analysis of BCYW genes in matching normal and tumor specimens. (**a**) Overlap of dysregulated genes among ≤40 vs. 41–54 years, ≤40 vs. ≥55 years, and 41–54 vs. ≥ 55 years age groups of adjacent matching normal breast tissue samples. Red, upregulated, and blue, downregulated genes; (**b**,**c**) bar chart showing the levels of indicated shared DRG genes in breast tumors from groups of ≤40 versus 41–54 years (**b**), ≤40 versus >55 years (**c**), or shared among three age groups, as well as in corresponding adjacent normal breast tissues; (**d**) multivariant analysis of 20 genes (DHRS2, TCN1, SLC6A17, CPB1, NCCRP1, CST9, FIBCD1, RIMS2, CHGB, NPY1R, SYNPO2L, ACAN, NXPH1, SERPINB7, HAMP, FOXS1, HS6ST3, MUC5B, LRRC15, and P2RY11) for overall survival of breast cancer patients using the using the online KMPlot tools; (**e**) overall survival (OS) of ER-positive (ER-pos) lymph node negative (LN-neg) breast tumors stratified on the basis of high (red) and low (gray) expression of the 12 genes (TNFSF11, IL22RA2, SYCE1, PLPPR1, VAT1L, CSRNP3, COLCA1, SLCO1A2, SPHKAP, RSPO1, EMILIN3, and SLC26A3) out of 22 upregulated genes in panels b and c; (**f**) Spearman correlation analysis of 12 genes in the context of 8 preset co-expression gene modules; (**g**) levels of 12 genes upregulated in adjacent normal breast samples but downregulated in tumor samples; and (**h**) overall survival analysis of 12 (left) or 10 (right panel, without VAT1L and TNFSF11) genes described in panel using the online KMPlot tools.

The analysis of breast cancer TCGA data provided the status of differentially expressed genes (DEGs) in cancer tissues compared with gene levels in adjacent normal matching tissues. Because certain genes might be common among the adjacent normal tissues of different age groups, we next identified DRGs within adjacent normal tissues between the specimens from the ≤40 vs. 41–54 years or ≤40 vs. >55 years age groups, and then used these DRGs to compare with the significantly altered genes in BC tissues among different age groups. We found 10 upregulated and 94 downregulated DEGs in matching adjacent normal tissues of the ≤40 versus 41–54 years age groups, whereas 122 upregulated and 200 downregulated DEGs were found in matching normal tissues of the ≤40 versus >55 years age groups (Figure 2a). Furthermore, we determined unique or shared DEGs in adjacent normal tissues between the age groups of ≤40 versus 41–54 years (Figure 2b) and ≤40 versus >55 years (Figure 2c) and in breast tumors from all three age groups. The gene ontology analysis of biological functions suggested that 19 of these 22 genes might be potentially involved in growth factor signaling and metabolic transport (Supplementary Table S4). Collectively, this analysis revealed that 22 genes were downregulated in matching normal tissues in patients aged ≤40 years but were upregulated in breast tumors of patients from all three age groups (Figure 2b,c). Figure 2d demonstrates that the overexpression of 20 (excluding PCDH10 and C6orf223 genes) of 22 genes (named in Figure 2b,c) in breast tumors is associated with a significant reduction in survival compared with a low expression of these genes (*p*-value = 0.0097). In addition, we also analyzed these 20 upregulated genes in the GOBO database. However, only 12 out of these 20 genes were mapped in the GOBO dataset and were, therefore, used in further analyses (Figure 2e,f; Supplementary Figure S7a,b). The overexpression of these 12 genes in breast tumors was significantly correlated with the reduced OS of patients with the ER-positive LN-negative (Figure 2e; *p*-value = 0.00783, *n* = 380 cases) and PAM50 normal-like (*p*-value = 0.00664, *n* = 124 cases) subtypes (Supplementary Figure S7b). The association between these 12 upregulated genes and PAM50 normal-like tumors was revealing; however, it provides no clues regarding the relevance of the observed upregulation of cancer-associated genes in normal breast tissues. It is possible that these genes are involved in events beyond the normal physiology in normal breast tissues, such as in disease-associated processes which have yet to be clinically manifested. The overexpression of these 12 genes correlated well with the improved OS of patients with the LN-positive subtype (*p*-value = 0.00169, *n* = 215 cases; Supplementary Figure S7b). No significant effect of the 12 upregulated genes on the RFS of patients was observed, except in patients with the LN-positive subtype. Furthermore, the relevance of these 12 upregulated genes in breast tumors of women aged ≤40 years was evident in a high Spearman correlation with 8 gene set modules [43] pertaining to stroma, lipid, early response, and steroid responses (Figure 2f).

In addition to the 20 upregulated genes in breast tumors of women aged ≤40 years, we identified 12 genes that were upregulated in adjacent normal tissues but downregulated in breast tumors in three age groups (Figure 2g, Supplementary Table S5). We predicted that the loss or reduction of these genes might be involved in cancer-promoting processes. However, this needs to be experimentally demonstrated in near future studies, perhaps using a combinatorial gene strategy in an appropriate whole animal model. As expected, the downregulation of these 12 genes or 10 genes (excluding TNFSF11 and VAT1L genes from the list in Figure 2g) was associated with the reduced survival of patients with BC compared with that of patients with an upregulation of these genes (Figure 2h). Contrary to previous studies [50], the expression of TNFSF11/RANKL was consistently downregulated in patients aged ≤40 years with breast tumors as compared with adjacent normal breast tissues.

**Figure 3:**
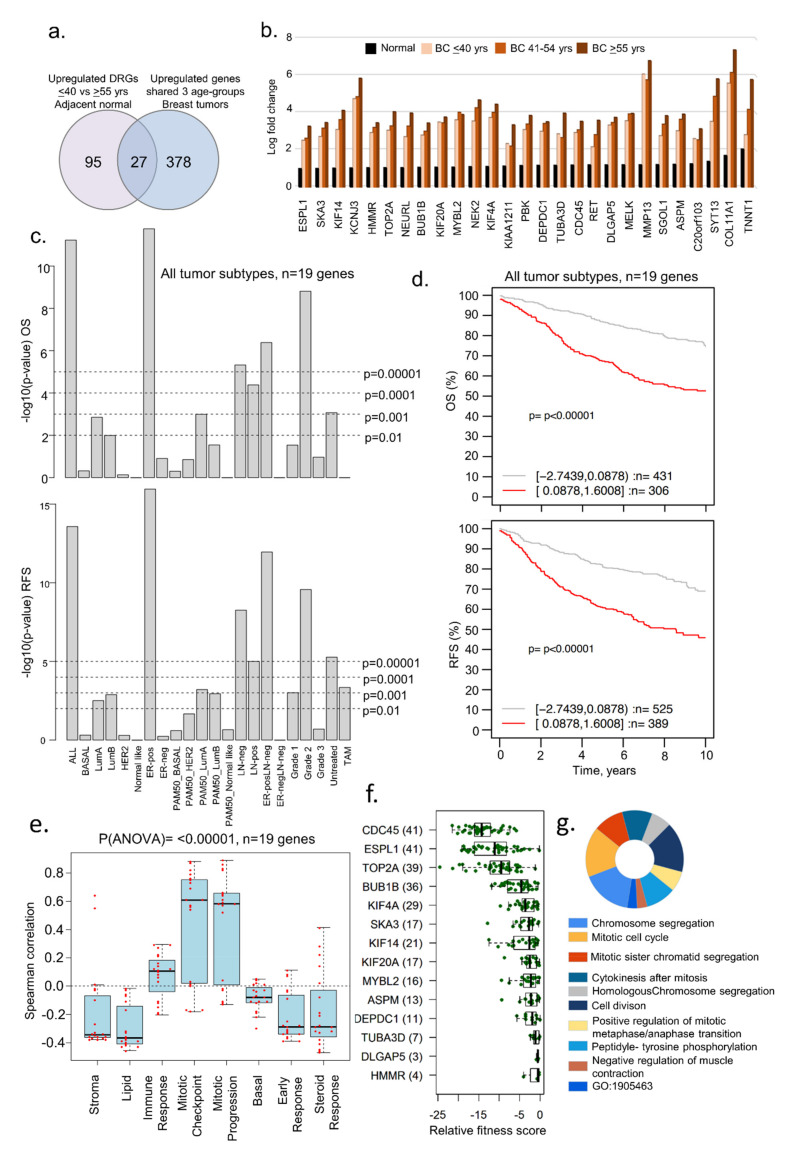
Examples of genes upregulated in adjacent normal and tumor samples. (**a**) Venn diagram representing the overlap of upregulated genes in all the age groups of tumor samples as well as in ≤40 vs. ≥55 years adjacent normal tissues; (**b**) bar chart representing levels of 27 upregulated genes identified from the overlap analysis in panel a; (**c**) Kaplan–Meier OS and RFS summaries of 19 of 27 genes mapped in the GOBO database with 1881 breast tumors; (**d**) OS and RFS curves using the high (red) and low (gray) expression of 19 genes in all tumor subtypes; (**e**) Spearman correlation analysis of 19 genes in the context of co-expressed 8 gene modules; (**f**) boxplot representing the fitness score of indicated genes in breast cancer cell lines; and (**g**) GO biological functional analysis of 26 of the 27 genes, except HMMR. All survival and correlation analyses were performed using the online GOBO tools.

Next, we determined whether 122 differentially upregulated genes in the adjacent normal tissues from the age groups of ≤40 years versus ≥55 years also shared common genes in the three age groups of patients with breast tumors. We found that 27 (out of 122) DEGs were upregulated in adjacent normal breast tissues and in breast tumors from the three age groups (Figure 3a,b, Supplementary Table S6). Of these 27 genes, 19 were mapped in the GOBO database and used for evaluating the clinical significance of these genes. The overexpression of these shared 19 upregulated genes was associated with a substantially shorter OS and RFS in several breast tumor subtypes (Figure 3c) as well as average OS and RFS in all tumors (Figure 3d; OS *p*-value = 0.0001, *n* = 737; RFS *p*-value = 0.0001, *n* = 914). Consistent with this observation, the overexpression of these 19 genes correlated positively with gene sets belonging to immune and mitosis processes (Figure 3e). It is possible that such genes might be primarily involved in the cellular proliferation of breast tumors. In line with this observation, we found that 14 of 19 genes were also fitness genes (Figure 3f) with diverse functions (Figure 3g), that is, the individual depletion of these genes in multiple BC cell lines led to the loss of cell viability in a CRISPER-9 database [45]. In brief, these results suggested that several uniquely upregulated genes in breast tumors of women aged ≤40 years were also upregulated in adjacent normal breast tissues. Because many of these genes have known roles in cancer progression, the biological significance of cancer-relevant upregulated genes in normal breast tissues remains unclear.

### 3.3. Evidence of Dysregulated Cancer Genes in BCYW and Normal Breast Tissues

**Figure 4:**
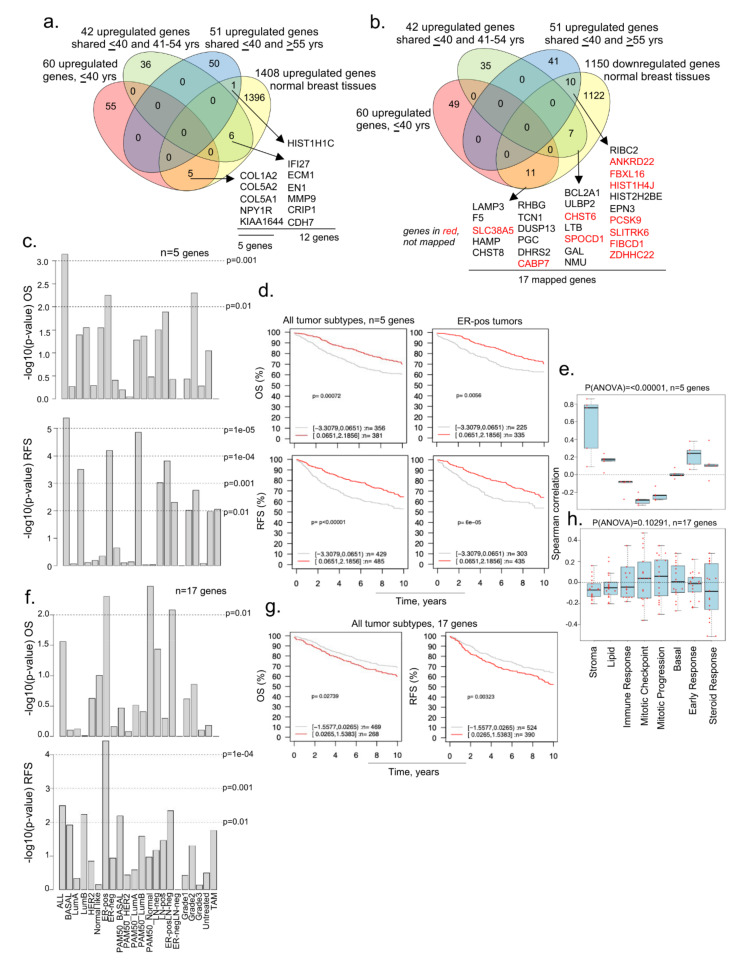
Comparative analysis of upregulated genes in BCYW aged ≤40 years with modulated genes in normal breast tissues. (**a**,**b**) Overlap of upregulated genes in ≤40 years age group and/or shared upregulated genes between the ≤40 years and 41–54 years or ≥55 years age groups with upregulated (**a**) or downregulated (**b**) genes in normal breast tissues; (**c**) Kaplan–Meier OS and RFS summaries of 5 genes mapped in the GOBO database with 1881 breast tumors; (**d**) OS and RFS curves using the high (red) and low (gray) expression of 5 genes in all tumor subtypes and ER-positive (ER-pos) breast tumors; (**e**) Spearman correlation analysis of 5 genes in the context of co-expressed 8 gene modules; (**f**) Kaplan–Meier OS and RFS summaries of 17 genes mapped in the GOBO database with 1881 breast tumors; (**g**) OS and RFS curves using the high (red) and low (gray) expression of 5 genes in all breast tumor subtypes; and (**h**) Spearman correlation analysis of 17 genes in the context of co-expressed 8 gene modules.

As adjacent normal tissues have been shown to not always mirror normal breast tissues, and because bi-directional cancer–stroma interactions and the resulting secreted molecules are likely to influence the genomic alterations in adjacent normal tissue [51,52], we examined the status of dysregulated genes in breast tumors from patients aged ≤40 years in normal breast tissues from healthy donors. To this effect, we used a recent RNA-sequencing dataset derived from the breast tissues of 151 parous women without a history of BC, listing 1408 upregulated and 1150 downregulated genes [31]. Although this study did not consider three age groups, it still served as a large gene set modulated genes for comparative analysis with breast cancer dysregulated genes. Next, we compared 1408 upregulated and 1150 downregulated genes in normal breast tissues [31] against dysregulated genes in BCYW ≤40 years and shared genes between the age groups of ≤40 years and 41–54 years or >55 years (Figure 4a,b). Among the shared upregulated genes in normal breast tissues and breast tumors, the expression of five genes unique to young women aged ≤40 years with BC (i.e., COL1A2, COL5A2, COL5A1, NPY1R, and KIAA1644) significantly affected the OS and RFS of patients with several tumor subtypes (Figure 4a). For example, the high expression levels of the five genes correlated well with the OS (*p*-value = 0.00072, *n* = 737) and RFS (*p*-value = 0.00001, *n* = 914) for patients with several subtypes of BC as compared to those with the low expression of these genes (Figure 4c,d). As collagen genes are considered crucial for the stromal microenvironment and extracellular matrix remodeling [53,54], while hypoxia inducible NPY1R [55] has been shown to be a determinant of breast cancer responsiveness to hormonal sensitivity and a secreted predictive marker for breast cancer metastasis [56,57], the increased expression of these molecules might be associated with a poor prognosis. We also noticed a significant positive Spearman correlation of these five genes with the gene sets related to stroma, lipid, early response, and steroid response (*p*(ANOVA) ≤ 0.00001; Figure 4e).

We also observed that 28 overexpressed genes in BCYW aged ≤40 years shared with those aged 41–54 and >55 years were downregulated in normal breasts (Figure 4b). Of these 28 genes, 17 could be mapped in the GOBO database and hence were used in subsequent analyses (Figure 4f–h). The results indicated that the overexpression of these 17 shared genes correlates well with the OS for ER-positive PAM50 normal-like and ER-positive LN-negative subtypes (Figure 4f, *p*-value > 0.01) and the RFS for all tumor subtypes (Figure 4g,h; *p*-value = 0.00323, *n* = 914).

**Figure 5:**
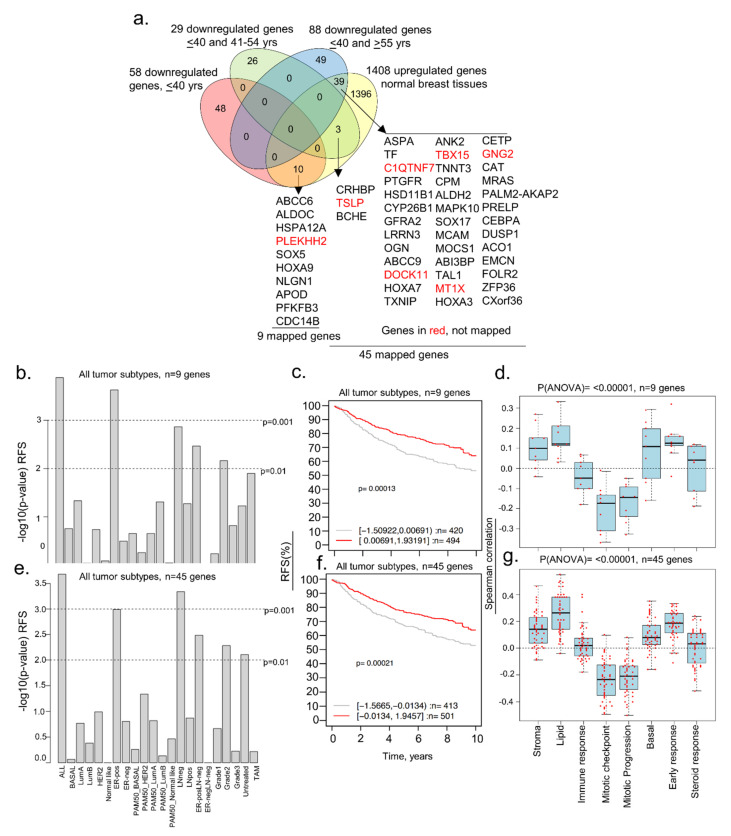
Comparative analysis of downregulated genes in BCYW aged ≤40 years with modulated genes in normal breast tissues. (**a**) Overlap of downregulated genes in ≤40 years age group and/or shared downregulated genes between the ≤40 years and 41–54 years or ≥ 55 years age groups with upregulated genes in normal breast tissues; (**b**) Kaplan–Meier RFS summaries of 9 genes mapped in the GOBO database with 1881 breast tumors; (**c**) RFS curve using the high (red) and low (gray) expression of 9 genes in all breast tumor subtypes; (**d**) Spearman correlation analysis of 9 genes in the context of co-expressed 8 gene modules; (**e**) Kaplan–Meier RFS summaries of 45 genes mapped in the GOBO database with 1881 breast tumors; (**f**) RFS curves using the high (red) and low (gray) expression of 45 genes in all breast tumor subtypes; and (**g**) Spearman correlation analysis of 45 genes in the context of co-expressed 8 gene modules. All survival and correlation analyses were performed using the online GOBO tools.

We explored the possibility that a subset of genes downregulated in women aged ≤40 years with BC might be upregulated in normal breast tissues. To this effect, we found that 52 upregulated genes in normal breast tissues were downregulated in BC tissues of women aged ≤40 years, of which 10 were unique to breast tumors of women aged ≤40 years, and 42 were shared between the age groups of ≤40 and 41–54 years or >55 years (Figure 5a). Nine of the ten downregulated genes in breast tumors of patients aged ≤40 years were mapped in the GOBO database. High and low expression levels of these nine genes correlated well with the RFS (but not OS) in several breast tumor subtypes (Figure 5b) and all tumors (Figure 5c; *p*-value = 0.00013, *n* = 914), revealing the potential role of these nine genes in the RFS of breast cancer patients. These findings were also true for 45 downregulated genes, inclusive of 9 genes (out of 52 genes as 7 genes could not be mapped in the GOBO database) in BCYW ≤40 years, which were upregulated in normal breast tissues (Figure 5e,f). Both the 9 and 45 gene sets were positively correlated with gene modules related to stroma, lipid, basal, early response, and steroid response, but negatively related to mitotic gene modules (Figure 5d,g; *p*(ANOVA) ≤ 0.00001).

Because BCYW has been linked to menstrual cycle-associated hormonal changes [58,59], we searched for a potential overlap of dysregulated genes in breast tumors of women aged ≤40 years with 255 DEGs in the luteal phase as compared with the follicular phase of the menstrual cycle in young healthy women (≤40 years, *n* = 17; 41–46 years, *n* = 3) [34]. We found that 3 (PTPRN, HIST1H2BH, and HIST1H2AM mRNAs) of 60 upregulated genes in breast tumors of patients ≤40 years were also upregulated in normal breast tissues in the luteal phase (Supplementary Figure S8a). In this context, PTPRN is a hypoxia-inducible phosphatase with a role in BC angiogenesis [60], while the expression of HIST1H2BH and HIST1H2AM is upregulated in human tumors with a suggested prognostic role [61,62]. Because the observed upregulation of replication-dependent histones HIST1H2BH and HIST1H2AM as the component of gene expression machinery and the process of hypoxia are fundamental processes in normal tissues, the stimulation of molecules by yet-to-be defined triggers in normal tissues might set the stage for early precancerous processes over a period. In addition, the expression of TNFSF11/RANKL—which was found to be downregulated in breast tumors of patients aged ≤40 years—was upregulated in normal breast tissues (Supplementary Figure S8b), similar to the upregulation in another dataset of normal breast tissues (Figure 2g).

As in the TCGA dataset breast tumors, we also noticed that 8 out of 109 upregulated genes specific to breast tumors from patients aged ≤40 years in the METABRIC dataset were also common to upregulated genes in normal breast tissues, while 17 were downregulated in normal breast tissue (Supplementary Figure S8). High and low expression levels of these 16 (of 17) genes correlated well with the OS and RFS for several breast tumor subtypes (Supplementary Figure S9c). Similarly, 124 of the upregulated genes in normal breasts during the luteal phase of the menstrual cycle were also upregulated in METABRIC breast tumors from patients aged ≤40 years (Supplementary Figure S9b).

### 3.4. Dysregulated Genes in BCYW Vary with the Intrinsic Tumor Subtype

**Figure 6:**
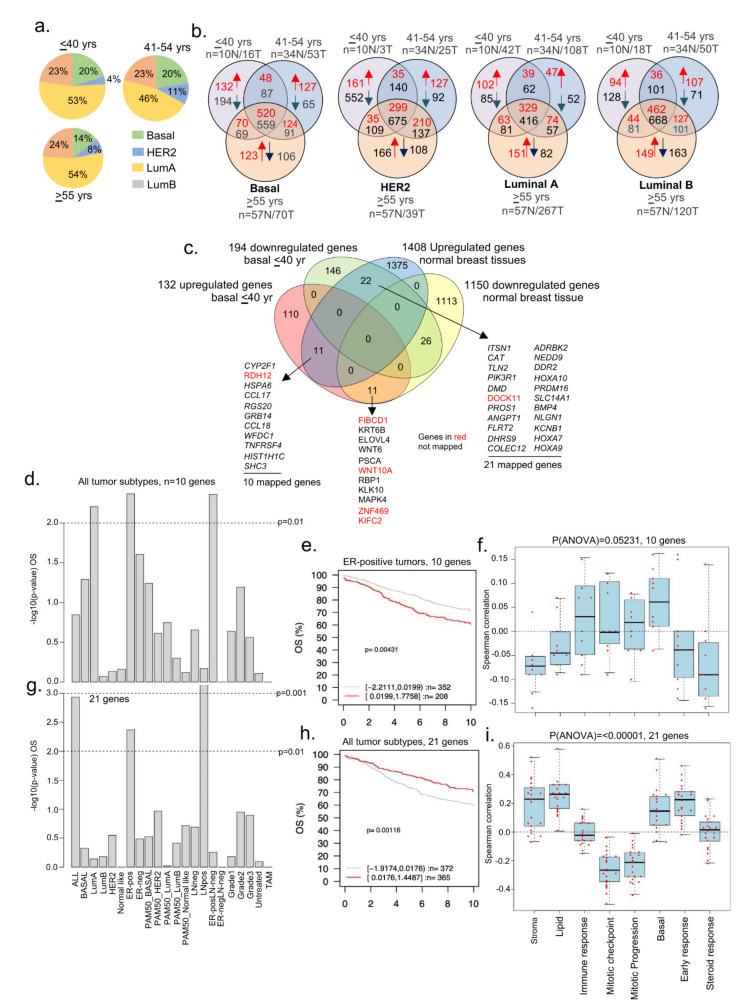
Status of BCYW genes in intrinsic breast tumor subtypes. (**a**) Pie chart showing distribution of four intrinsic breast tumor subtypes in 3 age groups; (**b**) overlap of dysregulated DRG genes among ≤40 vs. 41–54 vs. >55 years age groups. Red arrow, upregulated, and blue arrow, downregulated genes; (**c**) overlap of up- and downregulated genes in basal breast tumors from patients aged ≤40 years with upregulated or downregulated genes in normal breast tissues; (**d**) Kaplan–Meier OS summaries of 10 genes, upregulated in basal breast tumors and normal breast tissues, mapped in the GOBO database with 1881 breast tumors; (**e**) OS curve using the high (red) and low (gray) expression of 10 genes in all breast tumor subtypes; (**f**) Spearman correlation analysis of 10 genes in the context of co-expressed 8 gene modules; (**g**) Kaplan–Meier OS summaries of 21 mapped genes mapped in the GOBO database with 1881 breast tumors; (**h**) OS curves using the high (red) and low (gray) expression of 45 genes in all breast tumor subtypes; and (**i**) Spearman correlation analysis of 21 genes in the context of co-expressed 8 gene modules. All survival and correlation analyses were performed using the online GOBO tools.

Based on the status of ER-alpha, PR, and HER2, BC can be classified into four intrinsic subtypes, each with characteristic clinical features. Studies have shown that BCYW is generally more aggressive with a poorer prognosis than BC in older patients [16,17,18]. A high prevalence of TNBC, basal-like tumors, and luminal B exists in BCYW [10,11,12,13,14,15,16,17]. However, the effect of cancer subtypes on the genome of women with BC aged ≤40 years remains poorly studied. While keeping a minimum log 2-fold change as a cut-off, we determined the expression of dysregulated genes in four intrinsic breast subtypes as a function of distinct age groups. The results indicated that the ratios of BC subtypes did not differ significantly between the three age groups (Figure 6a). This observation is not in line with previous data that showed a high prevalence of TNBC/basal-like cancer in BCYW [12], presumably due to the use of a minimum log 2-fold change in computing our findings in the present study. Figure 6b demonstrates the numbers of upregulated and downregulated genes in each of four intrinsic breast tumor subtypes and gene overlaps among the three age groups.

In line with the goal of the present study regarding the analysis of BCYW, we focused on dysregulated genes unique to breast tumors of women aged ≤40 years and assessed their potential overlap with 1408 upregulated and 1150 downregulated genes in normal breast tissues [31]. For example, 11 upregulated genes were common in the basal tumor subtype of patients aged ≤40 years and normal breast tissues, whereas 22 genes upregulated in normal breast tissues were downregulated in the basal tumor subtype (Figure 6c). Ten of eleven mapped genes upregulated in basal tumors from patients aged ≤40 years were divided into high- and low-expressing groups in OS analysis. A high expression status of these genes correlated well with the reduced OS of BC patients as compared with that of low-expression groups for three tumor subtypes: luminal A, ER-positive LN-negative, and ER-positive tumors (Figure 6d; *p*-value = 0.00431, *n* = 560 for ER-pos tumors). The expression of these nine genes exhibited a positive Spearman correlation with immune response and basal gene modules (Figure 6f), consistent with the basal subtype phenotype of the tumors analyzed. However, we observed a negative correlation with gene modules associated with stroma, lipid, early response, and steroid response.

We analyzed 21 mapped genes out of the 22 shared genes upregulated in normal breast tissues and downregulated in breast tumors of women aged ≤40 years. We found that the difference between the high- and low-expression tumors significantly affected OS for all tumor subtypes, including ER-positive and LN-positive subtypes (Figure 6g,h). However, as opposed to the 10 genes upregulated in both breast tumors and normal breast tissues (Figure 6d,e), a low expression of 21 genes was associated with the reduced OS of BC patients compared with the high expression of those 21 genes for all tumor subtypes (Figure 6g) as well as for ER-positive (*p*-value = 0.00424, *n* = 560) and LN-positive (*p*-value = 0.00056, *n* = 215) subtypes. The correlation of these 21 genes with all eight functional gene modules was opposite to that of the 10 genes upregulated both in breast tumors of patients aged ≤40 years and normal breast tissues (Figure 6i). This might be associated with the opposite expression of these genes—the upregulation of 10 genes and downregulation of 21 genes—in patients aged ≤40 years with breast tumors, providing clues regarding the role of some of these downregulated genes in stromal functions in BCYW.

We also performed the above analysis for HER2, luminal A, and luminal B subtypes. In the case of HER2-positive breast tumors, 52 downregulated genes in the breast tumors of women aged ≤40 years were found to be upregulated in normal breast tissues (Supplementary Figure S10a). Of the 52 genes, 44 were mapped in the GOBO database. The difference in high and low expression levels of these 44 genes was significantly correlated with OS for only LN-positive tumors (Supplementary Figure S10b,c; *p*-value = 0.000229, *n* = 215). However, the levels of expression of these 44 genes significantly correlated with the RFS of ER-positive, LN-negative, and LN-positive and grade 2 tumors (Supplementary Figure S10d) as well as the mean of all tumor subtypes (Supplementary Figure S10e, *p*-value = 1 × 10^−5^). As in HER2-positive tumors, the expression of 44 genes is associated with stroma and lipid gene modules but not with mitotic progression (Supplementary Figure S10f). Contrary to basal and HER2-positive tumors, no significant correlation was observed between OS and the expression levels of 8 genes that were upregulated in normal breast tissues but downregulated in luminal A tumors (Supplementary Figure S11a,b). However, 11 of the mapped genes out of the 14 genes downregulated in normal breast tissues and upregulated in luminal A tumors were significantly correlated only with ER-positive LN-negative tumors (Supplementary Figure S11a). Likewise, the expression levels of 7 genes upregulated in luminal B but downregulated in normal breast tissues correlated well with the OS and RFS for several tumor subtypes as well as for the mean of all the tumor subtypes (Supplementary Figure S12b). In brief, these results revealed that several genes that significantly affect OS, RFS, or both and are uniquely expressed in breast tumors in women aged ≤40 years were common to normal tissues and that such gene overlaps were tumor subtype specific.

### 3.5. Tumor Laterality and Age-Associated Dysregulated Genes 

**Figure 7:**
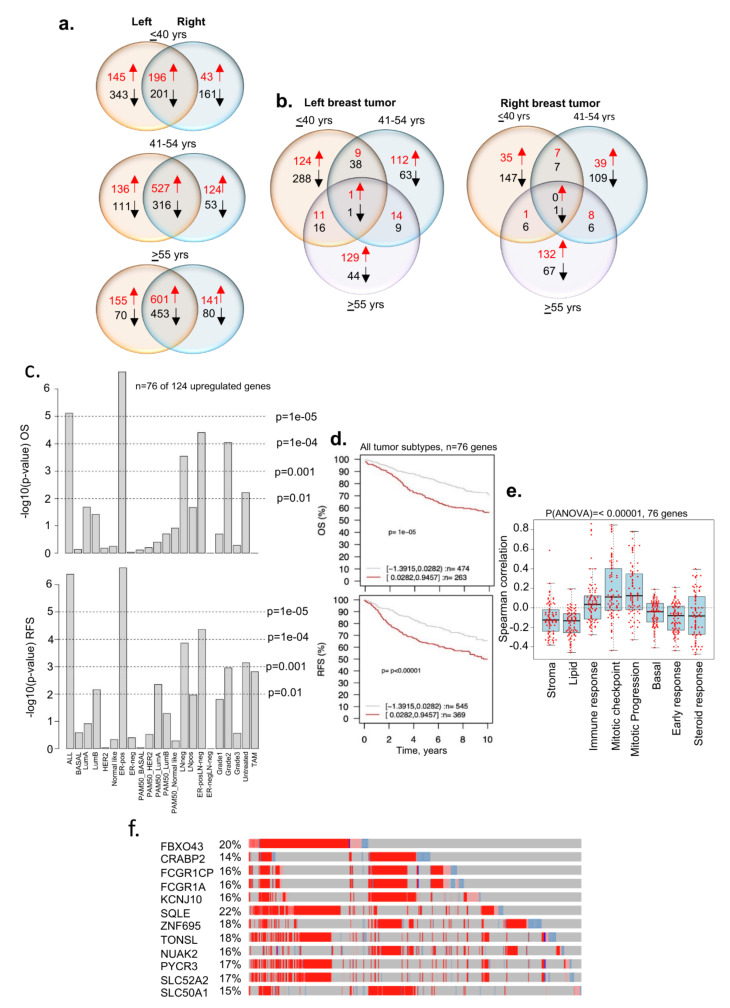
Breast cancer tumor genes from patients aged ≤40 years and tumor laterality. (**a**) Status of dysregulated DRG genes in the left and right breast tumors from three age groups; (**b**) overlap of dysregulated DRG genes within left tumors or right tumors from three age groups; (**c**) Kaplan–Meier OS and RFS summaries of 76 of 124 genes upregulated in the left breast tumors from patients aged ≤40 years; (**d**) OS and RFS curves using the high (red) and low (gray) expression of 76 genes in all breast tumor subtypes; (**e**) Spearman correlation analysis of 76 genes in the context of co-expressed 8 gene modules; and (**f**) expression of shortlisted 12 highly overexpressed genes. The percentage adjacent to the gene symbol represents the alteration rate. The data were curated from the cBioPortal platform. All survival and correlation analyses were performed using the online GOBO tools.

Numerous studies have suggested a higher percentage of BC in the left breast than in the right breast [63,64,65], including in the cases of bilateral BC [66]. Furthermore, studies have shown a poor OS of patients with tumors in the left breast and that such tumors were highly angiogenic in nature [67]. Moreover, certain aspects of BC laterality have been validated in a transgenic EERB2 murine model [68], showing differences in gene expression in both control and transgenic tumor-bearing mice. Recent studies have shown differences in DNA methylation and gene expression patterns between the left and right breast tumors [69] and a greater involvement of estrogen receptor signaling in the right breast tumors than in left breast tumors [70]. As the underlying basis of BC laterality in the context of age and BCYW remains unknown, we examined the prevalence of age-associated transcriptomic changes in breast tumors. Figure 7a demonstrates the existence of distinct as well as shared gene expression between the left and right breast tumors for all three age groups. For example, 124 and 35 genes were upregulated in the left and right breast tumors, respectively, in women aged ≤40 years (Figure 7b, Supplementary Table S7 for upregulated genes). Of 124 upregulated genes in the left breast tumors, 76 were mapped in the GOBO database. We found that the high expression of these 76 upregulated genes in left breast tumors correlates well with the significantly poor OS and RFS of patients with all subtypes (OS, *p*-value = 1 × 10^−5^, *n* = 737; RFS, *p*-value ≤ 0.00001, *n* = 914), including for ER-positive, LN-negative, and ER-positive LN-negative subtypes (Figure 7c,d). Furthermore, increased gene expression positively correlated with cell cycle mitotic progression gene sets (Figure 7e). Interestingly, 12 of 76 mapped genes are highly upregulated in breast tumors in the cBioportal dataset (Figure 7f) and 11 of these genes have been shown to be involved in metabolism (Supplementary Table S8). In contrast to left tumors, the high or low expression of 18 of 35 upregulated genes in the right breast tumors did not show any significant differences in OS (Supplementary Figure S13). Similarly, upregulation and downregulation of distinct sets of DEGs were observed between the left and the right adjacent normal breast tissues from the TCGA breast cancer Firehose dataset, as expected (Supplementary Figure S14). As these results were derived from a small number of adjacent normal breast tissues, clearly these preliminary results need to be tested in a larger clinical trial in the future.

In brief, the results presented here suggest that a subset of genomic changes (and consequently, cellular pathways) of breast tumors in young women are likely to be laterally linked and associated with the left breast tumors. Because such changes are also reflected in the adjacent normal breast tissues, laterality-associated inter-connected genomic changes are possible under the influence of hormonal and metabolic alterations, and such changes might be further increased in breast tumors.

### 3.6. Secretory Nature of Dysregulated Gene Products in Young Women’s Breast Tumors

BCYW shows the dysregulation of age-associated cancer-relevant genes in a subtype-specific manner; many of these genes are also dysregulated in adjacent matched normal or normal breast tissues. Uniquely dysregulated molecules in BCYW are likely crucial potential predictive or prognostic markers for BCYW and probably for BC at large. Because the results presented were derived from the analysis of breast tissues, the determination of whether some of these molecules are also secretory in nature and found in extracellular microvesicles/exosomes secreted from BC cell lines and in body fluids, such as plasma, urine, or saliva, is crucial. Therefore, we determined the presence of distinct subsets of dysregulated genes in secretome and plasma proteome databases.

**Figure 8:**
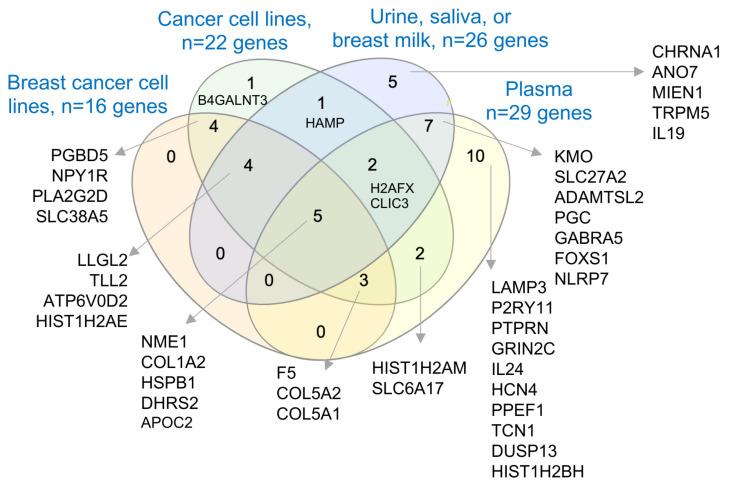
Secretory nature of upregulated genes in BCYW aged ≤40 years. Overlap of secreted proteins from breast cancer cell lines, cancer cell lines, body fluids such as urine, saliva, and breast milk, or plasma.

Previous studies have suggested that many cancer-relevant upregulated genes in breast cancer are also components of the cargos, packed in the extracellular microvesicles (EVs or exosomes) secreted by cancer cells [71], and thus are detectable either as mRNAs, proteins, or both in the EVs database, and that such gene products could also be found in the human plasma proteome database [47,48,49]. Accordingly, many of the molecules associated with BCYW aged ≤40 years and/or upregulated genes shared with normal breast tissues were detectable as proteins in EVs secreted from breast and cancer cell lines as well as in human body fluids such as urine, saliva, breast milk, and plasma. For example, among 60 upregulated genes unique to those aged ≤40 years, 16 gene products were found to be present in EVs secreted by breast cancer cell lines, of which 7 proteins (i.e., PGBD5, NPY1R, PLA2G2D, SLC38A5, F5, COL5A2, and COL5A1) were also common to other cancer cell lines, 4 proteins (i.e., LLGL2, TLL2, APT6V0D2, and HIST1H2AE) were in urine, saliva, or breast milk, and 5 proteins (i.e., NME1, COL1A2, HSPB1, DHRS2, and APOC2) were in breast cancer cell lines and human body fluids such as urine, saliva, breast milk, and plasma (Figure 8). The results of various comparative analyses presented in the preceding sections (i.e., Figure 1d,e; Figure 2b,c; Figure 3; Figure 4a,b; Figure 6d and Figure 7c) are summarized in Supplementary Tables S9 and S10. Overall, we noted a significant overlap between the BCYW-relevant gene products secreted by breast cancer cell lines and human body fluids such as urine, saliva, breast milk, and plasma (Supplementary Figure S15).

As with these TCGA dataset-based results, we also noticed that several of the 44 upregulated genes specific to breast tumors from patients aged ≤40 years in the METABRIC dataset (Supplementary Figure S8) were also secreted as cargo in extracellular vesicles/exosomes by breast and cancer cell lines and components of body fluids such as urine, saliva, breast milk, and plasma (Supplementary Figure S16). Though these findings need to be validated in prospective, well-controlled clinical trials, it underscores the critical point that many of the dysregulated genes/proteins in BCYW might be secreted in body fluids and thus could be pursued as the next generation of predictive biomarkers for the development and/or progression of BCYW.

## 4. Discussion

**Figure 9:**
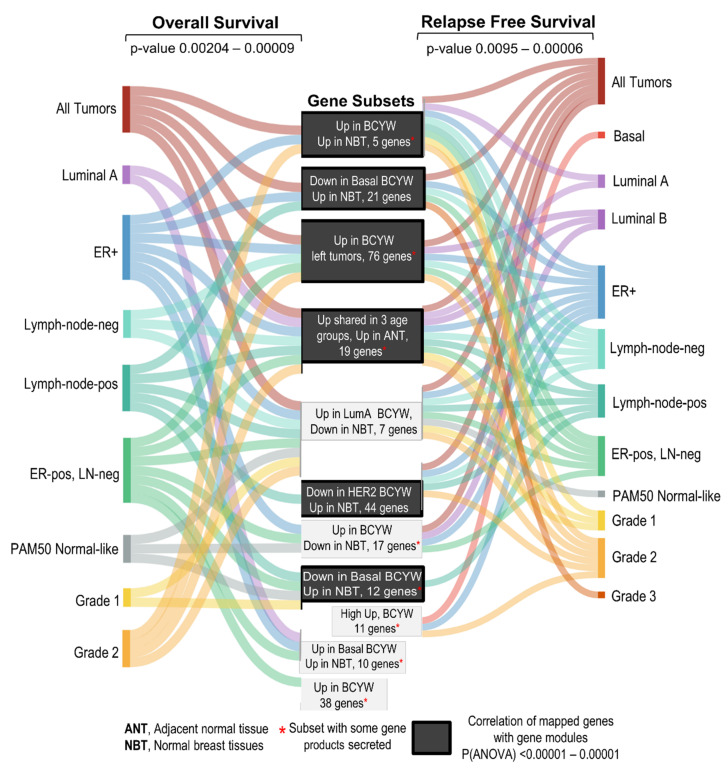
Summary of the observations discussed in the present study.

The present study delineated the nature of transcriptomic alterations in breast tumors in patients aged ≤40 years, four intrinsic BC subtypes, and their relationship with breast tumor laterality—all in comparison to breast tumors in the age groups of 41–54 or >55 years (see the summary of key observations in Supplementary Table S10 and Figure 9). We found that BCYW exhibits upregulation of 60 genes and downregulation of 58 genes, not shared with breast tumors from patients aged 41–54 or >55 years. Similar to these findings, using the TCGA breast tumor dataset, we also found the upregulation of 109 genes and downregulation of 67 genes in breast tumors from patients aged ≤40 years, but not in the other two age groups in the METABRIC breast tumor dataset. However, no significant overlap was observed among these unique gene sets between the two large datasets, except for LAMP3, HAMP, and CLIC3, which might be due to the use of different sequencing and gene expression platforms as well as differences in patient populations from different parts of the world. Nevertheless, these results reinforce the notion of the existence of unique and shared age-associated genomic and cellular insights of BCYW as well as the existence of geographical molecular disparities among BCYW from different parts of the world.

The overexpression of a subset of 60 upregulated genes was found to be clinically relevant, as evident through significant differences in the OS between the high- and low-expressing ER-positive LN-negative breast tumors. For example, 17 out of 60 genes were found to be highly overexpressed in BC, implying a wider significance of these genes beyond BCYW. We also recognized that the overexpression of 11 of these 17 genes resulted in significant differences in RFS or DMFS, but not in OS, for several breast tumor subtypes, such as basal and ER-positive. Interestingly, the overexpression of a gene set in tumor subtypes was not always associated with the reduced survival of patients with BC. For example, we found that the reduced expression of 11 genes was associated with a reduced RFS as compared to basal breast tumors with a high expression of these genes, whereas the reverse was true for ER-positive tumors, suggesting that dysregulated genes might exhibit tumor subtype-specific cellular and phenotypic effects. It remains unknown why certain gene sets might be more relevant for relapse-free survival but not overall survival. As the process of relapse is not only driven by the biology of the primary tumors but also by the ability of dormant tumor cells to reactivate and a small percentage of therapeutic-sensitive cells to acquire resistance over time, in addition to other cellular processes, the authors believe that the noted clinical differences are driven by the functions of the gene products as modulated by age-associated epigenomic and gene–environmental interactions. Unfortunately, due to the complex nature of such interactions and the inherent polygenic nature of breast cancer, it would be difficult to assign the noted clinical changes to specific genes as the field lacks valid model systems to test such hypotheses.

Our analysis also allowed us to identify genes that are downregulated in normal breast tissues but upregulated in breast tumors of women aged ≤40 years (as well as in the other two age groups), and that a high expression of such genes leads to the reduced survival of patients with BC. Conversely, 12 genes were upregulated in normal breasts but downregulated in BCYW aged ≤40 years and in the other two age groups. As expected, a low expression of these genes was correlated with the reduced survival of patients with BC compared with that of patients with a high expression of these genes. Similarly, 5 of 60 or 11 of 60 uniquely upregulated genes in breast tumors of patients aged ≤40 years were also upregulated or downregulated, respectively, in normal breast tissues from healthy donors. The expression levels of these genes correlated significantly with OS and RFS between the high- and low-expression groups. In addition, we also noticed examples of upregulated gene sets where there was a significant difference in the RFS or OS in the absence of OS or RFS, respectively, for a given tumor subtype among high- and low-expressing groups. As expected, this might be due to the potential involvement of a distinct set of pathways in regulating the processes that govern the survival versus relapse of breast tumors.

Another noticeable finding here is that both the nature of the uniquely dysregulated genes in breast tumors of patients aged ≤40 years and their overlap in normal breast tissues appear to be intrinsic and tumor subtype dependent. This suggests that the noticed phenotypic differences and worst prognosis of some BCYW cases might be explained, at least in part, by such subtype-specific genomic dysregulation. For example, the overexpression of 10 shared genes in basal breast tumors of patients aged ≤40 years as well as in normal breast tissues correlated with a substantial reduction in the OS of patients with BC, whereas the downregulation (but upregulation in normal breast tissues) of 21 genes in basal breast tumors of patients aged ≤40 years were associated with the reduced OS of patients with BC. As for the physiologic significance of these tumor-associated upregulated genes in the biology of normal breasts, the authors favor the hypothesis that many of these upregulated genes in the stromal or mammary epithelium might be an indication of dormant hyperactive pathways that could contribute to the development of BC later in life in response to an appropriate trigger or triggers. In addition, the findings presented here provide evidence to support the hypothesis that certain aspects of the genomic landscape of the left breast tumors might be different from those of the right breast tumors in women aged ≤40 years. For example, the upregulation of 76 genes in young women with left BC correlated well with the reduced OS and RFS in patients with breast tumors of different subtypes. As the transcriptomic landscape of breast cancer is also known to be influenced by epigenetic modifiers and chromatin remodeling processes, as well as by the subcellular localization of ER-alpha, etc. [72,73,74,75], it would be important to further examine the contribution of these upstream modifiers of transcriptome in BCYW.

Our finding that many upregulated genes in BCYW that are also upregulated in normal breast tissues encode proteins secreted in EVs or body fluids such as urine, saliva, breast milk, and plasma is important as it raises the possibility of developing non-invasive predictive biomarkers for the general screening of younger women who are currently excluded from the guidelines to use mammography and/or individuals without predisposed breast cancer genes. Following a somewhat similar line of thinking, but in the context of prognosis, a recent study suggested differences in the nature of cargo EVs purified from the plasma of young BC patients as compared to those from healthy donors [76]. As a good percentage of breast cancers in young women are of the TNBC subtype, it would be important to understand the nature of biomaterial packed within the circulating EVs from different subtypes of BCYW. In this context, the nature of the exosomal microRNA profile of TNBC BC patients has been shown to be different from those of HER2 positive patients in neoadjuvant treatment studies [77], and it has been shown that EVs from certain TNBC cases have the potential to modify the tumor immune microenvironment in favor of an improved prognosis [78]. Likewise, increased levels of microRNA-1246 and microRNA-155 in circulating exosomes from HER2-positive, transtuzumab-resistant breast cancer patients have been shown to be different from transtuzumab-resistant cases [79], raising the possibility of using the content of EVs in predicting the acquired therapeutic resistance. Though these observations did not consider the 3 age groups of BC patients—the focus of the present study—they provide preliminary support for the need to examine the nature of EVs in future clinical trials with BCYW patients. In addition, these observations also suggest that certain dysregulated genes in BCYW could be involved in cellular communications and interactions between the normal and tumor tissues.

One of the continuing bottlenecks in BCYW research is the lack of a predictive gene signature or signatures about the clinical manifestation of the disease and/or predicting the probability of developing sporadic BCYW in healthy young women. The observations presented here provide clues that such gene signatures might not be universal in nature and are likely to be somewhat different for distinct geographical populations and further modified by the sub-types and/or pathobiology of BCYW. As the manifestation of transcriptome is tightly governed by upstream epigenetic regulatory pathways which in turn are known to be modified by lifestyle and environmental variables [80,81], the authors hypothesize that a portion of the genome is expected to be differentially modified/regulated in different geographic populations—presumably due to region- and/or population-specific lifestyle and epigenome–environmental interactions. Our observation that even the two largest publicly available breast tumor sequencing databases (i.e., TCGA and METABRIC) contain a limited number of adjacent matching normal breast tissues for patients aged ≤40 years points to a major challenge for using such datasets for developing such gene signatures, unless we substantially increase the sample-size of matching adjacent normal breast specimens as well as start including age-matched normal breast tissues from donors with no history of breast cancer. In this context, the current common approach of cross-comparison among tumors from different age groups has provided interesting insights of breast tumors as a function of age. However, this might not be sufficient to start developing predictive biomarkers for sporadic BCYW to capture the cases at early, precancerous stages among young women. From the point of evolving predictive biomarkers for the purpose of screening, it would be highly desirable to start developing non-invasive predictive biomarkers of BCYW. To this effect, the results presented here provide clues about the existence of relevant overexpressed gene products in human body fluids such as urine, saliva, breast milk, and plasma, and support the feasibility further developing such research themes.

From the clinical point of view, it would be important to identify shared genes and unique signature(s) for each of the three age groups, if any, in addition to BCYW aged ≤40 years, in the context of nodal status and tumor subtypes, and if such signatures in BCYW are affected by the menstrual cycle. In this context, one cannot rule out the possibility of over- and/or persistent stimulation of certain breast cancer-promoting genes via the hyperactivation of converging master cellular router pathways (i.e., inflammation, metabolic and immunologic responses) due to life-style variables such as stress, diet, and genotoxic environmental signals. In addition, it remains possible that exposure to certain environmental carcinogens in some individuals might be associated with the stimulation of cellular genes and/or transcriptional factors and, in turn, the activation of cancer-promoting pathways. For example, activation of the aryl hydrocarbon receptor (AHR) transcription factor by a range of upstream activators could promote its translocation to the nucleus, allowing its interactions with binding factors and binding to xenobiotic response elements in the target genes to stimulate the expression of target genes, including cytochrome P450, with roles in breast cancer progression [82]. At the moment, we do not understand the nature of such gene–environmental interactions as a function of age that might be preferentially engaged in BCYW. In conclusion, the study findings delineate the genomics of BCYW and provide several new insights into BCYW pathology, laying a foundation for the development of several dysregulated molecules as predictive or prognostic biomarkers for BCYW. The authors believe that scientific progress in further understanding the underlying reasons of the noticed increased or decreased expression of cancer-associated genes in the normal breast and, in turn, their causative relationship with specific life-style variables will constitute a quantum leap for BCYW research and treatment, and perhaps breast cancer at large.

## Data Availability

The data presented in this study are available in supplementary material.

## Supplementary Materials

The following supporting information can be downloaded at: https://www.mdpi.com/article/10.3390/cells11121927/s1, Supplementary Figures S1–S16 followed by Supplementary Tables S1–S10. Figure S1: Overlap of upregulated genes among ≤40, 41–54 and ≥ 55-years old age groups in in the TCGA breast carcinoma dataset, Figure S2: Overlap of upregulated genes among ≤40, 41–54 and ≥ 55-years old age groups in in the TCGA breast carcinoma dataset, Figure S3: Overlap of up (red)- and down (black)-regulated genes among ≤40, 41–54 and ≥ 55 years old age groups in the METABRIC breast tumor dataset, Figure S4: Overlap of upregulated genes among four sets of differentially expressed genes reported in young women breast cancer, Figure S5: Clinical significance of a subset of upregulated genes specific to ≤40 years with no overlaps with other age-groups, Figure S6: Heatmap representing the expression of indicated at the levels of mRNA and protein in the same set of TCGA breast cancer samples, using the dataset curated from the cBioPortal platform, Figure S7: Examples of genes upregulated in breast tumors but downregulated in adjacent normal breast tissues, Figure S8: Dysregulated genes in BCYW aged ≤40 years with DEGs specific to the menstrual cycle phases; Figure S9: Clinical significance of a subset of upregulated genes specific to ≤40 years in the METABRIC breast tumor dataset, Figure S10: Status of BCYW genes in intrinsic HER2 breast tumor subtype, Figure S11: Status of BCYW genes in intrinsic luminal A breast tumor subtype, Figure S12: Status of BCYW genes in intrinsic luminal B breast tumor subtype, Figure S13: Breast cancer genes in right breast tumors aged ≤40 years, Figure S14: Comparative analysis of dysregulated genes in matching normal specimens. Figure S15: Summary of exemplified comparative analyses to highlight the noticed overlap among secreted proteins by breast cancer cell lines, cancer cell lines, and three human body fluids such as urine, saliva and breast milk and plasma, Figure S16: Summary and overlap of secreted proteins, identified in the METABRIC dataset for breast tumors aged ≤40 years, by breast cancer cell lines, cancer cell lines, human body fluids such as urine, saliva and breast milk, and plasma. Table S1: Summary of clinical features of the breast tumor specimens in the TCGA Firehose legacy breast cancer study, Table S2: Gene Ontology biological function analysis of 45 mapped genes out of 60 upregulated genes in Figure 1a. Table S3: Gene Ontology biological function analysis of 14 mapped genes out of highly upregulated 17 genes in Figure 1e, Table S4: Gene Ontology biological function analysis of 19 mapped genes out of 22 upregulated genes in Figure 2b,c, Table S5: Gene Ontology biological function analysis of 8 mapped genes out of 12 downregulated genes in Figure 2g, Table S6: Gene Ontology biological function analysis of 26 mapped genes out of 27 upregulated genes in Figure 3b, Table S7: Gene Ontology biological function analysis of 95 mapped genes out of 124 upregulated genes in Figure 7b (left breast cancer), Table S8: Gene Ontology biological function analysis of 11 highly upregulated genes in Figure 7f, Table S9: Examples of secreted gene products relevant to the biology of BCYW, aged ≤40 years; Table S10: Examples of secreted gene products relevant to the biology of BCYW, aged ≤40 years.

## References

[1] Sung H., Ferlay J., Siegel R.L., Laversanne M., Soerjomataram I., Jemal A., Bray F. (2021). Global Cancer Statistics 2020: GLOBOCAN Estimates of Incidence and Mortality Worldwide for 36 Cancers in 185 Countries. CA A Cancer J. Clin..

[2] Hendrick R.E., Helvie M.A., Monticciolo D.L. (2021). Breast Cancer Mortality Rates Have Stopped Declining in U.S. Women Younger than 40 Years. Radiology.

[3] Kumar R., Abreu C., Toi M., Saini S., Casimiro S., Arora A., Paul A.M., Velaga R., Rameshwar P., Lipton A. (2022). Oncobiology and Treatment of Breast Cancer in Young Women. Cancer Metastasis Rev..

[4] Kim H.J., Kim S., Freedman R.A., Partridge A.H. (2022). The Impact of Young Age at Diagnosis (Age <40 Years) on Prognosis Varies by Breast Cancer Subtype: A U.S. SEER Database Analysis. Breast.

[5] Paluch-Shimon S., Cardoso F., Partridge A.H., Abulkhair O., Azim H.A.J., Bianchi-Micheli G., Cardoso M.-J., Curigliano G., Gelmon K.A., Harbeck N. (2020). ESO-ESMO 4th International Consensus Guidelines for Breast Cancer in Young Women (BCY4). Ann. Oncol..

[6] Bajpai J., Ventrapati P., Joshi S., Wadasadawala T., Rath S., Pathak R., Nandhana R., Mohanty S., Chougle Q., Engineer M. (2021). Unique Challenges and Outcomes of Young Women with Breast Cancers from a Tertiary Care Cancer Centre in India. Breast.

[7] Breast Cancer Incidence (Invasive) Statistics|Cancer Research UK. https://www.cancerresearchuk.org/health-professional/cancer-statistics/statistics-by-cancer-type/breast-cancer/incidence-invasive#heading-One.

[8] Okazaki M., Bando H., Tohno E., Kujiraoka Y., Iguchi-Manaka A., Ichioka E., Tsushima Y., Watanabe H., Hara H. (2021). Investigation of the Significance of Population-Based Breast Cancer Screening among Women Aged under 40 Years. Breast Cancer.

[9] Nakata K., Hiyama E., Katanoda K., Matsuda T., Tada Y., Inoue M., Kawa K., Maru M., Shimizu C., Horibe K. (2022). Cancer in Adolescents and Young Adults in Japan: Epidemiology and Cancer Strategy. Int. J. Clin. Oncol..

[10] Hayashi N., Kumamaru H., Isozumi U., Aogi K., Asaga S., Iijima K., Kadoya T., Kojima Y., Kubo M., Miyashita M. (2020). Annual Report of the Japanese Breast Cancer Registry for 2017. Breast Cancer.

[11] Alvarez-Bañuelos M.T., Segura-Jaramillo K.A., Gómez-Rivera E.D.C., Alarcón-Rojas C.A., Morales-Romero J., Sampieri C.L., Guzmán-García R.E. (2021). Age Under 30 Years As a Predictor of Poor Survival in a Cohort of Mexican Women With Breast Cancer. Cancer Control.

[12] Ntirenganya F., Twagirumukiza J.D., Bucyibaruta G., Rugwizangoga B., Rulisa S. (2021). Premenopausal Breast Cancer Risk Factors and Associations with Molecular Subtypes: A Case-Control Study. Int. J. Breast Cancer.

[13] Chen L.-J., Chang Y.-J., Chang Y.-J. (2021). Treatment and Long-Term Outcome of Breast Cancer in Very Young Women: Nationwide Population-Based Study. BJS Open.

[14] Conte B., Soldato D., Razeti M.G., Fregatti P., de Azambuja E., Schettini F., Prat A., del Mastro L., Lambertini M. (2022). De Novo Metastatic Breast Cancer Arising in Young Women: Review of the Current Evidence. Clin. Breast Cancer.

[15] Szollár A., Újhelyi M., Polgár C., Oláh E., Pukancsik D., Rubovszky G., Udvarhelyi N., Kovács T., Sávolt Á., Kenessey I. (2019). A Long-Term Retrospective Comparative Study of the Oncological Outcomes of 598 Very Young (≤35 Years) and Young (36–45 Years) Breast Cancer Patients. Eur. J. Surg. Oncol..

[16] Suter M.B., Pagani O. (2018). Should Age Impact Breast Cancer Management in Young Women? Fine Tuning of Treatment Guidelines. Ther. Adv. Med. Oncol..

[17] Azim H.A.J., Partridge A.H. (2014). Biology of Breast Cancer in Young Women. Breast Cancer Res..

[18] Jemal A., Siegel R., Xu J., Ward E. (2010). Cancer Statistics, 2010. CA Cancer J. Clin..

[19] Zhong W., Tan L., Jiang W.G., Chen K., You N., Sanders A.J., Liang G., Liu Z., Ling Y., Gong C. (2019). Effect of Younger Age on Survival Outcomes in T1N0M0 Breast Cancer: A Propensity Score Matching Analysis. J. Surg. Oncol..

[20] Park C., Yoon K.-A., Kim J., Park I.H., Park S.J., Kim M.K., Jang W., Cho S.Y., Park B., Kong S.-Y. (2019). Integrative Molecular Profiling Identifies a Novel Cluster of Estrogen Receptor-Positive Breast Cancer in Very Young Women. Cancer Sci..

[21] Yau C., Fedele V., Roydasgupta R., Fridlyand J., Hubbard A., Gray J.W., Chew K., Dairkee S.H., Moore D.H., Schittulli F. (2007). Aging Impacts Transcriptomes but Not Genomes of Hormone-Dependent Breast Cancers. Breast Cancer Res..

[22] Azim H.A.J., Nguyen B., Brohée S., Zoppoli G., Sotiriou C. (2015). Genomic Aberrations in Young and Elderly Breast Cancer Patients. BMC Med..

[23] Waks A.G., Kim D., Jain E., Snow C., Kirkner G.J., Rosenberg S.M., Oh C., Poorvu P.D., Ruddy K.J., Tamimi R.M. (2022). Somatic and Germline Genomic Alterations in Very Young Women with Breast Cancer. Clin. Cancer Res..

[24] Azim H.A., Peccatori F.A., Brohée S., Branstetter D., Loi S., Viale G., Piccart M., Dougall W.C., Pruneri G., Sotiriou C. (2015). RANK-ligand (RANKL) expression in young breast cancer patients and during pregnancy. Breast Cancer Res..

[25] Gu X., Wang B., Zhu H., Zhou Y., Horning A.M., Huang T.H.-M., Chen Y., Houghton P., Lai Z., Michalek J.E. (2020). Age-Associated Genes in Human Mammary Gland Drive Human Breast Cancer Progression. Breast Cancer Res..

[26] Colak D., Nofal A., Albakheet A., Nirmal M., Jeprel H., Eldali A., Al-Tweigeri T., Tulbah A., Ajarim D., Malik O. (2013). Age-Specific Gene Expression Signatures for Breast Tumors and Cross-Species Conserved Potential Cancer Progression Markers in Young Women. PLoS ONE.

[27] Anders C.K., Hsu D.S., Broadwater G., Acharya C.R., Foekens J.A., Zhang Y., Blackwell K.L. (2008). Young age at diagnosis correlates with worse prognosis and defines a subset of breast cancers with shared patterns of gene expression. J. Clin. Oncol..

[28] Kan Z., Ding Y., Kim J., Jung H.H., Chung W., Lal S., Cho S., Fernandez-Banet J., Lee S.K., Kim S.W. (2018). Multi-Omics Profiling of Younger Asian Breast Cancers Reveals Distinctive Molecular Signatures. Nat. Commun..

[29] Eiro N., Gonzalez L.O., Fraile M., Cid S., Schneider J., Vizoso F.J. (2019). Breast Cancer Tumor Stroma: Cellular Components, Phenotypic Heterogeneity, Intercellular Communication, Prognostic Implications and Therapeutic Opportunities. Cancers.

[30] Pirone J.R., D’Arcy M., Stewart D.A., Hines W.C., Johnson M., Gould M.N., Yaswen P., Jerry D.J., Smith Schneider S., Troester M.A. (2012). Age-Associated Gene Expression in Normal Breast Tissue Mirrors Qualitative Age-at-Incidence Patterns for Breast Cancer. Cancer Epidemiol. Biomark. Prev..

[31] Kang T., Yau C., Wong C.K., Sanborn J.Z., Newton Y., Vaske C., Benz S.C., Krings G., Camarda R., Henry J.E. (2020). A Risk-Associated Active Transcriptome Phenotype Expressed by Histologically Normal Human Breast Tissue and Linked to a pro-Tumorigenic Adipocyte Population. Breast Cancer Res..

[32] Hinck L., Silberstein G.B. (2005). Key Stages in Mammary Gland Development: The Mammary End Bud as a Motile Organ. Breast Cancer Res..

[33] Woodward T.L., Xie J.W., Haslam S.Z. (1998). The Role of Mammary Stroma in Modulating the Proliferative Response to Ovarian Hormones in the Normal Mammary Gland. J. Mammary Gland Biol. Neoplasia.

[34] Pardo I., Lillemoe H.A., Blosser R.J., Choi M., Sauder C.A.M., Doxey D.K., Mathieson T., Hancock B.A., Baptiste D., Atale R. (2014). Next-Generation Transcriptome Sequencing of the Premenopausal Breast Epithelium Using Specimens from a Normal Human Breast Tissue Bank. Breast Cancer Res..

[35] Goldman M.J., Craft B., Hastie M., Repečka K., McDade F., Kamath A., Banerjee A., Luo Y., Rogers D., Brooks A.N. (2020). Visualizing and Interpreting Cancer Genomics Data via the Xena Platform. Nat. Biotechnol..

[36] Colaprico A., Silva T.C., Olsen C., Garofano L., Cava C., Garolini D., Sabedot T.S., Malta T.M., Pagnotta S.M., Castiglioni I. (2016). TCGAbiolinks: An R/Bioconductor Package for Integrative Analysis of TCGA Data. Nucleic Acids Res..

[37] Ritchie M.E., Phipson B., Wu D., Hu Y., Law C.W., Shi W., Smyth G.K. (2015). Limma powers differential expression analyses for RNA-sequencing and microarray studies. Nucleic Acids Res..

[38] Heberle H., Meirelles G.V., da Silva F.R., Telles G.P., Minghim R. (2015). InteractiVenn: A web-based tool for the analysis of sets through Venn diagrams. BMC Bioinform..

[39] Curtis C., Shah S.P., Chin S.F., Turashvili G., Rueda O.M., Dunning M.J., Speed D., Lynch A.G., Samarajiwa S., Yuan Y. (2012). The Genomic and Transcriptomic Architecture of 2,000 Breast Tumours Reveals Novel Subgroups. Nature.

[40] Cerami E., Gao J., Dogrusoz U., Gross B.E., Sumer S.O., Aksoy B.A., Jacobsen A., Byearsne C.J., Heuer M.L., Larsson E. (2012). The CBio Cancer Genomics Portal: An Open Platform for Exploring Multidimensional Cancer Genomics DataThe CBio Cancer Genomics Portal: An Open Platform for Exploring Multidimensional Cancer Genomics Data. Cancer Discov..

[41] Lánczky A., Győrffy B. (2021). Web-Based Survival Analysis Tool Tailored for Medical Research (KMplot): Development and Implementation. J. Med. Internet Res..

[42] Ringnér M., Fredlund E., Häkkinen J., Borg Å., Staaf J. (2011). GOBO: Gene Expression-Based Outcome for Breast Cancer Online. PLoS ONE.

[43] Fredlund E., Staaf J., Rantala J.K., Kallioniemi O., Borg Å., Ringnér M. (2012). The gene expression landscape of breast cancer is shaped by tumor protein p53 status and epithelial-mesenchymal transition. Breast Cancer Res..

[44] Fonseka P., Pathan M., Chitti S.V., Kang T., Mathivanan S. (2021). FunRich Enables Enrichment Analysis of OMICs Datasets. J. Mol. Biol..

[45] Behan F.M., Iorio F., Picco G., Gonçalves E., Beaver C.M., Migliardi G., Santos R., Rao Y., Sassi F., Pinnelli M. (2019). Prioritization of Cancer Therapeutic Targets Using CRISPR-Cas9 Screens. Nature.

[46] George B., Pillai P.M., Paul A.M., Amjesh R., Leitzel K., Ali S.M., Sandiford O., Lipton A., Rameshwar P., Hortobagyi G.N. (2021). Cellular Fitness Phenotypes of Cancer Target Genes from Oncobiology to Cancer Therapeutics. Cells.

[47] Kalra H., Drummen G.P.C., Mathivanan S. (2016). Focus on Extracellular Vesicles: Introducing the Next Small Big Thing. Int. J. Mol. Sci..

[48] Nanjappa V., Thomas J.K., Marimuthu A., Muthusamy B., Radhakrishnan A., Sharma R., Ahmad Khan A., Balakrishnan L., Sahasrabuddhe N.A., Kumar S. (2014). Plasma Proteome Database as a Resource for Proteomics Research: 2014 Update. Nucleic Acids Res..

[49] Muthusamy B., Hanumanthu G., Suresh S., Rekha B., Srinivas D., Karthick L., Vrushabendra B.M., Sharma S., Mishra G., Chatterjee P. (2005). Plasma Proteome Database as a Resource for Proteomics Research. Proteomics.

[50] Azim H.A.J., Michiels S., Bedard P.L., Singhal S.K., Criscitiello C., Ignatiadis M., Haibe-Kains B., Piccart M.J., Sotiriou C., Loi S. (2012). Elucidating Prognosis and Biology of Breast Cancer Arising in Young Women Using Gene Expression Profiling. Clin. Cancer Res..

[51] Deng G., Lu Y., Zlotnikov G., Thor A.D., Smith H.S. (1996). Loss of Heterozygosity in Normal Tissue Adjacent to Breast Carcinomas. Science.

[52] Aran D., Camarda R., Odegaard J., Paik H., Oskotsky B., Krings G., Goga A., Sirota M., Butte A.J. (2017). Comprehensive Analysis of Normal Adjacent to Tumor Transcriptomes. Nat. Commun..

[53] Zhang L., Wang L., Yang H., Li C., Fang C. (2021). Identification of Potential Genes Related to Breast Cancer Brain Metastasis in Breast Cancer Patients. Biosci. Rep..

[54] Zhao Y., Zheng X., Zheng Y., Chen Y., Fei W., Wang F., Zheng C. (2021). Extracellular Matrix: Emerging Roles and Potential Therapeutic Targets for Breast Cancer. Front. Oncol..

[55] Medeiros P.J., Pascetta S.A., Kirsh S.M., Al-Khazraji B.K., Uniacke J. (2022). Expression of Hypoxia Inducible Factor-Dependent Neuropeptide Y Receptors Y1 and Y5 Sensitizes Hypoxic Cells to NPY Stimulation. J. Biol. Chem..

[56] Bhat R., Thangavel H., Abdulkareem N.M., Vasaikar S., de Angelis C., Bae L., Cataldo M.L., Nanda S., Fu X., Zhang B. (2022). NPY1R Exerts Inhibitory Action on Estradiol-Stimulated Growth and Predicts Endocrine Sensitivity and Better Survival in ER-Positive Breast Cancer. Sci. Rep..

[57] Liu L., Xu Q., Cheng L., Ma C., Xiao L., Xu D., Gao Y., Wang J., Song H. (2015). NPY1R Is a Novel Peripheral Blood Marker Predictive of Metastasis and Prognosis in Breast Cancer Patients. Oncol. Lett..

[58] Navarrete M.A.H., Maier C.M., Falzoni R., Quadros L.G.D.A., Lima G.R., Baracat E.C., Nazário A.C.P. (2005). Assessment of the Proliferative, Apoptotic and Cellular Renovation Indices of the Human Mammary Epithelium during the Follicular and Luteal Phases of the Menstrual Cycle. Breast Cancer Res..

[59] Olsson H.L., Olsson M.L. (2020). The Menstrual Cycle and Risk of Breast Cancer: A Review. Front. Oncol..

[60] Liang H., Xiao J., Zhou Z., Wu J., Ge F., Li Z., Zhang H., Sun J., Li F., Liu R. (2018). Hypoxia Induces MiR-153 through the IRE1α-XBP1 Pathway to Fine Tune the HIF1α/VEGFA Axis in Breast Cancer Angiogenesis. Oncogene.

[61] Li X., Tian R., Gao H., Yang Y., Williams B.R.G., Gantier M.P., McMillan N.A.J., Xu D., Hu Y., Gao Y. (2017). Identification of a Histone Family Gene Signature for Predicting the Prognosis of Cervical Cancer Patients. Sci. Rep..

[62] Xie W., Zhang J., Zhong P., Qin S., Zhang H., Fan X., Yin Y., Liang R., Han Y., Liao Y. (2019). Expression and Potential Prognostic Value of Histone Family Gene Signature in Breast Cancer. Exp. Ther. Med..

[63] Senie R.T., Rosen P.P., Lesser M.L., Snyder R.E., Schottenfeld D., Duthie K. (1980). Epidemiology of Breast Carcinoma II: Factors Related to the Predominance of Left-sided Disease. Cancer.

[64] Perkins C.I., Hotes J., Kohler B.A., Howe H.L. (2004). Association between Breast Cancer Laterality and Tumor Location, United States, 1994–1998. Cancer Causes Control.

[65] Wilting J., Hagedorn M. (2011). Left-Right Asymmetry in Embryonic Development and Breast Cancer: Common Molecular Determinants?. Curr. Med. Chem..

[66] Veltmaat J.M., Ramsdell A.F., Sterneck E. (2013). Positional Variations in Mammary Gland Development and Cancer. J. Mammary Gland Biol. Neoplasia.

[67] Barbara R.C., Piotr R., Kornel B., Elżbieta Z., Danuta R., Eduardo N. (2020). Divergent Impact of Breast Cancer Laterality on Clinicopathological, Angiogenic, and Hemostatic Profiles: A Potential Role of Tumor Localization in Future Outcomes. J. Clin. Med..

[68] Robichaux J.P., Hallett R.M., Fuseler J.W., Hassell J.A., Ramsdell A.F. (2015). Mammary Glands Exhibit Molecular Laterality and Undergo Left-Right Asymmetric Ductal Epithelial Growth in MMTV-CNeu Mice. Oncogene.

[69] Campoy E.M., Laurito S.R., Branham M.T., Urrutia G., Mathison A., Gago F., Orozco J., Urrutia R., Mayorga L.S., Roqué M. (2016). Asymmetric Cancer Hallmarks in Breast Tumors on Different Sides of the Body. PLoS ONE.

[70] Newton Y., Szeto C., Vaske C., Reddy L., Reddy S. (2019). Abstract 2533: The Genomic and Transcriptomic Landscape of Left versus Right Sided Breast Cancer in 410 Cases. Cancer Res..

[71] Sandiford O.A., Donnelly R.J., El-Far M.H., Burgmeyer L.M., Sinha G., Pamarthi S.H., Sherman L.S., Ferrer A.I., DeVore D.E., Patel S.A. (2021). Mesenchymal Stem Cell-Secreted Extracellular Vesicles Instruct Stepwise Dedifferentiation of Breast Cancer Cells into Dormancy at the Bone Marrow Perivascular Region. Cancer Res..

[72] Eswaran J., Cyanam D., Mudvari P., Divijendra S., Reddy N., Pakala S.B., Nair S.S., Florea L., Fuqua S.A., Godbole S. (2012). Transcriptomic landscape of breast cancers through mRNA sequencing. Sci. Rep..

[73] Nair S.S., Kumar R. (2012). Chromatin remodeling in cancer: A gateway to regulate gene transcription. Mol. Oncol..

[74] Nair S.S., Mishra S.K., Yang Z., Balasenthil S., Kumar R., Vadlamudi R.K. (2004). Potential role of a novel transcriptional coactivator PELP1 in histone H1 displacement in cancer cells. Cancer Res..

[75] Manavathi B., Acconcia F., Rayala S.K., Kumar R. (2006). An inherent role of microtubule network in the action of nuclear receptor. Proc. Natl. Acad. Sci. USA.

[76] Jordan K.R., Hall J.K., Schedin T., Borakove M., Xian J.J., Dzieciatkowska M., Borges V.F. (2020). Extracellular vesicles from young women’s breast cancer patients drive increased invasion of non-malignant cells via the focal adhesion kinase pathway: A proteomic approach. Breast Cancer Res..

[77] Stevic I., Müller V., Weber K., Fasching P.A., Karn T., Marmé F., Schem C., Schwarzenbach H. (2018). Specific microRNA signatures in exosomes of triple-negative and HER2-positive breast cancer patients undergoing neoadjuvant therapy within the GeparSixto trial. BMC Med..

[78] Tkach M., Thalmensi J., Timperi E., Gueguen P., Névo N., Grisard E., Sirven P. (2022). Extracellular vesicles from triple negative breast cancer promote pro-inflammatory macrophages associated with better clinical outcome. Proc. Natl. Acad. Sci. USA.

[79] Zhang Z., Zhang L., Yu G., Sun Z., Wang T., Tian X., Duan X., Zhang C. (2020). Exosomal miR-1246 and miR-155 as predictive and prognostic biomarkers for trastuzumab-based therapy resistance in HER2-positive breast cancer. Cancer Chemother. Pharmacol..

[80] Knower K.C., To S.Q., Leung Y.K., Ho S.M., Clyne C.D. (2014). Endocrine disruption of the epigenome: A breast cancer link. Endocr. Relat. Cancer.

[81] Remely M., Stefanska B., Lovrecic L., Magnet U., Haslberger A.G. (2015). Nutriepigenomics: The role of nutrition in epigenetic control of human diseases. Curr. Opin. Clin. Nutr. Metab. Care..

[82] Paris A., Tardif N., Galibert M.D., Corre S. (2021). AhR and cancer: From gene profiling to targeted therapy. Int. J. Mol. Sci..

