# Supplementary material for: Delineation of Pathogenomic Insights of Breast Cancer in Young Women
Source: Cells. 2022 Jun 15;11(12):1927. doi: 10.3390/cells11121927 (PMC9221490; doi:10.3390/cells11121927)
Supplement: Supplementary file 1 [file cells-11-01927-s001.zip › cells-1750338-supplementary.pdf]

SUPPLEMENTARY FILE

Delineation of Pathogenomic Insights of Breast Cancer in Young Women

Aswathy Mary Paul, Bijesh George, Sunil Saini, Madhavan Radhakrishna Pillai, Masakazu Toi, Luis Costa and Rakesh Kumar

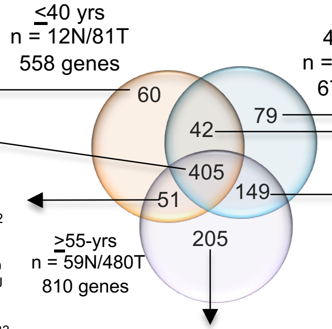

C17orf37
ZNF703
LLGL2
LAMP3
F5
ANO7
NME1
CENPW
IER5L
FOXS1
SLC38A5
B4GALNT3
COL1A2
HSPB1
H2AFX
COL5A2
C18orf56
KMO
KLHL35
SLC27A2
ADAMTSL2
PGBD5
KIAA1644
P2RY11
C4orf48
TLL2
SLC6A17
HAMP
CHST8
COL5A1
HAR1A
PTPRN
ATP6V0D2
C8orf73
CABP7
CLIC3
APOC2
GRIN2C
NLRP7
NPY1R
PLA2G2D
RHBG
CHRNA1
IL24
HIST1H2BH
C5orf46
HIST1H2AE
HCN4
PPEF1
TCN1
DUSP13
PGC
HIST1H2AM
C6orf223
DHRS2
IL19
TRPM5
AMTN
C1orf105
GABRA5

IL20
EDN2
GLDC
ROBO2
BUB1
CDK1
PCDH10
PPP2R2C
CDCA3
DLX5
WDR62
SALL4
HSD17B6
CNTD2
ANLN
ADAMTS14
EXO1
PITX1
VSTM2L
CD177
KIF2C
HTR1D
GPR37L1
CEP55
EMX1
DLGAP5
KIF26B
SOX11
AURKA
KCNK15
HAGHL
NDC80
CILP2
TPX2
BMPR1B
TDO2
KCNF1
SKA1
GABRD
TMEM90B
TK1
KIF18B
RRM2
NEIL3
SCUBE3
CDC20
KIF20A
FOXM1
AURKB
KREMEN2
PLK1
MS4A15
SYT13
CNTNAP2
PCSK1
NEK2
MELK
CXCL10
HIST1H2BC
FCRLB
FBN2
HJURP
ULBP1
ZPLD1
PLAC1
MUC5B
CDC25C
IQGAP3
MYBL2
CENPA
ISG15
TROAP
TUBB3
NUF2
HS6ST3
TMEM145
FOXJ1
KIF4A
XIRP1
ABP1
SIX3
RIMS4
GJB2
UNC13A
HHIPL2
KCNJ3
CEACAM6
HIST1H3H
CEACAM5
CBLN2
COMP
S100P
CA9
MYT1
RTBDN
CST1
PVALB
IBSP
CST4
CHRNA9
CDC20B
MUC2

ZNF695
ALG1L
JPH3
UPF0639
COL1A1
TNNT1
GPR158
CLEC5A
COL22A1
RECQL4
CCDC64
MAST1
PLAC2
CDCA2
ADORA2A
ONECUT2
KLK4
DQX1
FAM64A
TUBA3D
CAMP
SLC7A5
BMP8A
CENPF
AKR7A3
CST6
CLSPN
TTK
CDCA5
HIST1H2BD
SYT1
CXCR2P1
GPRIN1
CBX2
JAKMIP1
CDC45
HMMR
UBE2T
NCAPH
BCAS1
GAD1
CDKN3
TNFRSF9
ADAMDEC1
RAD54L
TUBA3E
RAMP1
DEPDC1
WISP1
MKRN3
ASPM
MATN3
IGFL2
TOP2A
P4HA3
SIX2
MMP12
DSCR6
FAM83D
CCNB2
CKAP2L
PBK
SAMD11
MCM10
SPC25
KIAA0101
KIF14
PTTG1
FN1
CDCA8
GTSE1
TFR2
E2F8
CCDC78
PSCA
BRSK2
ANK1
KLHDC7B
KIFC1
DIO1
NKAIN1
PKMYT1
A2ML1
CXCL9
INHBA
KIAA1199
WT1
SPC24
CAPN9
CACNG1
ELFN2
HCN2
INA
PPAPDC1A
COL10A1
LRTM2
CGA
MSMB
TLX1
VSTM2A
MAGEA1

OIP5
C16orf59
RAD51
CDT1
APLP1
HOXC13
SLC16A3
KPNA2
HN1
PNCK
NXPH4
KIF15
CAPS
CTHRC1
ESCO2
HOXC11
ESPL1
KIF23
CASC5
IL4I1
SDS
PAQR4
CCNE1
PRC1
EEF1A2
CTXN1
TACC3
PACSIN1
ERMN
NELL2
PIF1
IL21R
EME1
ZWINT
C20orf103
OASL
CENPE
IFI6
CDC6
E2F1
CCDC19
DEPDC1B
FAM54A
PRR11
CAMK2N2
UBE2S
ZBED2
RCOR2
CCL20
AMZ1
C19orf33
HIST1H2BJ
GNG4
FAM72B
ASF1B
NEURL
FAM72D
MND1
SKA3
CENPM
SPIB
HES6
E2F2
COX6C
FAM72A
E2F7
SGOL1
SYNGR3
CCNE2
PLA2G10
INHA
NCAPG
CCNA2
ESM1
BUB1B
MKI67
CDH2
NUSAP1
SPAG5
CXCL11
INSM1
UBE2C
SLC24A2
CCL11
BIRC5
C2orf54
KRT75
CNIH2
AIM1L
ART3
ATP1A3
ACAN
MMP11
GRM4
MMP13
MMP1
CHGB
ABCC13
CHGA
TRPA1
CST5

SLC44A4
C19orf21
GINS2
TYMS
TSPAN1
HMGB3
ADAM8
RNFT2
EZH2
MAD2L1
ORC1L
SYN1
GINS1
S100A14
F12
KIAA1524
C16orf75
ARHGAP11A
CACNA1H
ARTN
NUAK2
SHCBP1
MLF1IP
SBK1
OLR1
RET
DEGS2
METRN
C1orf135
ORC6L
RHBDL1
CDC25A
MAPK15
RAD51AP1
RAB26
LINGO1
SRMS
CREB3L1
C15orf42
HSH2D
GBP5
TRIP13
FAM57B
SPP1
LMNB1
GSDMC
KLHL38
MMP3
GRIN2D
ERCC6L
CELSR3
KIF18A
C1QTNF6
KIAA1211
KIAA0319
CSMD2
CKS2
TNFRSF18
SQLE
FOXP3
FAM111B
CD80
SLC1A1
TLCD1
KIF11
MAPK8IP2
POLQ
MFAP2
APOBEC3B
CCNB1
DTL
CENPI
KCNJ10
OTX1
RDH16
HIST2H4A
DIAPH3
GPRC5A
CD1A
SLC15A1
LRRC15
HIST1H3D
MMP10
GPR26
SERPINB7
KIF1A
HAPLN1
LOC84740
MYO3B
SYNPO2L
HORMAD1
CST2
COL11A1
HOXB13
NXPH1
EPYC
TRH
NCAN
TYRP1
ACTL8
PRSS33

RIBC2
SLC39A4
ANKRD22
FBXL16
DBNDD1
STARD10
HIST1H4J
ADM2
NFKBIL2
PAFAH1B3
PYCR1
ANXA9
C9orf140
CRABP2
C8orf85
PARD6B
HIST3H2A
NCRNA00176
HIST2H2BE
HIST1H2BK
EPN3
SRPK3
DOK7
HES2
PCSK9
CHST1
TMEM132E
CORIN
SLC7A11
SLITRK6
C3orf57
C9orf169
CACNG4
HIST1H1C
DRP2
TEX14
SLIT1
HIST1H2BG
ITPKA
NKX3-2
RIMS2
HIST1H4H
CCKBR
KISS1R
FIBCD1
KRT31
CSMD3
CST9
S100A7
ZDHHC22
PRSS1

SLC39A6
FREM2
TPRN
REPS2
FGFR4
ARX
CA14
C11orf82
F2RL2
RAG1AP1
GLYATL1
LOC153328
FABP7
GPR143
CCNI2
NDP
UNC5B
NEK5
PLK4
VSIG1
FOXA1
JPH1
TOX3
ERBB2
XRCC2
C2orf50
POLE2
DNAJC22
KIAA1467
FAM184B
CKMT1A
SUSD3
IGSF9
ECT2
SORD
CPA6
GPR84
TRIM59
RACGAP1
TRIM55
C9orf116
RSPH1
GPC2
GSG2
LRG1
PHGR1
FAM5B
CAPN13
PHF21B
FUT3
RHCE
FGD3
PPM1J
RAD54B
MTL5
MB
KIAA1257
PTK6
CYP24A1
PRRT3
PKIB
NUP210
TIGD3
TPD52
ATAD2
C15orf48
PDIA2
MRPS30

OBP2B
C16orf71
RHPN1
KCNC1
TMEM132A
ARHGAP11B
BAI2
C6orf154
DPEP1
PGLYRP2
HPX
MORN3
NPNT
MEX3A
TPRG1
KLK13
RP1
CRYM
ATP2A1
FLT3
LCN12
LYPD1
HPN
GINS4
HDC
KNDC1
RTKN2
MAG
ACCN1
KLRG2
KCNG1
TDRD12
GALNT5
NEFH
SERPINA11
SYT9
C9orf172
SPATA17
NEB
PLA2G3
BARX1
HELLS
CENPK
DNAJC12
MYO18B
KCNQ5
SHISA9
SNAP25
LEF1
CASKIN1
C10orf82
POSTN
SLC12A1
COL11A2
AGR3
TPSG1
CALML5
LRRC46
CHRDL2
TDRD1
UNC80
IP6K3
FLJ45983
AGR2
NPTX1
VWA5B2
ARNT2
FOXA3

TCAP
LOC643719
C12orf69
ZMAT4
UGT1A6
ANKRD30B
KLK14
CDSN
ACMSD
CPNE7
SERPINA6
PTCHD2
GTSF1
HEPHL1
LRRC31
C2CD4A
FGB
TGM3
TFF1
ECEL1
FGL1
SLC26A9
FGA
PCSK2
ADAMTS19
GATA5
PZP
PEX5L
B4GALNT2
FAM5C
FGG
MYH2
CTSE
CA10
SERPINB3
IVL
EPO
MYL2
SLC1A6
GDPD2
IRS4
XIRP2
SERPINB4
NDST4
DCD
SMR3B
XAGE1D
NRAP
ROS1
TGM4
NKX2-2
GNG13
SMR3A
FAM25A
CSAG3
CKM
C4orf40
CYP2A7
OLFM3
GNAT3
TBX10
GPR128
GABRA1
MYL1
CSAG1
S100A7A
GAGE1
MYH7
UCN3

CWH43
FANCI
POC1A
LAG3
SLC23A1
UNC5A
WDR72
CCNO
PRR15
SPEF1
TFF3
KCNE4
BRIP1
SPAG6
ETV7
SIM2
BLM
GP2
MUC1
FANCA
IGFALS
CIT
THPO
STIL
TMPRSS6
KCNH2
ZMYND10
FBXO43
FGFR3
HOTAIR
PNMT
OGDHL
C3orf67
MYBL1
PAX2
RPLP0P2
GDF15
SSTR2
F7
PXDNL
HOXC10
SLC16A6
GDF9
ENTPD8
SCG3
FSIP1
CLGN
VTN
CCDC151
UMODL1
C19orf51
PPM1E
PAH
ACTA1
CFB
GPR19
SLC8A2
NTNG1
TMPRSS4
FAM83A
ELAVL2
SCG2
KRT86
CRISP3
CXCL17
PADI3
DNAH11
CXCL13
CXADRP3
ZIC2
ZIC1
CYP2A6
KLK12
ACTN2
COL2A1
TPH1
SAMD14

S100A9
ARMC3
MSLN
NBPF6
GRIA2
SLC5A8
BEX1
HOXB9
CYP2B7P1
SMC1B
PCSK1N
FAM135B
SLC4A10
NBPF4
SEZ6L
UGT2B4
RUNDC3A
MAGEC1
SBSN
NAT1
TRIM67
UPK1A
S100A8
KCNH1
COL9A1
SOX2
FAM196A
PAX7
LOC152225
CAPSL
C20orf114
MAGEA4
TNNI3
CLDN6
PYDC1
MAEL
KRT20
CTAG1B
MS4A8B
GABBR2
SLC6A4
ZFP42
MAGEA9B
CLEC3A
LIN28A
SEZ6
DPYSL5
NELL1
GATA4
HMP19
NCRNA00052
SYT4
PRAME
ORM2
S100G
MAGEC2
SLC30A8
ORM1
SPDYC
TCL1B
ASCL1
CASP14
CARTPT
CPLX2
MAGEA2
KLHL1
MAGEA6
MAGEA3
GAGE2D
MAGEA12
GAGE4
GAGE12D

SULF1
BCL2A1
TIGIT
LRP8
IFI27
DYDC2
TNFSF4
TAS1R3
ULBP2
IGF2BP3
RLTPR
MGAT5B
ECM1
CHST6
CLDN9
ASPN
LTB
EN1
SLC17A9
ANKRD1
BGN
IDO1
MMP9
CRIP1
SPOCD1
COL9A3
ASCL2
CDKN2A
NIPAL4
NCCRP1
PSORS1C2
GAL
NPW
TRPM8
NMU
MLC1
CTNNA2
LOC400696
UCA1
Mar-04
CDH7
CPB1

SELL
ADAM19
STAP1
FBN3
PCP4
PDCD1
NOXO1
CCR7
KRT81
ACSL6
ACY3
OPRK1
UGT3A2
VCAN
AGT
TREM1
TM7SF4
ICOS
GZMB
HOXA11
FAP
DLX2
UBD
CCL19
FCGR1A
LBP
CCR4
ABHD12B
AQP9
GRB14
PNLDC1
CR2
ROPN1L
GJB6
FNDC1
CEACAM7
MS4A1
AIM2
CXCR5
TMEM92
IL12RB2
DKK1
CLIC6
FCRL3
RGAG1
SLC6A15
DMBT1
PCDH8
MNX1
LHFPL4
CD19
KRT4
MUC13
NPTX2
CNGA3
GCNT3
RHCG
VMO1
TCAM1P
C4orf7
PNMA5
TCL1A
CADPS
PAX1
CACNG6
GNG3
TMEM215
AICDA
CXorf61
CLVS2
VGF
C20orf85
SYT5
NEUROD1
LHX1
PLUNC
GLYATL3
CT45A1
SPANXA2

Supplementary Figure S1: Overlap of upregulated genes among ≤40, 41-54 and ≥ 55-years old age groups in the TCGA breast carcinoma dataset. Cut-off, log 2-fold or more change.

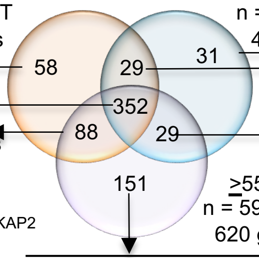

TRHDE
FCGR3B
CSRNP3
TNFSF11
CXCR2
DACT2
TP53AIP1
SILV
LPPR1
MYH1
SYCE1
ANKRD53
LRRTM2
TMEM213
SCN7A
ABCC6
C11orf92
PAR5
CRTAC1
ANO3
CRISPLD1
IL22RA2
RIC3
ZNF483
EGFR
ALDOC
SLC5A1
OXGR1
FLJ13197
HSPA12A
MAML2
PLEKHH2
SOX5
VAT1L
FCER1A
RCAN1
KCTD14
HOXA9
NLGN1
RHOU
APOD
PTCH2
RUNDC3B
GPM6B
CCBE1
LOC100126784
RNF157
PFKFB3
CCDC36
CCDC158
PIWIL4
CRYBG3
CDC14B
EGFLAM
CD164L2
KCNS1
IL-17RD
CALML3

HBA1
GLYAT
LEP
HBB
MRAP
GPD1
CIDEC
C14orf180
PLIN4
AQP7
PLIN1
HBA2
APOB
KCNIP2
PCK1
ACVR1C
TUSC5
ADIPOQ
SCARA5
AQPEP
LOC283392
CA4
HEPACAM
LGALS12
HSD17B13
NNAT
TNMD
HEPN1
ALDH1L1
ADH1B
ADH1A
HSPB7
CD300LG
FIGF
SLC19A3
FABP4
SMYD1
RDH5
SLC2A4
KLB
TMEM132C
LIPE
GPAM
HSPB6
RBP4
PCOLCE2
LYVE1
ANGPTL7
KIAA0408
CIDEA
FHL1
CES1
SLC7A10
DEFB132
OXTR
G0S2
TIMP4
LPL
CHRDL1
ABCD2
GYG2
ACADL
NPY2R
ITIH5
FXYD1
SDPR
MYOM1
LOC728264
ABCA8
TNXB
IL6
MAMDC2
PER1
COL17A1
RELN
ABCA6
SVEP1
ACSL1
NDRG2
DTX1
FAM13A
CX3CL1
SRPX
AKAP12
SPRY2
CLDN5
PDZD2

BTNL9
AOC3
PKHD1L1
FAM196B
CA3
RYR3
CXCL2
CD36
GRIA4
ABCA10
PFKFB1
ACSM5
WIF1
ITGA7
ADH1C
ABCA9
PPP1R1A
PRRT4
COL6A6
KLHL31
PDK4
SORBS1
C2orf40
MLXIPL
CFD
AKR1C2
GPX3
PCDH9
ATOH8
MME
PYGM
KCNE1
DPT
KLF15
ZBTB16
GPIHBP1
ANGPTL1
ACACB
NPR1
GDF10
PDE1C
CKMT2
AKR1C1
KIT
CLEC3B
CAV1
PDE3B
GPLD1
MMRN1
HIF3A
FOSB
C10orf90
ATP1A2
PI16
SLC16A7
GPR109A
KCNB1
BHMT2
CDO1
CLDN19
NAALAD2
NAT8L
C3orf55
SGK2
NRG2
LRRN4CL
PPARG
CCL14
ANGPT1
FMO2
TP63
NRG1
RNF150
SCN4B
FHL5
ASAM
ITM2A
BMP2
PRKAR2B
MMD
RGS6
FAM189A2
DGAT2
HOXA4
TMEM220
MEOX1

TMEM37
NTRK2
SCN2A
SCN4A
NMUR1
SAMD5
TGFBR3
PENK
C6
GCOM1
FGF2
MAOA
CHL1
DMGDH
KY
MASP1
S100B
LRRC2
TMEM178
PLAC9
SAA1
DMD
C14orf139
PIK3C2G
BMX
PDE2A
GPC3
EBF1
PLXNA4
FREM1
HLF
SFRP1
GHR
SLC25A27
SEMA3G
PAMR1
CCDC69
ADRB2
MATN2
GALNTL2
ADAMTS5
NIPSNAP3B
ALDH1A2
CHST9
PALMD
TNS1
ADAM33
MYH11
F10
EBF3
SCN2B
THRSP
CAV2
ARHGAP20
ALDH1A1
IGFBP6
AOX1
AK5
DARC
C7orf41
XPNPEP2
HOXA5
DNASE1L3
PKD1L2
SCN3A
LOC339524
IGSF10
LIFR
KLHL29
SLC22A3
GIPC2
EGR1
VIT
GPR146
EGR3
RERGL
ENPP2
C7orf58
RGN
NR3C2
C14orf49
FZD4
SLC17A7
C21orf34
PGM5
AGAP11

GPR109B
EGR2
ATF3
SEMA3D
TMEM100
GSN
RSPO3
LGI4
FAM70A
ANKRD29
LMOD1
IQSEC3
FAT2
IGF1
HSPB2
PTH1R
GPRASP1
PPP1R14A
MFAP4
CLDN11
WDR86
KLF4
KLHL13
ADHFE1
GNAI1
CORO2B
C8orf79
RBMS3
HAS3
ECM2
GPR133
TCEAL7
ITPRIPL1
GRRP1
KCNMB1
GLDN
COX7A1
KRT14
FGF1
USHBP1
PEAR1
FOXP2
MEG3
CNTFR
P2RY12
EDNRB
ADAMTS1
BBOX1
ROBO3
FAM107A
NCRNA00086
SLC27A6
GNAL
LRFN5
JPH2
TWIST2
PPP1R12B
MYLK
C2orf88
MOSC1
MEOX2
CRYAB
IL33
CCL28
EPHB1
CNN1
KL
LEPR
CD209
SAA2
FAM89A
SYNM
DMRT2
CASQ2SIK2
DDR2
TSHZ2
INMT
EFHA2
ABCB1
TACR1
DST
FOS
FAM13C
LRRC70
D4S234E
ISM1
C10orf10
CCDC3
AKR1C3
PREX2
CLDN8
CNTNAP3

C10orf128
RBP7
FGFBP2
LDB2
PALM2-AKAP2
PRELP
RASSF9
CEBPA
DUSP1
ACO1
ALPK3
EMCN
CUBN
CSRNP1
LPHN3
SELP
FOLR2
ZFP36
CXorf36
SHE
GNG11
MYOC
SLC26A3
ASPA
KCNJ16
TF
C1QTNF7
PROL1
TSPAN7
C7
SOD3
C4BPA
PTGFR
MT1M
LILRB5
PLCXD3
IGJ
HSD11B1
APOLD1
SCN3B
EMX2OS
AOC2
CYP26B1
THSD7B
GFRA2
ODZ1
C2CD4B
FAM149A
PID1
LRRN3
OGN
DENND2A
ABCC9
GGTA1
DOCK11
HOXA7
NR4A3
TXNIP
ANK2
PKDCC
EBF2
TBX15
TNNT3
CPM
APCDD1
ALDH2
MAPK10
SOX17
TNFRSF10D
AQP1
MCAM
MRC1
TMEM22
MOCS1
RAPGEF3
ABI3BP
TAL1
MT1X
HOXA3
CETP
GNG2
CAT
MRAS
KANK1
TMEM88
WASF3
OSR1
S1PR1

CSN1S1
C12orf39
LALBA
CSN2
PRG4
HAS1
AVPR2
GPBAR1
TRDN
SCN9A
C13orf33
CALB2
NRN1
ACSM1
EGFL6
MRGPRF
CDKN1C
ANXA1
MMP28
BMP6
RGS2
KCNA6
ANGPTL4
CSN3
NMB
TGFBR2
C9orf125
PTGIS
ACTG2
KRT17
LIMS2
LTBP4
CCL13
SLIT3
VWF
CREB5
LHFP
SLC14A2
KANK3
PTX3
CDR1
TYRO3
CD34
KLHL4
GYPC
SOSTDC1
NPR3
SOCS3
STX11
MAP7D3

SV2B
TMTC1
GREM2
IL17D
NECAB1
EHD2
PLSCR4
ADAMTSL4
ST6GALNAC3
PROS1
MARCO
MMRN2
ROBO4
KCNAB1
NDN
DCN
ADCY4
STAC2
C1QTNF1
ARHGEF15
SOX7
MFAP5
PTRF
NMT2
JAM2
CTSG
CFP
RASD1
PPP1R16B
CLIC5
TFPI
ECSCR
EPAS1
ID4
MYCT1
CCDC80
HP
ARHGAP36
PDE1B
PNPLA2
CCDC85A
SNCA
CELF2
TMOD1
CACNA2D1
CD248
FBLN5
PPAP2B
SPTBN1
GIMAP8

CXCL12
PCDH18
PPAP2A
WISP2
CCDC46
CYYR1
PVRL3
SYNPO
ANGPTL2
C20orf160
RUNX1T1
SH3D19
FERMT2
CNRIP1
SNCG
GPR116
LOC158376
AGPAT2
SPRY1
WSCD1
ANKDD1A
PCSK5
PCDHGA3
TRPM6
CBLN4
PTPRB
IRX1
TEK
P2RY14
FAM126A
VGLL3
LHX6
TINAGL1
HYAL1
EMP1
SH3BGRL2
CFL2
XG
LAMA2
ELOVL3
KLF2
CXCR7
CYTL1
TRIM29
AIFM2
NPR2
PLAGL1
TLN2
BOC
RECK
TACC1

GSTM5
SULT1C3
EDN3
PTN
SGCG
ASTN1
LUZP2
UGT2B28
MUSTN1
PHYHIP
SLC29A4
CADM3
NGFR
CCL21
GABRE
BMPER
CDH23
KRT5
HSD3B2
CITED1
CCDC8
IRX6
NR4A1
MGLL
PDE11A
CACHD1
APCDD1L
SYNPO2
C5orf4

STAB2
CRHBP
ABCB5
PIGR
SLCO1A2
SLC7A3
SPHKAP
GFAP
PAK7
SLC13A2
TSLP
RASL10A
CAPN11
EFCAB1
PI15
DLK2
KRT15
RSPO1
EMILIN3
EGF
MYEOV
KCNA1
KCNH8
FGFBP1
HRASLS5
BCHE
PROX1
ATP13A5
LAMA3

SFTPB
ITIH2
MYOM2
ALB
DES
HS3ST4
TESC
SCGB1A1
SCGB3A1
AFP
C6orf223
LST-3TM12
PAK3
BMP3
KLK3
ACSM3
ODZ2
SLC16A12
PTCHD1
GSTT2
CACNA1G
HAP1
LAMC3
FAM150B
AKR1B15
PLCH2
TCEAL5
IL17B
TAT
LOXL4
DBC1

Supplementary Figure S2: Overlap of downregulated genes among ≤40, 41-54 and ≥ 55-years old age groups in the TCGA breast carcinoma dataset. Cut-off , log 2-fold or more change.

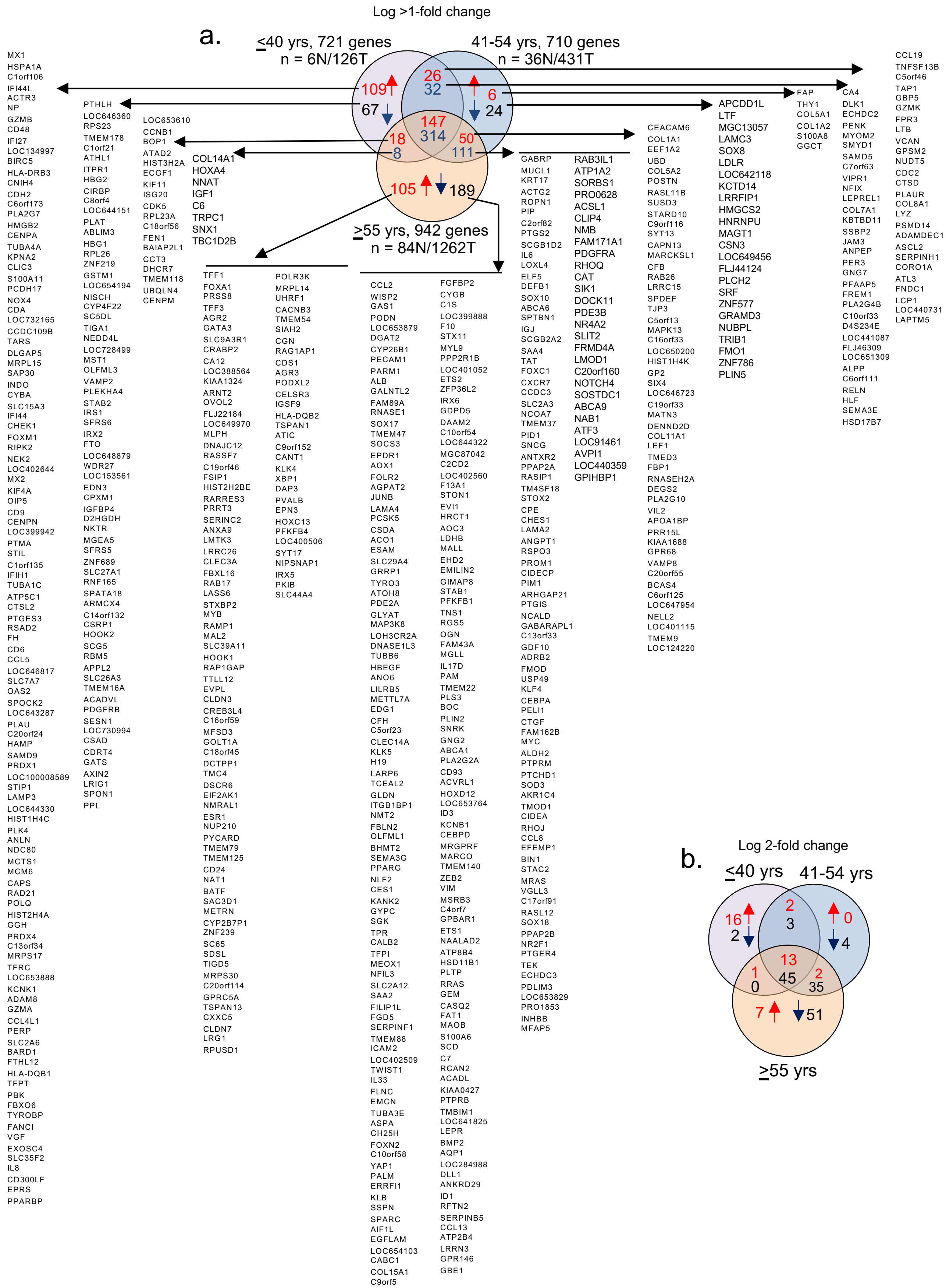

Supplementary Figure S3: Overlap of up (red)- and down (black)-regulated genes among ≤40, 41-54 and ≥ 55 years old age groups in the METABRIC breast tumor dataset. Cut-off, log 1-fold or more (a) or log 2-fold or more (b) change.

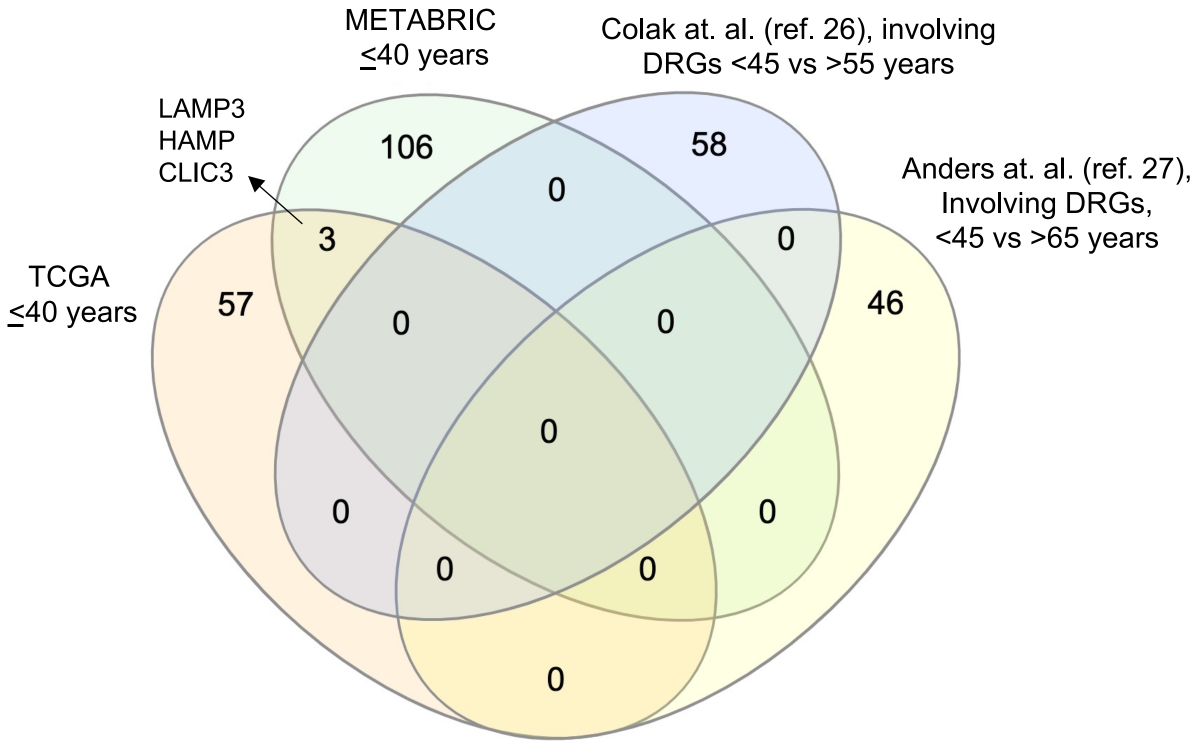

Supplementary Figure S4: Overlap of upregulated genes among four sets of differentially expressed genes reported in young women breast cancer.

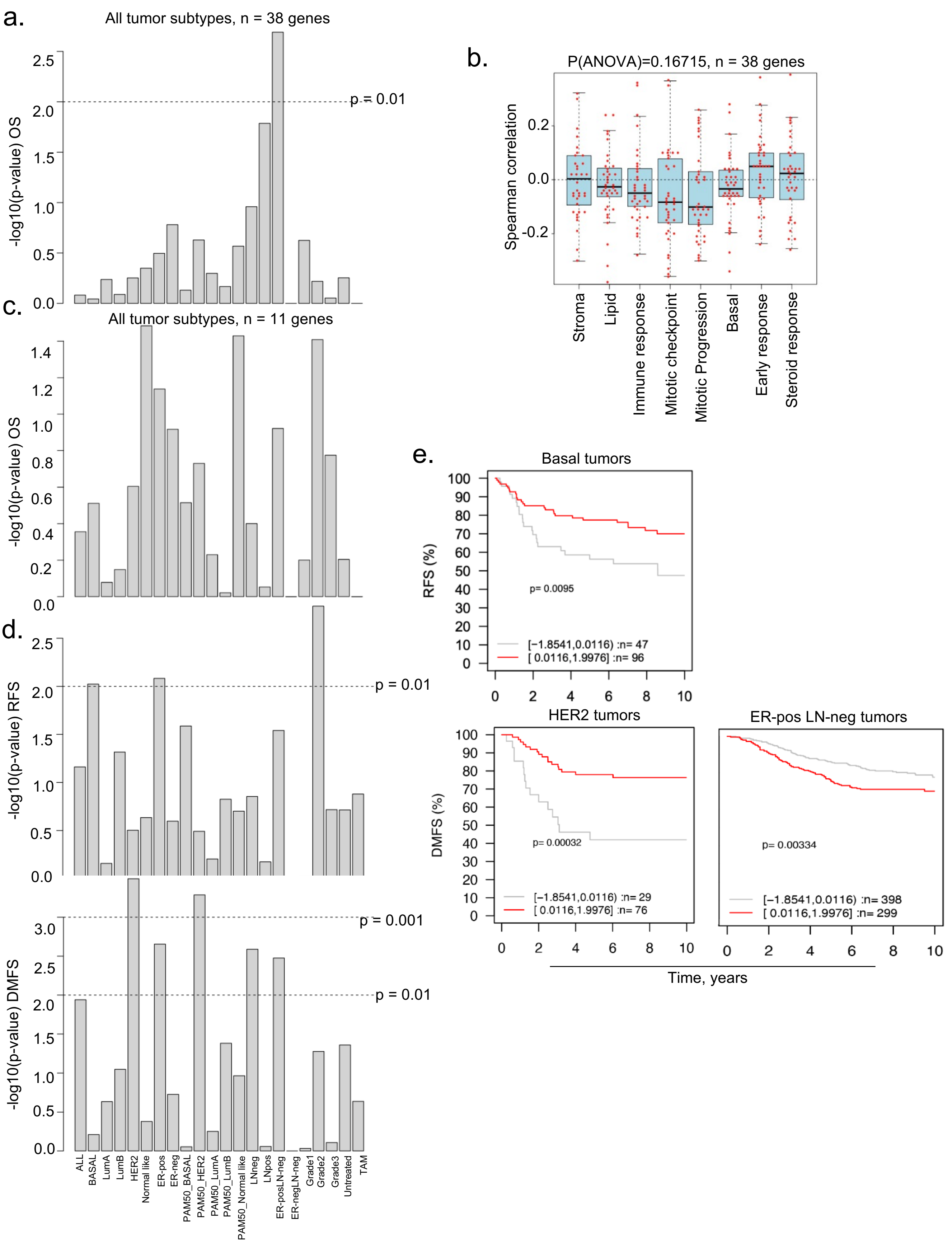

Supplementary Figure S5: Clinical significance of a subset of upregulated genes specific to ≤40 years with no overlaps with other age-groups. **a**, Kaplan-Meier OS summary of 38 out of 60 upregulated genes mapped in GOBO database with 1881 breast tumors; **b**, spearman correlation analysis of 38 genes in the context of co-expressed 8 gene modules; **c** and **d**, Kaplan-Meier OS, RFS and DMFS summaries of 11 out of 17 shortlisted genes in Fig. 1e, mapped in the GOBO database with 1881 breast tumors; **e**, RFS and DMFS curves using the high (red) and low (gray) expression of 11 genes for indicated breast tumor subtypes. All survival, correlation analysis performed using the online GOBO tools.

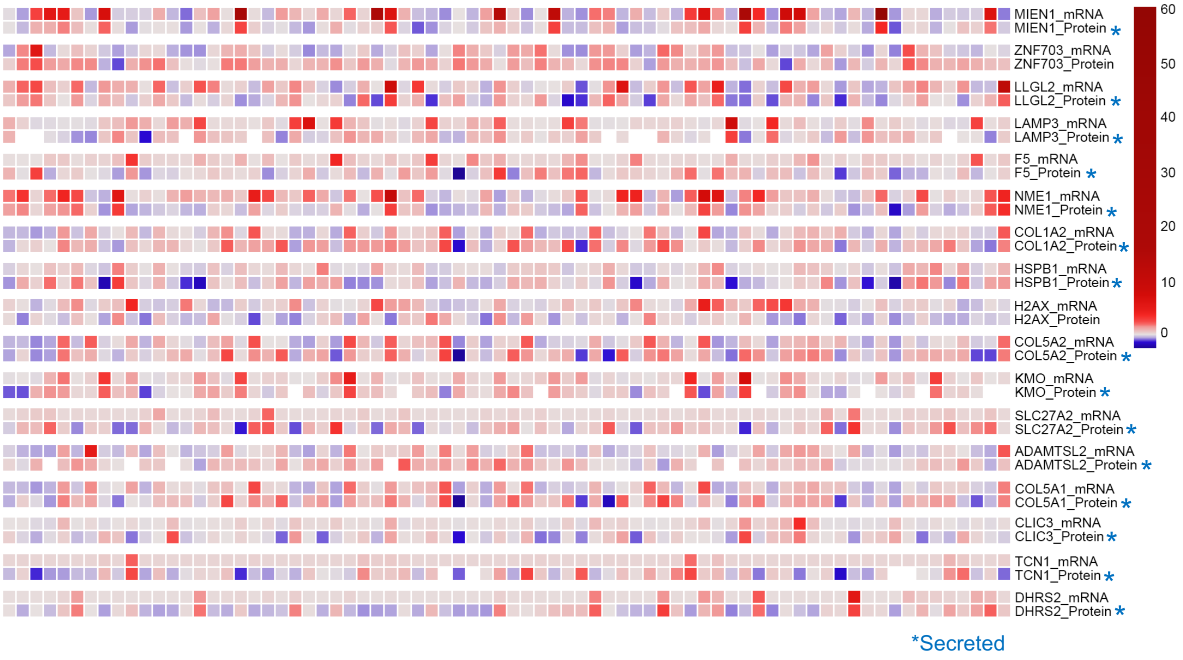

Supplementary Figure S6: Heatmap representing the expression of indicated at the levels of mRNA and protein in the same set of TCGA breast cancer samples, using the dataset curated from the cBioPortal platform.

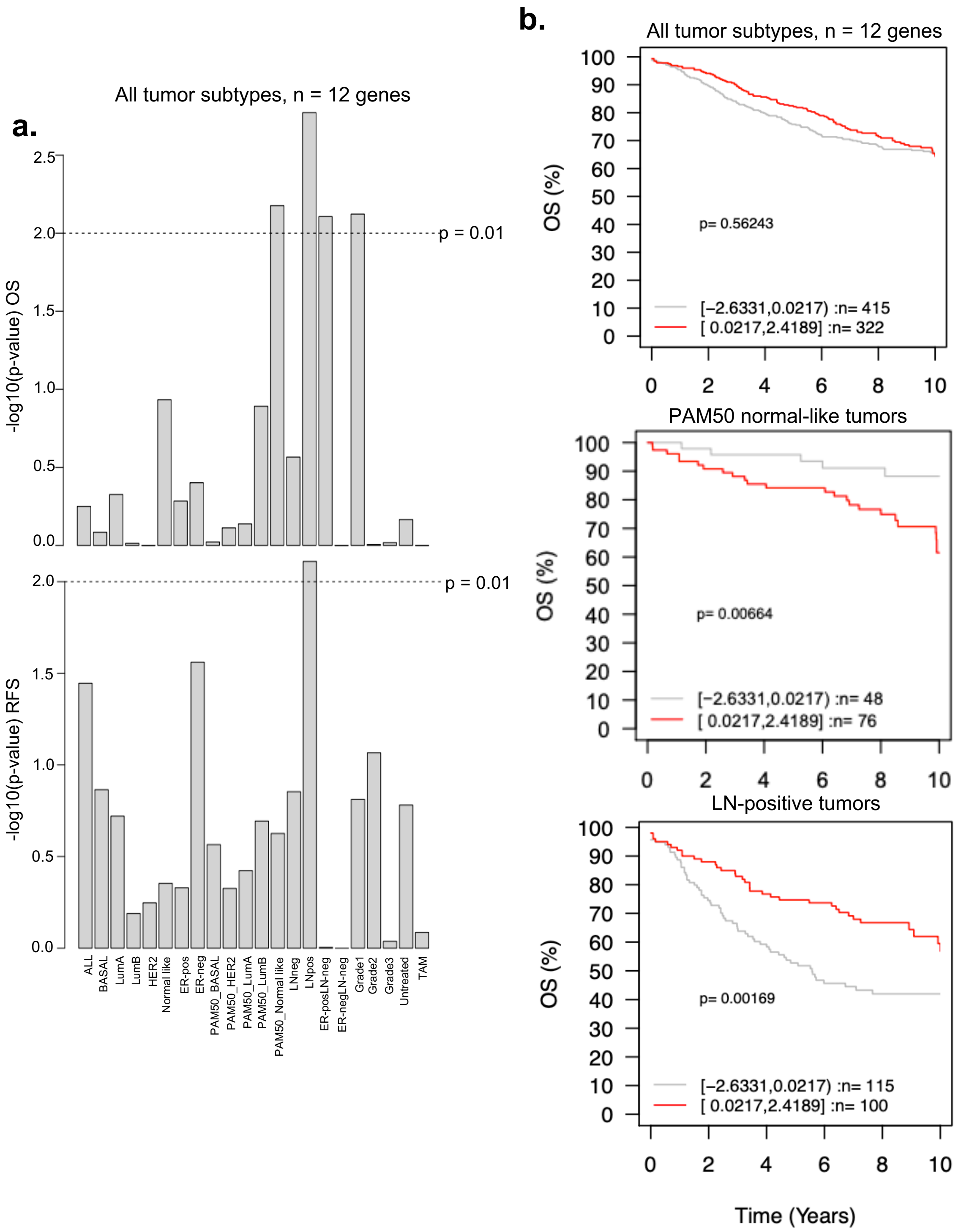

Supplementary Figure S7: Examples of genes upregulated in breast tumors but downregulated in adjacent normal breast tissues. **a**, Kaplan-Meier OS and RFS summaries of 12 out of 20 upregulated genes in Fig. 2b. **b**, OS curves using the high (red) and low (gray) expression of 12 genes for all tumor sub-types, PAM50 normal-like tumors and LN-positive tumors. All survival, correlation analysis performed using the online GOBO tools.

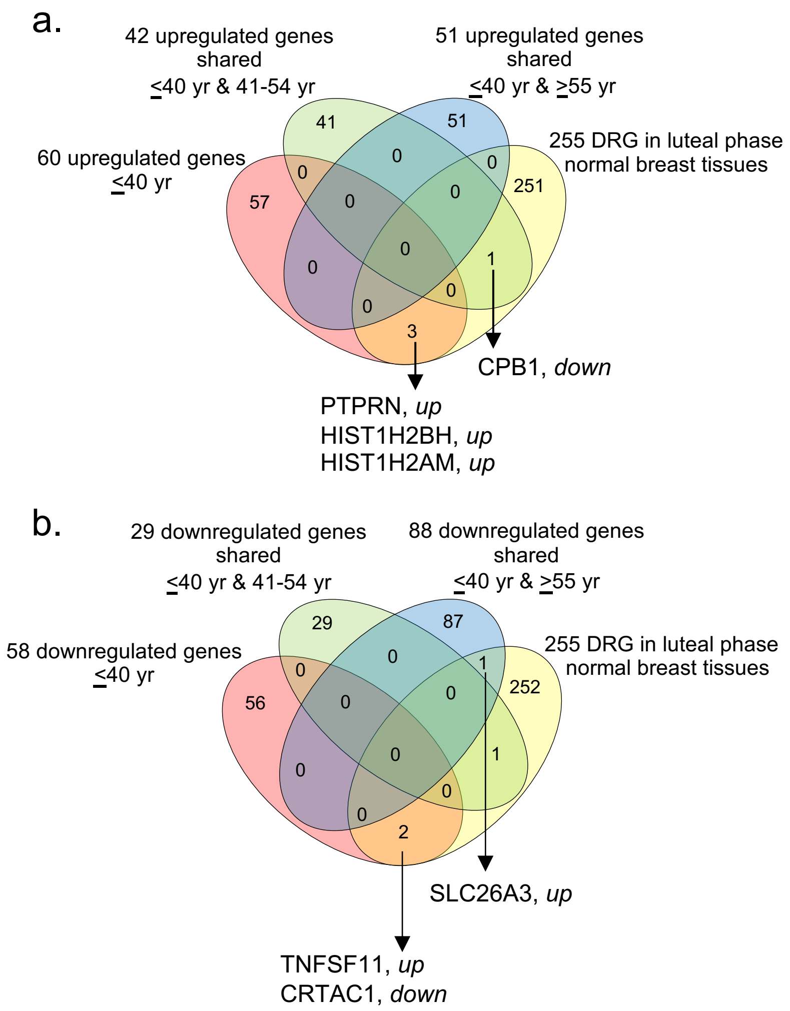

Supplementary Figure S8: Dysregulated genes in BCYW aged ≤40 years with DEGs specific to the menstrual cycle phases. **a**, overlap among DRGs in normal breast tissues during the luteal phase of the menstrual cycle and upregulated in breast tumors from women aged ≤40 years or shared with women between the ≤40 and 41 - 54 years or ≤40 and ≥55 years. **b**, overlap among DRGs in normal breast tissues during the luteal phase of the menstrual cycle and downregulated u in breast tumors from women aged ≤40 years or shared with women between the ≤40 and 41 - 54 years or ≤40 and ≥55 years.

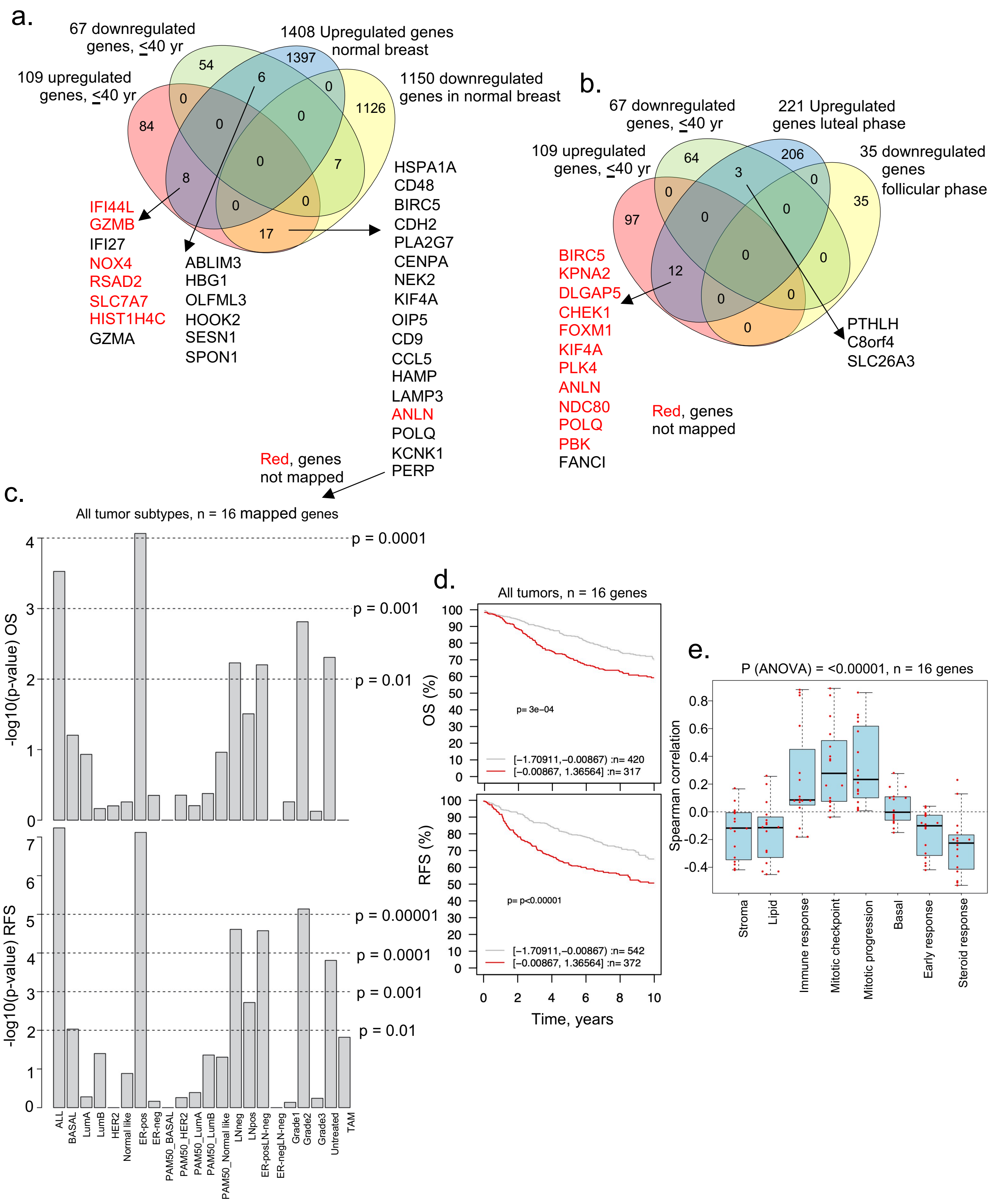

Supplementary Figure S9: Clinical significance of a subset of upregulated genes specific to ≤40 years in the METABRIC breast tumor dataset. **a**, Overlap of up- and down-regulated genes in patients aged ≤40 years and modulated genes in normal breast tissues; **b**, Overlap of up- and down-regulated genes in patients aged ≤40 years and DRGs in normal breast tissues from the luteal or follicular phases of the menstrual cycle; **c**, Kaplan-Meier OS and RFS summaries of 16 genes upregulated in breast tumors <40 years but downregulated in normal breast tissues; **d**, OS and RFS curves using the high (red) and low (gray) expression of 16 genes for all tumor subtypes. **e**, Spearman correlation analysis of 16 genes in the context of co-expressed 8 gene modules. All survival, correlation analysis performed using the online GOBO tools.

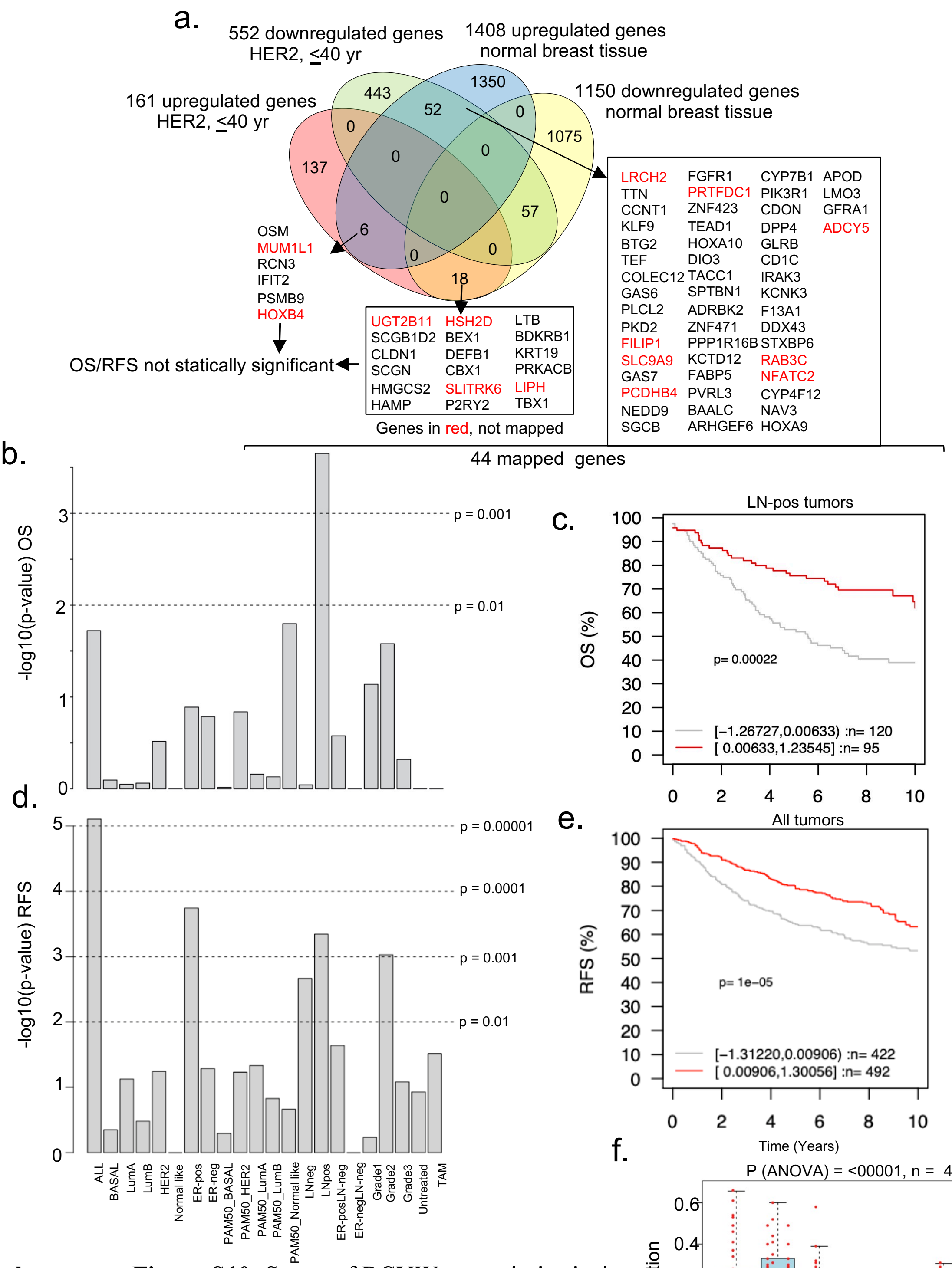

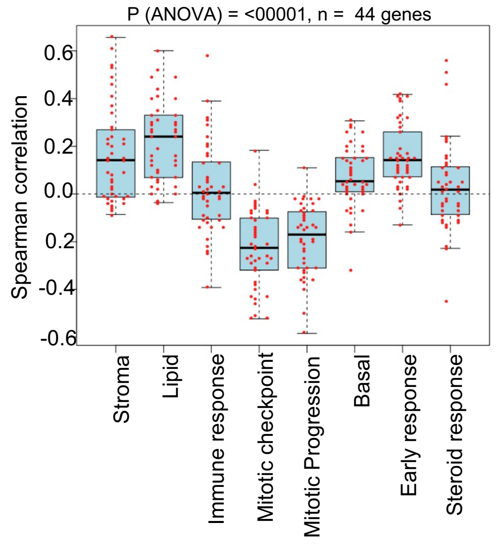

Supplementary Figure S10: Status of BCYW genes in intrinsic HER2 breast tumor subtype. **a**, Overlap of up- and down-regulated genes in HER2 breast tumors from patients aged ≤40 year with upregulated or downregulated genes in normal breast tissues; **b**, Kaplan-Meier OS summary of 44 genes downregulated in HER2 tumors but upregulated in normal breast tissues mapped in GOBO database with 1881 breast tumors; **c**, OS curve using the high (red) and low (gray) expression of 44 genes in LN-pos breast tumors; **d**, Kaplan-Meier RFS summary of 44 genes mapped in GOBO database with 1881 breast tumors; **e**, RFS curve using the high (red) and low (gray) expression of 44 genes in all tumor subtypes; and **f**, Spearman correlation analysis of 44 genes in the context of co-expressed 8 gene modules. All survival, correlation analysis performed using the the GOBO online tools.

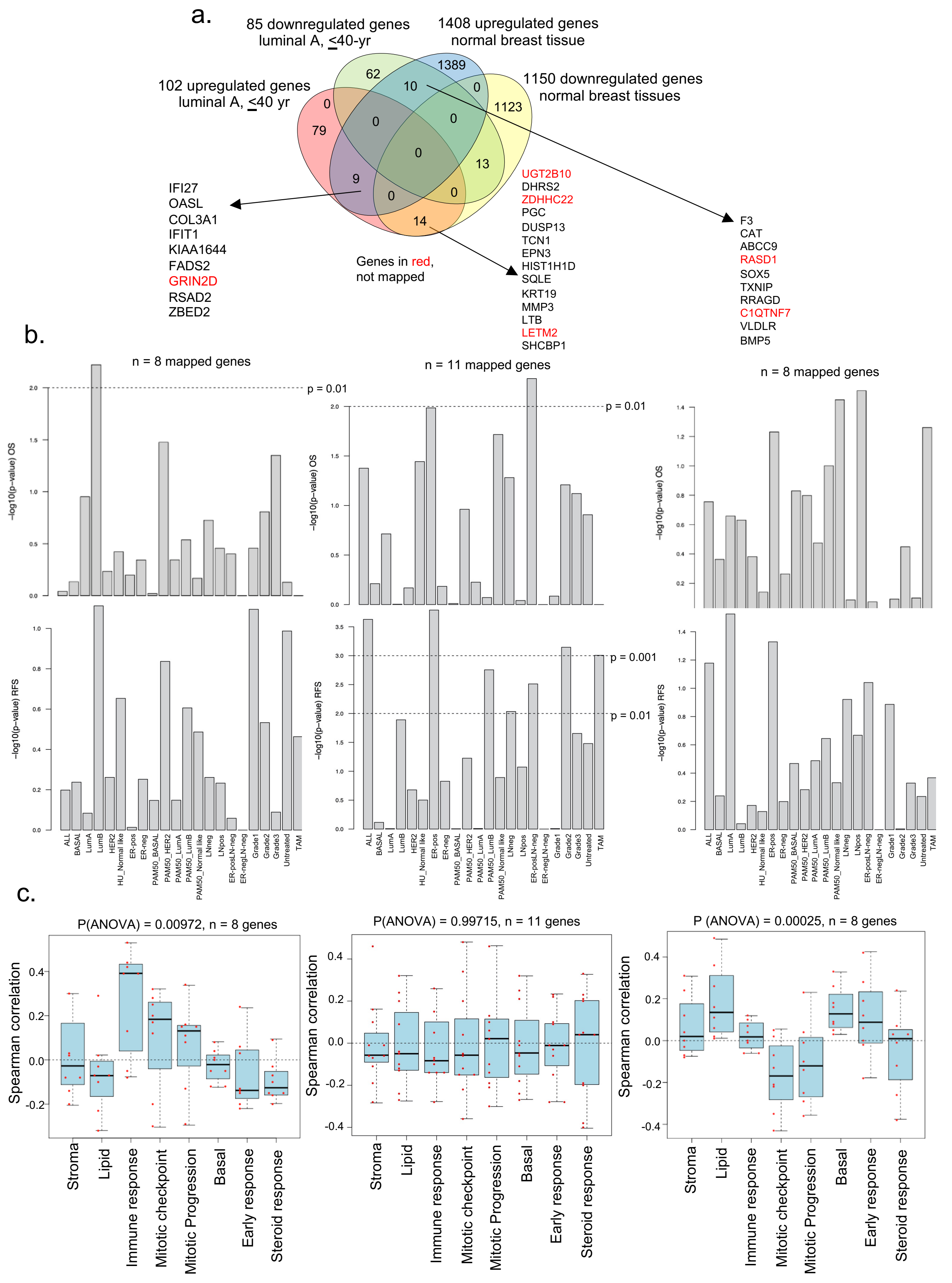

Supplementary Figure S11: Status of BCYW genes in intrinsic luminal A breast tumor subtype.

a, Overlap of up- and down-regulated genes in luminal A breast tumors from patients aged ≤40 years with upregulated or downregulated genes in normal breast tissues; **b**, Kaplan-Meier OS and RFS summaries of indicated 8 genes, 11 genes and 8 genes, respectively; **c**, Spearman correlation analysis of indicated 8 genes, 11 genes and 8 genes, respectively. All survival, correlation analysis performed using the online GOBO tools.

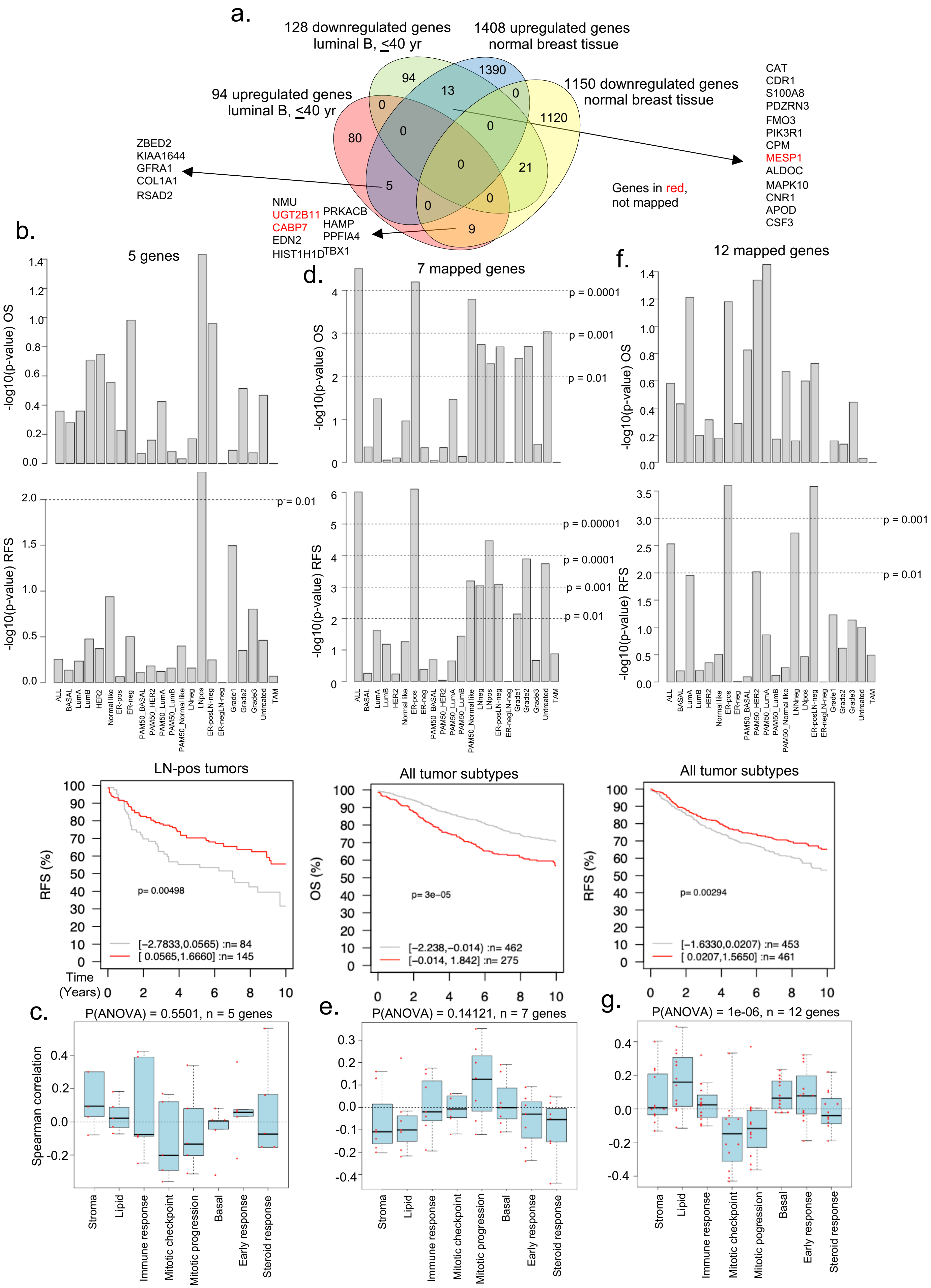

Supplementary Figure S12: Status of BCYW genes in intrinsic luminal B breast tumor subtype.

a, Overlap of up- and down-regulated genes in luminal B breast tumors from patients aged ≤40 year with upregulated or downregulated genes in normal breast tissues; **b**, Kaplan-Meier OS and RFS summaries of 5 genes, upregulated in luminal B breast tumors as well as in normal breast tissues; RFS curve using the high (red) and low (gray) expression of 5 genes in LN-pos breast tumors; **c**, Spearman correlation analysis of 5 genes in the context of co-expressed 8 gene modules; **d**, Kaplan-Meier OS and RFS summaries of 7 genes, upregulated in luminal B breast tumors but downregulated in normal breast tissues; OS curve using the high (red) and low (gray) expression of 7 genes in all tumor subtypes; **e**, Spearman correlation analysis of 7 genes in the context of co-expressed 8 gene modules; **f**, Kaplan-Meier OS and RFS summaries of 12 genes, downregulated in luminal B breast tumors but upregulated in normal breast tissues; RFS curve using the high (red) and low (gray) expression of 12 genes in all tumor subtypes; and **g**, Spearman correlation analysis of 12 genes in the context of co-expressed 8 gene modules. All survival, correlation analysis performed using the online GOBO tools.

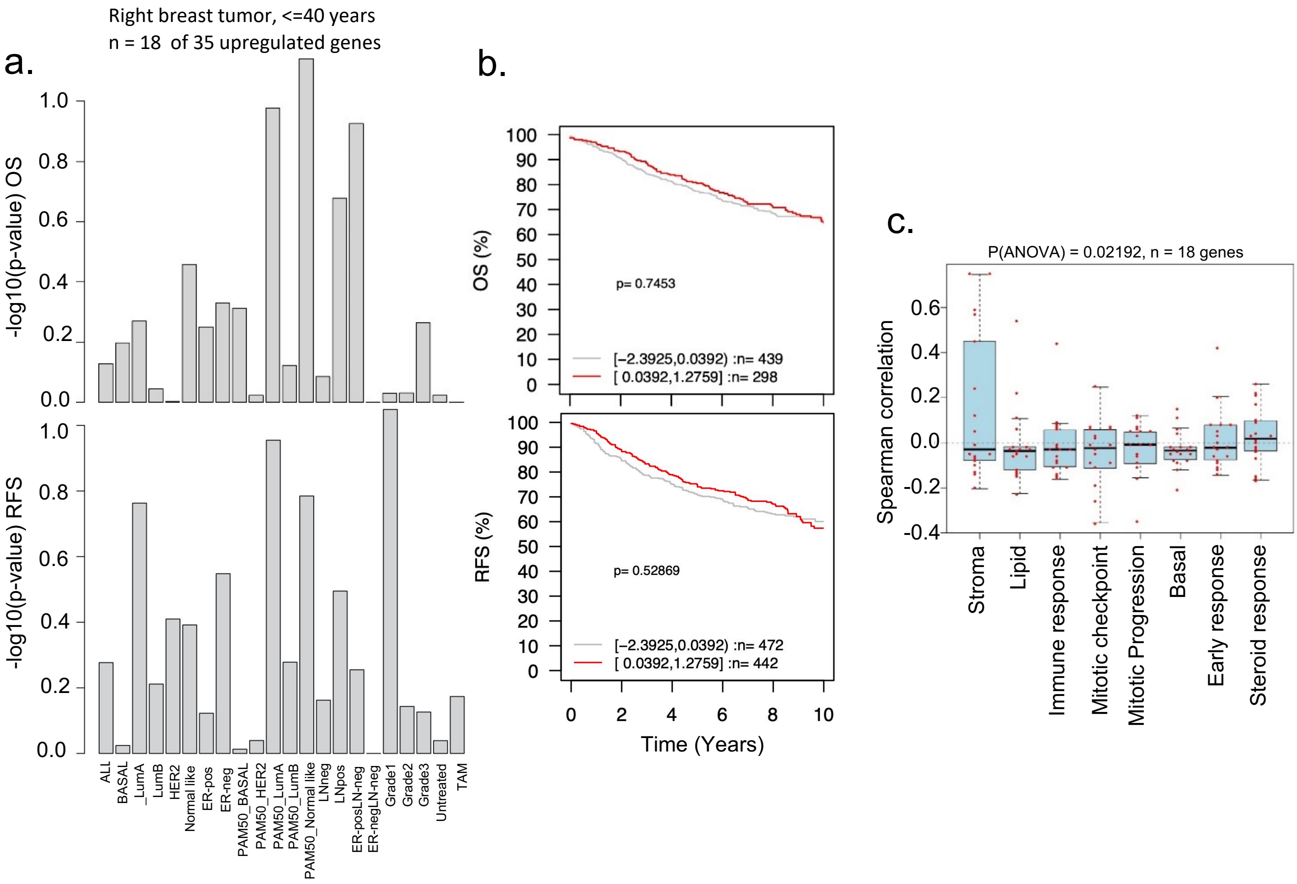

Supplementary Figure S13: Breast cancer genes in right breast tumors aged ≤40 years. **a**, Kaplan-Meier OS and RFS summaries of 18 of 35 upregulated genes in the right breast tumors from patients aged ≤40 years; **b**, OS and RFS curves using the high (red) and low (gray) expression of 18 genes in all breast tumor subtypes; and **c**, Spearman correlation analysis of 18 genes in the context of co-expressed 8 gene modules.

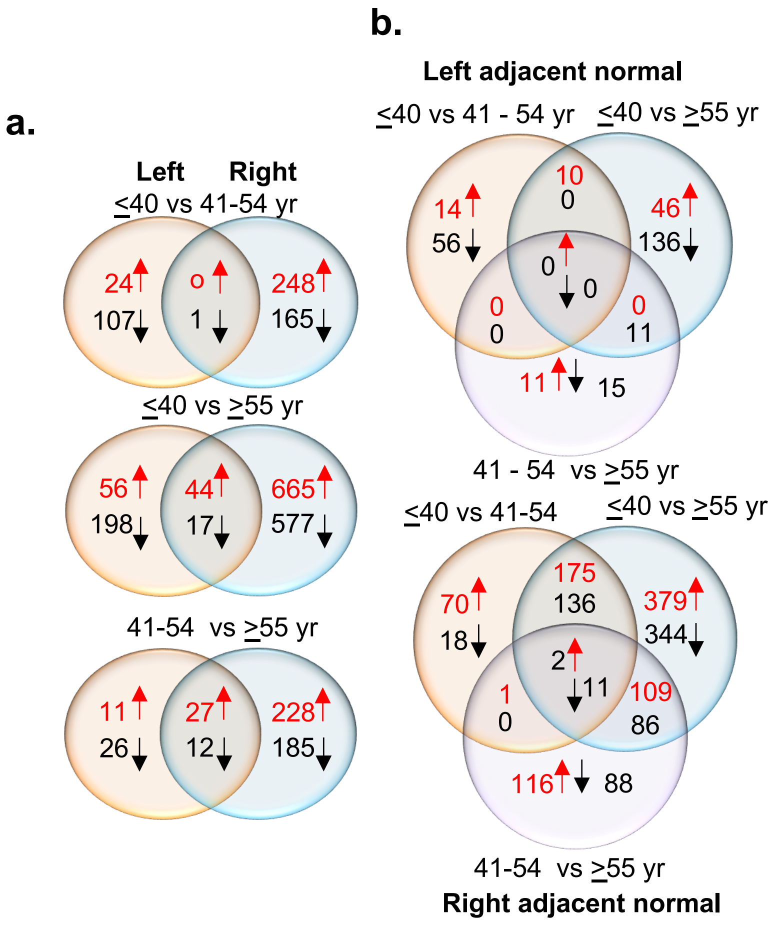

Supplementary Figure S14: Comparative analysis of dysregulated genes in matching normal specimens. **a**, Status of dysregulated DRG genes in the left and right adjacent normal tissues from three age groups; **b**, Overlap of dysregulated genes among ≤40 vs 41 - 54 years, ≤40 vs ≥55 years and 41 - 54 vs ≥ 55-years age groups of adjacent matching normal breast tissue samples. Red, upregulated, and blue, downregulated genes.

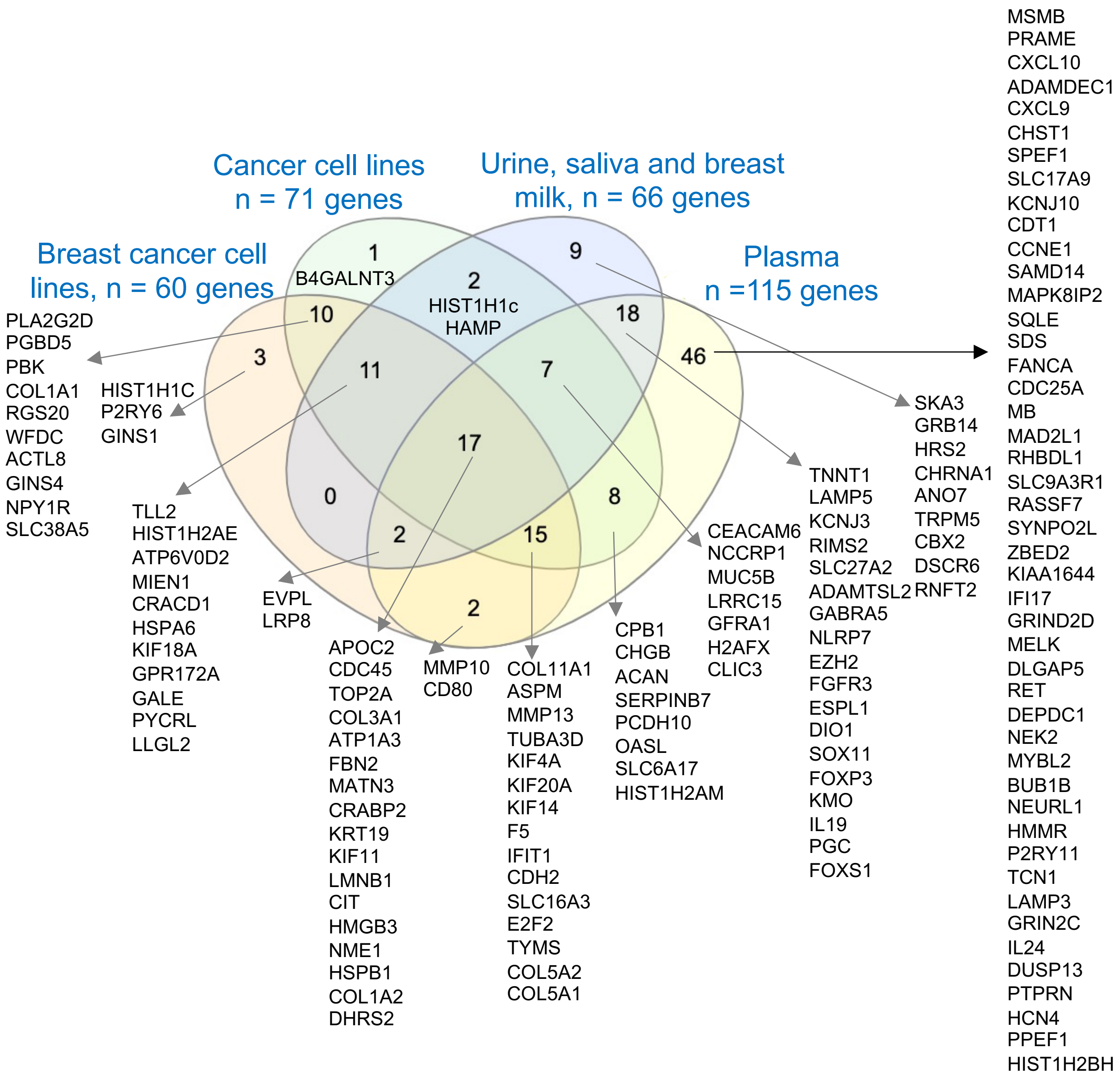

Supplementary Figure S15: Summary of exemplified comparative analyses to highlight the noticed overlap among secreted proteins by breast cancer cell lines, cancer cell lines, and three human body fluids such as urine, saliva and breast milk and plasma.

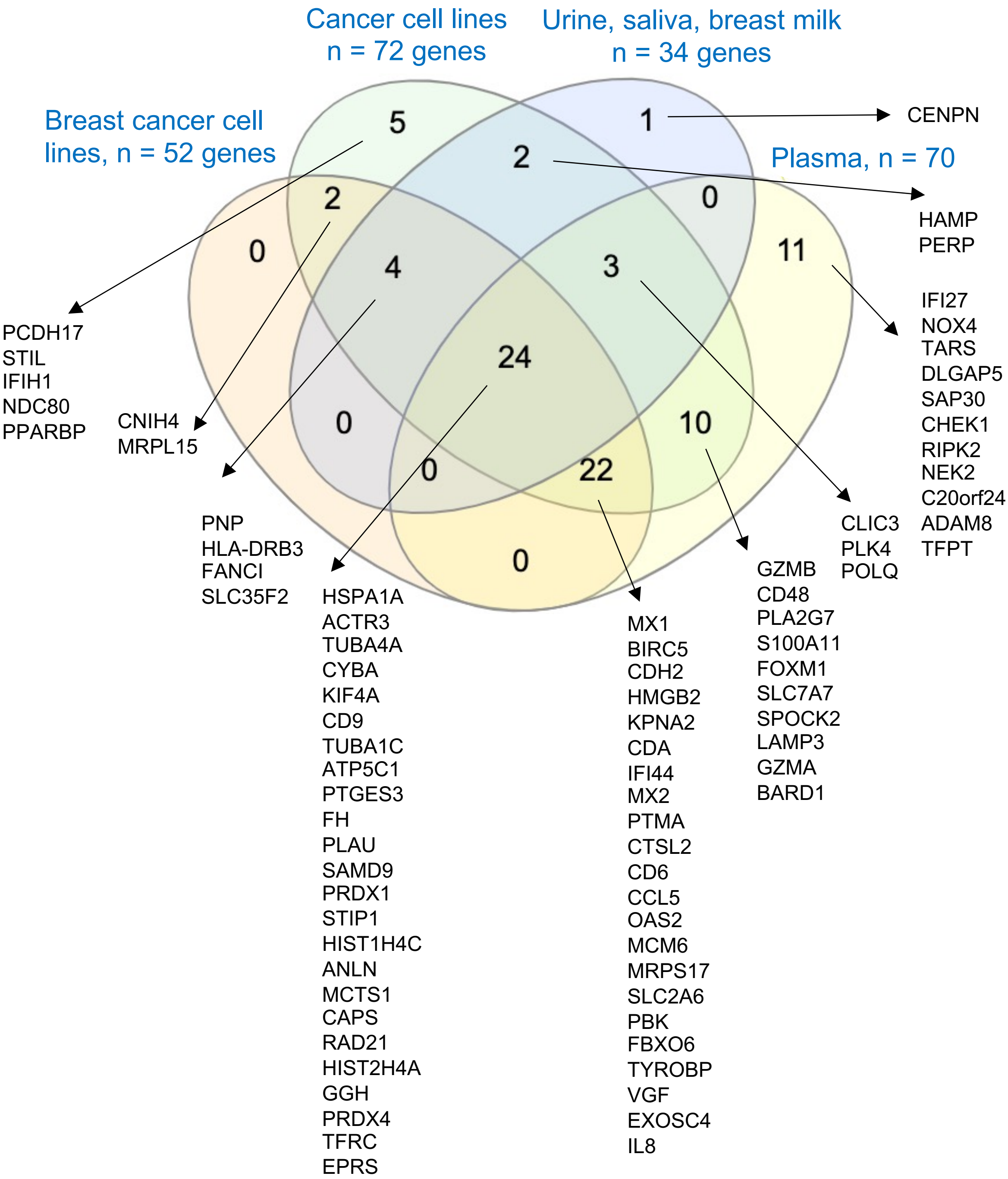

Supplementary Fig. S16: Summary and overlap of secreted proteins, identified in the METABRIC dataset for breast tumors aged ≤40 years, by breast cancer cell lines, cancer cell lines, human body fluids such as urine, saliva and breast milk, and plasma.

Supplementary Table S1: Summary of clinical features of the breast tumor specimens in the TCGA Firehose legacy breast cancer study.

Age Groups	<40 yrs.	41-54 yrs.	>55 yrs.
**Sample Numbers**	81	255	480
**PAM50 Subtype Classifications**			
Basal	16	53	70
HER2	3	25	39
LumA	42	108	267
LumB	18	50	120
**AJCC Stage**			
Stage I	25	13	40
Stage IA	6	20	38
Stage IB	1	2	8
Stage II	20	18	18
Stage IIA	20	71	158
Stage IIB	18	60	79
Stage III	4	12	2
Stage IIIA	17	24	61
Stage IIIB	0	4	19
Stage IIIC	3	9	20
Stage IV	0	5	10
Stage X	2	2	6
**Anatomic Neoplasm Subdivision**			
Right	9	52	93
Left	17	52	100
Right Upper	22	59	128
Right Lower	4	15	46
Left Upper	16	87	141
Left Lower	3	20	32
**Adjacent Matching Normal Samples**	12	37	59

Supplementary Table S2: Gene Ontology biological function analysis of 45 mapped genes out of 60 upregulated genes in Figure 1a.

Analysis: Biological process	
Dataset name: Group4	
Number of genes in the dataset: 60	
Not recognized for the analysis : 15	IER5L;B4GALNT3;H2AFX;C18orf56;KLHL35;KIAA1644;C4orf48;HAR1A;MROH6;CABP7;HIST1H2BH;HIST1H2AE;HIST1H2AM;C6orf223;C1orf105
Number of genes in the dataset (which are available in Biological process database) :45	COL5A2;COL5A1;COL1A2;TLL2;LLGL2;SLC6A17;FOXS1;SLC38A5;ANO7;CLIC3;CHRNA1;GABRA5;ADAMTSL2;KMO;HAMP;NLRP7;IL24;P2RY11;GRIN2C;TRPM5;HCN4;APOC2;PTPRN;PGBD5;PLA2G2D;LAMP3;F5;PPEF1;ZNF703;SLC27A2;RHBG;PGC;NPY1R;HSPB1;AMTN;DHRS2;ATP6V0D2;TCN1;CENPW;IL19;NME1;CHST8;MIEN1;DUSP13;C5orf46;
Number of genes in background: 17857	
Is analysis quantitative: No	

						Corrected p-values			
**Biological process**	**No. of genes in the dataset**	**No. of genes in the background dataset**	**Percentage of genes**	**Fold enrichment**	**P-value (Hypergeometric test)**	**Bonferroni method**	**BH method**	**Q-value (Storey-Tibshirani method)**	**genes mapped (from input data set)**
GO:1903225	2	2	4.44	396.73	0.00	0.08	0.07	0.49	COL5A2; COL5A1;
collagen fibril organization	4	56	8.89	28.40	0.00	0.15	0.07	0.49	COL1A2; COL5A2; TLL2; COL5A1;
eye morphogenesis	2	4	4.44	198.86	0.00	0.46	0.15	1.00	COL5A2; COL5A1;
leucine transport	2	5	4.44	159.17	0.00	0.77	0.19	1.00	LLGL2; SLC6A17;
blood vessel development	3	41	6.67	29.12	0.00	1.00	0.37	1.00	FOXS1; COL1A2; COL5A1;
glycine transport	2	9	4.44	88.51	0.00	1.00	0.46	1.00	SLC38A5; SLC6A17;
ion transmembrane transport	4	142	8.89	11.20	0.00	1.00	0.78	1.00	ANO7; CLIC3; CHRNA1; GABRA5;
extracellular matrix organization	4	156	8.89	10.20	0.00	1.00	0.96	1.00	COL1A2; COL5A2; ADAMTSL2; COL5A1;
cellular response to lipopolysaccharide	4	160	8.89	9.94	0.00	1.00	0.96	1.00	KMO; HAMP; NLRP7; IL24;
calcium-mediated signaling	3	81	6.67	14.74	0.00	1.00	1.00	1.00	P2RY11; GRIN2C; TRPM5;

Supplementary Table S3: Gene Ontology biological function analysis of 14 mapped genes out of highly upregulated 17 genes in Figure 1e.

Analysis: Biological process	
Dataset name: Group4	
Number of genes in the dataset: 17	
Not recognized for the analysis : 4	KLHL35;HAR1A;MROH6;C1ORF105
Number of genes in the dataset (which are available in Biological process database) :13	PGBD5;LAMP3;KMO;ZNF703;F5;RHBG;LLGL2;GRIN2C;IL24;ATP6V0D2;IL19;MIEN1;NME1;
Number of genes in background: 17857	
Is analysis quantitative: No	

						Corrected p-values			
Biological process	No. of genes in the dataset	No. of genes in the background dataset	Percentage of genes	Fold enrichment	P-value (Hypergeometric test)	Bonferroni method	BH method	Q-value (Storey-Tibshirani method)	genes mapped (from input data set)
GO:0098038	1	1	7.69	1372.56	0.00	1	1	1	PGBD5;
GO:1903900	1	1	7.69	1372.56	0.00	1	1	1	LAMP3;
kynurenic acid biosynthetic process	1	2	7.69	689.69	0.00	1	1	1	KMO;
GO:1903296	1	2	7.69	689.69	0.00	1	1	1	KMO;
negative regulation of homotypic cell-cell adhesion	1	2	7.69	689.69	0.00	1	1	1	ZNF703;
quinolinate biosynthetic process	1	3	7.69	460.56	0.00	1	1	1	KMO;
anthranilate metabolic process	1	3	7.69	460.56	0.00	1	1	1	KMO;
L-kynurenine metabolic process	1	3	7.69	460.56	0.00	1	1	1	KMO;
response to vitamin K	1	3	7.69	460.56	0.00	1	1	1	F5;
transepithelial ammonium transport	1	3	7.69	460.56	0.00	1	1	1	RHBG;

Supplementary Table S4: Gene Ontology biological function analysis of 19 mapped genes out of 22 upregulated genes in Figures 2b and 2c.

Analysis: Biological process	
Dataset name: Group1	
Number of genes in the dataset: 22	
Not recognized for the analysis : 3	NXPH1;MUC5B;C6orf223
Number of genes in the dataset (which are available in Biological process database) :19	FIBCD1;ACAN;PCDH10;CST9;SERPINB7;CPB1;HAMP;FOXS1;RIMS2;P2RY11;NCCRP1;DHRS2;CHGB;NPY1R;SLC6A17;LRRC15;TCN1;SYNPO2L;HS6ST3
Number of genes in background: 17857	
Is analysis quantitative: No	

						Corrected p-values			
**Biological process**	**No. of genes in the dataset**	**No. of genes in the background dataset**	**Percentage of genes**	**Fold enrichment**	**P-value (Hypergeometric test)**	**Bonferroni method**	**BH method**	**Q-value (Storey-Tibshirani method)**	**genes mapped (from input data set)**
response to erythropoietin	1	1	5.26	939.35	0.001064	1.00	1.00	1.00	HAMP;
GO:1904479	1	1	5.26	939.35	0.001064	1.00	1.00	1.00	HAMP;
positive regulation of platelet-derived growth factor production	1	2	5.26	472.01	0.002127	1.00	1.00	1.00	SERPINB7;
negative regulation of iron ion transmembrane transport	1	2	5.26	472.01	0.002127	1.00	1.00	1.00	HAMP;
GO:1990641	1	3	5.26	315.20	0.003189	1.00	1.00	1.00	HAMP;
positive regulation of glomerular mesangial cell proliferation	1	5	5.26	189.37	0.005309	1.00	1.00	1.00	SERPINB7;
positive regulation of cell growth involved in cardiac muscle cell development	1	5	5.26	189.37	0.005309	1.00	1.00	1.00	HAMP;
leucine transport	1	5	5.26	189.37	0.005309	1.00	1.00	1.00	SLC6A17;
GO:1903413	1	5	5.26	189.37	0.005309	1.00	1.00	1.00	HAMP;
alanine transport	1	5	5.26	189.37	0.005309	1.00	1.00	1.00	SLC6A17;

Supplementary Table S5: Gene Ontology biological function analysis of 8 mapped genes out of 12 downregulated genes in Figure 2g.

Analysis: Biological process	
Dataset name: Group3	
Number of genes in the dataset: 12	
Not recognized for the analysis : 4	VAT1L;COLCA1;SPHKAP;EMILIN3
Number of genes in the dataset (which are available in Biological process database) :8	TNFSF11;CSRNP3;PLPPR1;RSPO1;SLC26A3;SLCO1A2;SYCE1;IL22RA2
Number of genes in background: 17857	
Is analysis quantitative: No	

						Corrected p-values			
**Biological process**	**No. of genes in the dataset**	**No. of genes in the background dataset**	**Percentage of genes**	**Fold enrichment**	**P-value (Hypergeometric test)**	**Bonferroni method**	**BH method**	**Q-value (Storey-Tibshirani method)**	**genes mapped (from input data set)**
positive regulation of corticotropin-releasing hormone secretion	1	1	12.5	2229.34	0.000448	1	1	1	TNFSF11;
GO:1904616	1	1	12.5	2229.34	0.000448	1	1	1	TNFSF11;
positive regulation of fever generation by positive regulation of prostaglandin secretion	1	2	12.5	1120.215	0.000896	1	1	1	TNFSF11;
positive regulation of ERK1 and ERK2 cascade via TNFSF11-mediated signaling	1	2	12.5	1120.215	0.000896	1	1	1	TNFSF11;
cytokine-mediated signaling pathway	2	145	25	30.90113	0.001776	1	1	1	TNFSF11; IL22RA2;
osteoclast proliferation	1	4	12.5	561.5045	0.001791	1	1	1	TNFSF11;
positive regulation of osteoclast development	1	4	12.5	561.5045	0.001791	1	1	1	TNFSF11;
intracellular pH elevation	1	4	12.5	561.5045	0.001791	1	1	1	SLC26A3;
positive regulation of homotypic cell-cell adhesion	1	5	12.5	449.4277	0.002238	1	1	1	TNFSF11;
TNFSF11-mediated signaling pathway	1	5	12.5	449.4277	0.002238	1	1	1	TNFSF11;

Supplementary Table S6: Gene Ontology biological function analysis of 26 mapped genes out of 27 upregulated genes in Figure 3b.

Analysis: Biological process		
Dataset name: Group2		
Number of genes in the dataset:	27	
Not recognized for the analysis:	1	HMMR
Number of genes in the dataset (which are available in Biological process database) :26	SGO1;DLGAP5;NEK2;TOP2A;SKA3;TUBA3D;PBK;MYBL2;ESPL1;KIF4A;KIF20A;BUB1B;KIF14;MELK;RET;TNNT1;ASPM;CDC45;KCNJ3;COL11A1;CRACD;NEURL1;MMP13;LAMP5;SYT13;DEPDC1	
Number of genes in background:	17857	
Is analysis quantitative: No		

						Corrected p-values			
**Biological process**	**No. of genes in the dataset**	**No. of genes in the background dataset**	**Percentage of genes**	**Fold enrichment**	**P-value (Hypergeometric test)**	**Bonferroni method**	**BH method**	**Q-value (Storey-Tibshirani method)**	**genes mapped (from input data set)**
chromosome segregation	5	73	19.23	47.11	0.00	0.00	0.00	0.01	SGO1; DLGAP5; NEK2; TOP2A; SKA3;
mitotic cell cycle	5	132	19.23	26.06	0.00	0.01	0.01	0.05	TUBA3D; PBK; NEK2; MYBL2; SKA3;
mitotic sister chromatid segregation	3	33	11.54	62.60	0.00	0.18	0.06	0.41	SGO1; NEK2; ESPL1;
cytokinesis after mitosis	3	50	11.54	41.32	0.00	0.64	0.14	0.96	KIF4A; KIF20A; ESPL1;
homologous chromosome segregation	2	8	7.69	172.28	0.00	0.70	0.14	0.96	SGO1; ESPL1;
cell division	5	348	19.23	9.88	0.00	1.00	0.24	1.00	SGO1; NEK2; BUB1B; KIF14; SKA3;
positive regulation of mitotic metaphase/anaphase transition	2	12	7.69	114.90	0.00	1.00	0.24	1.00	DLGAP5; ESPL1;
peptidyl-tyrosine phosphorylation	3	130	11.54	15.89	0.00	1.00	1.00	1.00	MELK; RET; PBK;
GO:1905463	1	1	3.85	686.54	0.00	1.00	1.00	1.00	TOP2A;
negative regulation of muscle contraction	1	1	3.85	686.54	0.00	1.00	1.00	1.00	TNNT1;

Supplementary Table S7: Gene Ontology biological function analysis of 95 mapped genes out of 124 upregulated genes in Figure 7b (left breast cancer).

Analysis: Biological process	
Dataset name: Left<40_124Genes	
Not recognized for the analysis : 124	
Number of genes in the dataset: 29	TRY6;LOC400696;C2orf54;CNTD2;LINGO1;FAM64A;RDM1;C19orf33;RLTPR;DSCR6;NCRNA00176;C15orf42;MLF1IP;HIST1H2BE;C9orf140;NFKBIL2;ORC1L;KIAA1524;KIAA1211;C16orf75;PRR19;C3orf67;C20orf151;C18orf56;FCGR1C;GPR172A;PYCRL;C14orf80;RAG1AP1
Number of genes in the dataset (which are available in Biological process database) :95	IL4I1;FCGR1A;SRMS;IRF7;HMGB3;ADAMDEC1;HAMP;CD80;ESPL1;SAMD14;EZH2;CXCL10;LLGL2;RCC1;TMEM145;CXCL9;RAMP1;ADORA2A;P2RY6;HSPB1;TLX1;ZNF695;E2F2;FOXP3;FOXS1;SIX4;EVPL;PRAME;CEACAM6;SOX11;FGFR3;TNFRSF18;NUAK2;MAD2L1;SAMD11;CXCL11;MMP10;FAM111B;LRP8;RHBDL1;CDH2;CCNE1;MAPK8IP2;CRABP2;SLC9A3R1;TYMS;HSH2D;METRN;RASSF7;CAPS;CIT;JAKMIP1;KIF18A;POC1A;RNFT2;FBXO43;CBX2;CDC25A;MFSD2B;NME1;VSTM2L;MSMB;CHST1;SDS;LGALS9C;MND1;CDCA2;CDT1;KIF11;MATN3;MB;RAD51AP1;PIF1;SBK1;SPEF1;APOC2;SQLE;FANCA;PRR11;KCNJ10;PPM1J;ATP1A3;SLC17A9;KRT19;FBN2;ACTL8;IL21R;DIO1;LMNB1;GINS1;GINS4;IL17REL;SLC16A3;AIFM3;GALE
Number of genes in background: 17857	
Is analysis quantitative: No	

						Corrected p-values			
**Biological process**	**No. of genes in the dataset**	**No. of genes in the background dataset**	**Percentage of genes**	**Fold enrichment**	**P-value (Hypergeometric test)**	**Bonferroni method**	**BH method**	**Q-value (Storey-Tibshirani method)**	**genes mapped (from input data set)**
regulation of mitosis	3	23	3.16	24.59	0.000239	1.00	1.00	1.00	CDCA2; FBXO43; RCC1;
GO:1903934	2	5	2.11	75.40	0.000277	1.00	1.00	1.00	GINS1; GINS4;
adenylate cyclase-activating G-protein coupled receptor signaling pathway	5	121	5.26	7.78	0.000468	1.00	1.00	1.00	CXCL11; CXCL10; CXCL9; RAMP1; ADORA2A;
positive regulation of release of sequestered calcium ion into cytosol	3	29	3.16	19.50	0.000482	1.00	1.00	1.00	CXCL11; CXCL10; CXCL9;
GO:0097421	3	29	3.16	19.50	0.000482	1.00	1.00	1.00	HAMP; EZH2; TYMS;
cell division	8	348	8.42	4.33	0.000535	1.00	1.00	1.00	CDCA2; CDT1; CCNE1; KIF11; CDC25A; MAD2L1; LLGL2; RCC1;
positive regulation of phospholipase activity	2	7	2.11	53.89	0.000578	1.00	1.00	1.00	FGFR3; APOC2;
mitotic sister chromatid segregation	3	33	3.16	17.14	0.000709	1.00	1.00	1.00	ESPL1; KIF18A; MAD2L1;
neuroepithelial cell differentiation	2	8	2.11	47.16	0.000768	1.00	1.00	1.00	SOX11; CDH2;
negative regulation of retinoic acid receptor signaling pathway	2	9	2.11	41.93	0.000984	1.00	1.00	1.00	PRAME; EZH2;

Supplementary Table S8: Gene Ontology biological function analysis of 11 highly upregulated genes in Figure 7f.

Analysis: Biological process	
Number of genes in the dataset: 11	
Number of genes in the dataset (which are available in Biological process database) :11	SLC50A1;KCNJ10;FCGR1A;CRABP2;SLC52A2;PYCR3;FBXO43;SQLE;TONSL;NUAK2;ZNF695
Number of genes in background: 17857	
Is analysis quantitative: No	

						Corrected p-values			
**Biological process**	**No. of genes in the dataset**	**No. of genes in the background dataset**	**Percentage of genes**	**Fold enrichment**	**P-value (Hypergeometric test)**	**Bonferroni method**	**BH method**	**Q-value (Storey-Tibshirani method)**	**genes mapped (from input data set)**
cellular aromatic compound metabolic process	1	1	9.09	1621.89	0.000616	1.00	1.00	1.00	SQLE;
glucoside transport	1	1	9.09	1621.89	0.000616	1.00	1.00	1.00	SLC50A1;
glutamate uptake involved in synaptic transmission	1	2	9.09	814.98	0.001232	1.00	1.00	1.00	KCNJ10;
antibody-dependent cellular cytotoxicity	1	3	9.09	544.22	0.001847	1.00	1.00	1.00	FCGR1A;
retinoic acid biosynthetic process	1	5	9.09	326.97	0.003077	1.00	1.00	1.00	CRABP2;
riboflavin metabolic process	1	5	9.09	326.97	0.003077	1.00	1.00	1.00	SLC52A2;
L-proline biosynthetic process	1	6	9.09	272.56	0.003691	1.00	1.00	1.00	PYCR3;
negative regulation of meiosis	1	6	9.09	272.56	0.003691	1.00	1.00	1.00	FBXO43;
riboflavin transport	1	6	9.09	272.56	0.003691	1.00	1.00	1.00	SLC52A2;
positive regulation of collateral sprouting	1	8	9.09	204.51	0.004918	1.00	1.00	1.00	CRABP2;

**Supplementary Table S9**: Examples of secreted gene products relevant to the biology of BCYW, aged ≤40 years.	
**A. Upregulated genes in BC ≤40 years, Fig. 1a**	
Breast cancer cells	LLGL2, F5, NME1, COL1A2, HSPB1, COL5A2, PGBD5, TLL2, COL5A1, NPY1R, PLA2G2D, HIST1H2AE, DHRS2, SLC38A5, ATP6V0D2, APOC2
Cancer cell lines	LLGL2, F5, NME1, COL1A2, HSPB1, COL5A2, PGBD5, TLL2, COL5A1, NPY1R, PLA2G2D, HIST1H2AE, DHRS2, H2AFX, HAMP, CLIC3, HIST1H2AM, SLC38A5, ATP6V0D2, APOC2, B4GALNT3, SLC6A17
Urine	LLGL2, NME1, COL1A2, HSPB1, H2AFX, KMO, SLC27A2, ADAMTSL2, HAMP, CLIC3, CHRNA1, HIST1H2AE, PGC, DHRS2, GABRA5, ANO7, FOXS1, ATP6V0D2, NLRP7
Saliva	NME1, HSPB1, HIST1H2AE
Breast milk	LLGL2, NME1, HSPB1, TLL2, HAMP, IL19
Proteome	LAMP3, F5, NME1, COL1A2, HSPB1, H2AFX, COL5A2, KMO, SLC27A2, ADAMTSL2, P2RY11, COL5A1, PTPRN, CLIC3, GRIN2C, IL24, HCN4, PPEF1, TCN1, DUSP13, PGC, HIST1H2AM, DHRS2, GABRA5, FOXS1, SLC6A17, APOC2, NLRP7, HIST1H2BH
**B. Subset of highly upregulated genes in BC ≤40 years, Fig. 1e**	
Breast cancer cells	MIEN1, LLGL2, F5, NME1, PGBD5, ATP6V0D2
Cancer cell lines	MIEN1, LLGL2, F5, NME1, PGBD5, ATP6V0D2
Urine	MIEN1, LLGL2, NME1, KMO, ATP6V0D2
Saliva	NME1
Breast milk	IL19, MIEN1, NME1, LLGL2
Proteome	LAMP3, F5, NME1, KMO, GRIN2C, IL24, IL19
**C. Upregulated genes in BC but downregulated in adjacent normal, Fig. 2b,c**	
Breast cancer cells	DHRS2, NCCRP1, CHGB, NPY1R, ACAN, MUC5B, LRRC15
Cancer cell lines	DHRS2, SLC6A17, CPB1, NCCRP1, CHGB, NPY1R, ACAN, SERPINB7, HAMP, MUC5B, LRRC15, PCDH10
Urine	HRS2, NCCRP1, RIMS2, HAMP, FOXS1, MUC5B, LRRC15
Saliva	NCCRP1, MUC5B
Breast milk	NCCRP1, MUC5B, HAMP
Proteome	DHRS2, TCN1, SLC6A17, CPB1, NCCRP1, RIMS2, CHGB, SYNPO2L, ACAN, SERPINB7, FOXS1, MUC5B, LRRC15, P2RY11, PCDH10
**D. Upregulated genes in BC 3-age groups and adjacent normal DRG ≤40vs≥55 years, Fig. 3a**	
Breast cancer cells	COL11A1, ASPM, MMP13, CDC45, TUBA3D, PBK, CRACD1, KIF4A, KIF20A, TOP2A, KIF14
Cancer cell lines	COL11A1, ASPM, MMP13, CDC45, TUBA3D, PBK, CRACD1, KIF4A, KIF20A, TOP2A, KIF14, CDC45, TUBA3D
Urine	TNNT1, LAMP5, CDC45, CRACD1, TOP2A, KCNJ3, SKA3
Proteome	TNNT1, COL11A1, LAMP5, ASPM, MMP13, MELK, DLGAP5, RET, CDC45, TUBA3D, DEPDC1, KIF4A, NEK2, MYBL2, KIF20A, BUB1B, NEURL1, TOP2A, HMMR, KCNJ3, KIF14, ESPL1
**E. Upregulated genes in BC ≤40 year and normal breast tissues, Fig. 4a**	
Breast cancer cells	COL1A2, COL5A2, COL5A1, NPY1R
Cancer cell lines	COL1A2, COL5A2, COL5A1, NPY1R
Urine	COL1A2
Proteome	COL1A2, COL5A2, COL5A1
**F. Upregulated genes in BC ≤40 year but downregulated in normal breast tissues, Fig. 4b**	
Breast cancer cells	F5, SLC38A5, DHRS2
Cancer cell lines	F5, SLC38A5, DHRS2, HAMP
Urine	HAMP, PGC, DHRS2
Breast milk	HAMP
Proteome	LAMP3, F5, TCN1, DUSP13, PGC, DHRS2

G. Upregulated genes in basal BC ≤40 years and in normal breast tissues, Fig. 6c	
Breast cancer cells	HSPA6, RGS20, WFDC, HIST1H1c
Cancer cell lines	HSPA6, RGS20, WFDC, HIST1H1C, GRB14
Urine	HSPA6, GRB14, HIST1H1C
Saliva	HSPA6
Breast milk	HIST1H1c
**H. Upregulated genes in LumA and LumB BC ≤40 years and normal breast tissues, Supplementary Fig. 10 and 11**	
Breast cancer cells	COL1A1, COL3A1, IFIT1
Cancer cell lines	GFRA1, COL1A1, OASL, COL3A1, IFIT1
Urine	GFRA1, COL1A1,
Saliva	COL3A1
Proteome	ZBED2, KIAA1644, GFRA1, IFI17, OASL, COL3A1, IFIT1GRIND2D, ZBED2
**I. Downregulated in HER2 BC ≤40 years and upregulated in normal breast tissues, Supplementary Fig. 9a**	
Breast cancer cells	TTN, GAS6, GAS7, SGCB, SPTBN1, KCTD12, FABP5, PVRL3, ARHGEF6, PIK3R1, DPP4, CD1C, F13A1, STXBP6, APOD
Cancer cell lines	TTN, COLEC12, GAS6, PKD2, GAS7, NEDD9, SGCB, FGFR1, HOXA10, TACC1, SPTBN1, PPP1R16B, KCTD12, FABP5, PVRL3, ARHGEF6, PIK3R1, DPP4, CD1C, KCNK3, F13A1, STXBP6, APOD, GFRA1
Urine	TTN, COLEC12, GAS6, PKD2, GAS7, NEDD9, SGCB, FGFR1, HOXA10, TACC1, SPTBN1, PPP1R16B, KCTD12, FABP5, PVRL3, ARHGEF6, PIK3R1, DPP4, CD1C, KCNK3, F13A1, STXBP6, APOD, GFRA1
Saliva	FGFR1, DPP4
Breast milk	FGFR1, SPTBN1, FABP5, APOD
Proteome	TTN, CCNT1, KLF9, BTG2, TEF, COLEC12, GAS6, PLCL2, PKD2, GAS7, NEDD9, SGCB, FGFR1, ZNF423, TEAD1, HOXA10, DIO3, TACC1, SPTBN1, ADRBK2, PPP1R16B, KCTD12, FABP5, PVRL3, ARHGEF6, CYP7B1, PIK3R1, DPP4, GLRB, IRAK3, KCNK3, F13A1, CYP4F12, NAV3, HOXA9, APOD, GFRA1
**J. Upregulated genes in left BC ≤40 years, Fig. 7b left**	
Breast cancer cells	ACTL8, CEACAM6, ATP1A3, FBN2, MATN3, CDH2, CRABP2, SLC16A3, E2F2, KRT19, KIF18A, KIF11, LMNB1, HSPB1, CIT, GINS4, LLGL2, HMGB3, GPR172A, GALE, PYCRL, TYMS, NME1
Cancer cell lines	ACTL8, MMP10, ATP1A3, FBN2, MATN3, CDH2, CRABP2, CD80, SLC16A3, E2F2, KRT19, KIF18A, KIF11, LMNB1, P2RY6, EVPL, HSPB1, GINS1, LRP8, CIT, GINS4, LLGL2, HMGB3, GPR172A, GALE, PYCRL, TYMS, NME1
Urine	CBX2, FGFR3, ESPL1, DSCR6, CRABP2, KRT19, KIF18A, KIF11, RNFT2, LMNB1, EVPL, HSPB1, EZH2, CIT, LLGL2, HMGB3, GALE, NME1, CEACAM6, DIO1, SOX11, ATP1A3, FBN2, MATN3
Saliva	CEACAM6, KRT19, EVPL, NME1
Breast milk	MATN3, CRABP2, KRT19, EVPL, HSPB1, LLGL2, GPR172A, GALE, PYCRL, NME1
Proteome	TTN, CCNT1, KLF9, BTG2, TEF, COLEC12, GAS6, PLCL2, PKD2, GAS7, NEDD9, SGCB, FGFR1, ZNF423, TEAD1, HOXA10, DIO3, TACC1, SPTBN1, ADRBK2, PPP1R16B, KCTD12, FABP5, PVRL3, ARHGEF6, CYP7B1, PIK3R1, DPP4, GLRB, IRAK3, KCNK3, F13A1, CYP4F12, NAV3, HOXA9, APOD, GFRA1

Supplementary Table S10: Summary of key observations in the present study. Emply blocks in OS and RFS, no significant p-value; ND, not determined; NS, not significant; pos, positive correlation; Neg, negative correlation.

	Upregulated genes BC≤40 yr	Highly Upregulated ≤40 yr	Up BC ≤40 years Down in adjacent normal	Up BC Up adjacent normal ≤40 vs ≥55 yr	Up BC ≤40 yr common Up normal breast	Up BC ≤40 yr common Down normal breast	Down BC <40 yr common Up normal breast	Up basal ≤40 yr common Up normal breast	Down basal ≤40 yr common Up normal breast	Up HER2 ≤40 yr commom Up normal breast	Down HER2 ≤40 yr commom Up normal breast	Up LumA ≤40 yr commom Up normal breast	Down LumA ≤40 yr commom Up normal breast	Up LumB ≤40 yr commom Up normal breast	Up LumB ≤40 yr common Down normal breast	Down LumB ≤40 yr common Up normal breast	Up ≤40 yr Left Tumors
**GENESET**	60	60	22	27	60	60	58	132	194	161	552	102	85	94	94	128	124
Subset gene number		17			5	28	10	11	22	6	52	9	10	5	9	13	
Genes mapped	38	11	12	19	5	17	9	10	21	4	44	8	8	5	7	12	76
**OVERALL SURVIVAL**																	
p-value significant	Yes	No	Yes	Yes	Yes	Yes	No	Yes	Yes	No	Yes	No	No	No	Yes	No	Yes
All Tumors	0.82859	No	0.56243	<0.00001	0.00072	0.02739		0.14248	0.00116		0.01896				3.00E-05		1.00E-05
Basal								0.0512	0.47058								
Lum A				0.00141				0.00624									
Lum B																	
ER+				<0.00001	0.0056	0.00497		0.00431	0.00424						6.00E-05		<0.00001
LN-				<0.00001											0.00183		0.00029
LN+			0.00169	4.00E-05					0.00056		2.20E-04				0.00508		
ER-posLN-neg	0.00204		0.00783	<0.00001		0.00825		0.00436							0.00207		4.00E-05
Pam50 normal-like			0.00664			0.00335									0.00016		
Grade 1			0.00754												0.00384		
Grade 2				<0.00001	0.00497										0.00202		9.00E-05
Grade 3																	
**RELAPSE FREE SURVIVAL**																	
p-value significant	No	Yes	Yes	Yes	Yes	Yes	Yes	No	Yes	No	Yes	No	No	Yes	Yes	Yes	Yes
All tumors	No	0.06891	0.03584	<0.00001	<0.00001	0.00323	0.00013	0.36986	<0.00001		1.00E-05			No	<0.00001	0.00294	0.00001
Basal		0.0095				0.01217			0.25768								
Lum A				0.0031	0.00031												
Lum B				0.00131		0.00583											0.00698
ER+		0.00828		<0.00001	6.00E-05	4.00E-05	0.00024		1.00E-05		0.00029				<0.00001	0.00025	<0.00001
LN-				<0.00001	0.00094		0.00137		<0.00001		0.00196				9.00E-04	0.00188	0.00014
LN+			0.00775	1.00E-05	0.00015						5.00E-04			0.00498	3.00E-05		0.01095
ER-posLN-neg				<0.00001	0.00488	0.00452	0.00342		1.00E-05						0.00081	0.00026	4.00E-05
Pam50 Normal-like															0.00064		
Grade 1				0.00099	0.00957										0.00718		
Grade 2		0.00147		<0.00001	0.00178		0.00689				0.00023				0.00013		0.00111
Grade 3									0.00321								
**CORRELATION with 8 GENE MODULES**																	
P(ANOVA)	0.16715	0.10505	1.00E-05	<0.00001	<0.00001	0.10291	<0.00001	0.05231	<0.00001	No	<0.00001	0.00972	0.00025	0.5501	No	1.00E-06	<0.00001
Stroma	NS	NS	pos	neg	pos	NS	pos	neg	pos	NS	pos	neg	NS	pos	NS	NS	neg
Lipid	NS	NS	pos	neg	pos	NS	pos	neg	pos	NS	pos	neg	pos	NS	NS	pos	neg
Immune	NS	NS	neg	pos	neg	NS	neg	pos	neg	NS	NS	pos	NS	neg	NS	N.S.	pos
Mitotic	NS	NS	neg	pos	neg	NS	neg	NS	neg	NS	neg	pos	neg	neg	NS	neg	pos
Basal	NS	NS	neg	neg	NS	NS	pos	pos	pos	NS	pos	neg	pos	NS	NS	pos	neg
Early	NS	NS	pos	neg	pos	NS	pos	neg	pos	NS	pos	neg	pos	NS	NS	pos	neg
Steroid	NS	NS	pos	neg	pos	NS	pos	neg	pos	NS	pos	neg		neg	NS	neg	neg
**SECRETION**	**SECRETION**	**SECRETION**	**SECRETION**	**SECRETION**	**SECRETION**	**SECRETION**	**ND**	**SECRETION**	**ND**	**ND**	**ND**	**SECRETION**	**ND**	**ND**	**ND**	**ND**	**SECRETION**
Breast cancer cells	16	6	7	11	4	3		4				3					23
Cancer cells	22	6	12	13	4	4		5				5					28
Urine	19	5	7	7	1	3		3				2					24
Saliva	3	1	2	none	none	none		1				1					4
Breast milk	6	4	3	none	none	1		1				none					10
Plasma	29	7	15	22	3	6		none				8					47
